# Limited detection of Gulf War illness-like behavioral phenotypes in Charles River male Sprague-Dawley rats across multiple chemical and stress exposure paradigms

**DOI:** 10.1371/journal.pone.0337157

**Published:** 2026-09-30

**Authors:** Rashelle Lashley, Joshua Brian Foster, Xueqin Wang, Albert John Muhleman, Samuel Weaver, Edison Lin, Liching Lai, Chien-liang Glenn Lin

**Affiliations:** Department of Neuroscience, College of Medicine, The Ohio State University, Columbus, Ohio, United States of America; University of Tartu, ESTONIA

## Abstract

Gulf War illness (GWI) is characterized by a range of neurological and neuropsychiatric symptoms, including chronic pain, fatigue, mood problems, and cognitive impairment. The pathophysiology is associated with war-related environmental chemical exposure and psychological stress, which are known to disrupt glutamatergic synaptic structure and function. The original objective of this study was to evaluate the efficacy of LH-001, an investigational compound designed to enhance tripartite glutamatergic synapses, in a rat model of GWI. We initially attempted to induce GWI-like behavioral phenotypes in Charles River male Sprague–Dawley (SD) rats using a previously published protocol employed for male SD rats from Harlan involving exposure to Gulf War-related chemical agents and mild stress. Because robust behavioral phenotypes were not detected, we subsequently modified the induction protocol by increasing chemical exposure intensity and stress levels. Despite these adjustments, GWI-like behavioral phenotypes were not detected under the experimental conditions evaluated. To better interpret these findings, we compared our protocol and outcomes with previously published GWI rat studies. This comparison revealed substantial variability across studies in exposure paradigms, animal sources, timing of behavioral assessments, and reported outcomes. Collectively, our findings suggest that detection of GWI-like behavioral phenotypes in rats may be sensitive to experimental conditions and highlight the importance of continued efforts to validate, optimize, and standardize animal models to improve translational rigor and support preclinical therapeutic development.

## Introduction

Gulf War illness (GWI) is a chronic, multi-symptomatic condition affecting approximately one-third of veterans—around 250,000 people—who served in the 1990–1991 Gulf War [1]. GWI presents a wide range of symptoms that include fatigue, gastrointestinal disorders, and widespread pain, among others [2]. Neurological problems, including headache, sleep difficulty, anxiety, depression, memory deficit, and difficulty in concentration, are commonly reported symptoms [3–6]. Although the exact etiology of the disease remains unclear, it is widely believed that these clinical symptoms stem from a combination of war-related stress and exposure to a range of toxic chemical agents [7–10]. These chemical agents include pyridostigmine bromide (PB, administered as a prophylactic treatment against organophosphate nerve agents); pesticides (such as permethrin (PER) and N,N-diethyl-meta-toluamide (DEET)); chemical warfare agents (such as sarin); burn pits; byproducts of oil well fires; and depleted uranium. Current treatments for the complex disorder of GWI focus on managing symptoms; however, understanding its mechanisms is critical for developing more effective therapies.

Growing evidence indicates that exposure to GW-related chemical agents can impair the structure and function of glutamatergic synapses in the central nervous system [11]. PB and certain Gulf War-related neurotoxic exposures, including organophosphate nerve agents such as sarin, have been associated with inhibition of acetylcholinesterase activity [12]. Under stress conditions, these exposures have been proposed to alter central neurotransmission and contribute to dysregulation of glutamatergic signaling, excitotoxicity, and synaptic dysfunction [13,14]. Air pollutants associated with burn pit exposure have also been proposed to contribute to neuroinflammation, oxidative stress, excitotoxicity, and disruption of glutamatergic signaling following systemic exposure [15,16]. Depleted uranium exposure has been associated with altered glutamate uptake, changes in glutamate receptor expression, neurochemical imbalance, oxidative stress, and neuroinflammation [17]. Moreover, mounting evidence indicates that stress can cause dysregulation of glutamate transmission and losses of dendrites and their synapses [18,19]. Taken together, military-related toxic exposures may impair glutamatergic synapses, resulting in long-term structural and functional alterations in the brain and leading to neurological problems.

Because synaptic dysfunction has been implicated in proposed mechanisms underlying GWI, we previously evaluated LDN/OSU-215111, a compound designed to enhance the structure and function of tripartite glutamatergic synapses, in a mouse model of GWI induced by Gulf War-related chemicals and stress. In that study, LDN/OSU-215111 improved behavioral deficits and normalized associated hippocampal abnormalities [20]. Subsequent studies identified LH-001, a metabolite of LDN/OSU-215111 with improved preclinical pharmacological properties.

The original objective of the present study was to evaluate the effects of LH-001 in a rat model of GWI and determine whether enhancing tripartite glutamatergic synaptic function could influence GWI-related outcomes. To establish the model, we first applied a previously published rat exposure protocol developed for male Harlan-SD rats involving Gulf War-related chemicals and restraint stress, using Charles River SD rats [21]. Because expected GWI-like behavioral phenotypes were not observed, additional exposure paradigms incorporating modified chemical and stress conditions were subsequently evaluated. However, behavioral phenotypes consistent with GWI were still not detected under the experimental conditions used. Therefore, conclusions regarding the therapeutic efficacy of LH-001 cannot be drawn from the present study. Potential factors that may contribute to variability in GWI phenotype detection across experimental conditions and prior studies are discussed.

## Materials and methods

### Animals

Male Sprague-Dawley rats were obtained from Charles River Laboratories (Raleigh, NC, USA) and used in all cohorts. Rat starting weights ranged from 225–275 g, at an estimated age of 7–8 weeks. Rats were left to habituate in the vivarium for one week before beginning the stress and chemical exposure protocol. Vivarium conditions were kept at a steady temperature (approximately 70°F) with a 12-hour room light/dark cycle. Rats were housed in groups or pairs depending on size and provided ad libitum access to food and water, except on days that required food or water deprivation. All procedures were done between 8 am and 6 pm. All procedures were approved by the Institutional Animal Care and Use Committee of The Ohio State University following the National Institutes of Health Guide for the Care and Use of Laboratory Animals.

### Experimental design

In the original study design, we planned to use 24 rats of each sex per group, based on an assumed large effect size (Cohen’s d = 0.8), a significance level (α) of 0.05, and 80% statistical power, following procedures adapted from Parihar et al. [21]. The study was designed to include four experimental cohorts, each consisting of 12 male or female rats per group.

We initiated the study with Cohort 1, which included 48 male rats (12 rats per group). Animals were randomly assigned to one of four groups: control/vehicle (no GW exposure, vehicle treatment), control/LH-001 (no GW exposure, LH-001 treatment), GWI/vehicle (GW exposure, vehicle treatment), and GWI/LH-001 (GW exposure, LH-001 treatment). Rats in the GWI groups were exposed to GW-related chemicals (1.3 mg/kg PB, 0.13 mg/kg PER, and 40 mg/kg DEET) followed by 5-minute restraint stress on weekdays for five weeks (hereinafter referred to as the “low-dose/low-stress” protocol). Body weight was monitored weekly during the first six weeks.

Beginning 4–5 weeks after completion of GW exposure, rats received either LH-001 or vehicle throughout the behavioral assessment period. Behavioral testing was initiated 8–9 weeks after exposure and included evaluation of anxiety-like behaviors (open field, light–dark transition, elevated plus maze, and novelty-suppressed feeding) and cognition (novel object location, novel object recognition, and Barnes maze). Investigators performing behavioral assessments were blinded to group allocation.

Following completion of Cohort 1, expected GWI-like behavioral phenotypes were not detected (Fig 1). Therefore, rather than proceeding directly with the remaining planned cohorts, we conducted additional exploratory studies to evaluate whether modifying exposure conditions would influence phenotype detection. First, we increased chemical exposure (13 mg/kg PB, 2.6 mg/kg PER, and 215 mg/kg DEET) while maintaining 5-minute restraint stress (“high-dose/low-stress” protocol) [22,23]. Subsequently, we evaluated an alternative exposure paradigm adapted from our previous mouse study [20], which used the original chemical doses combined with two unpredictable stressors (“low-dose/high-stress” protocol).

**Fig 1:**
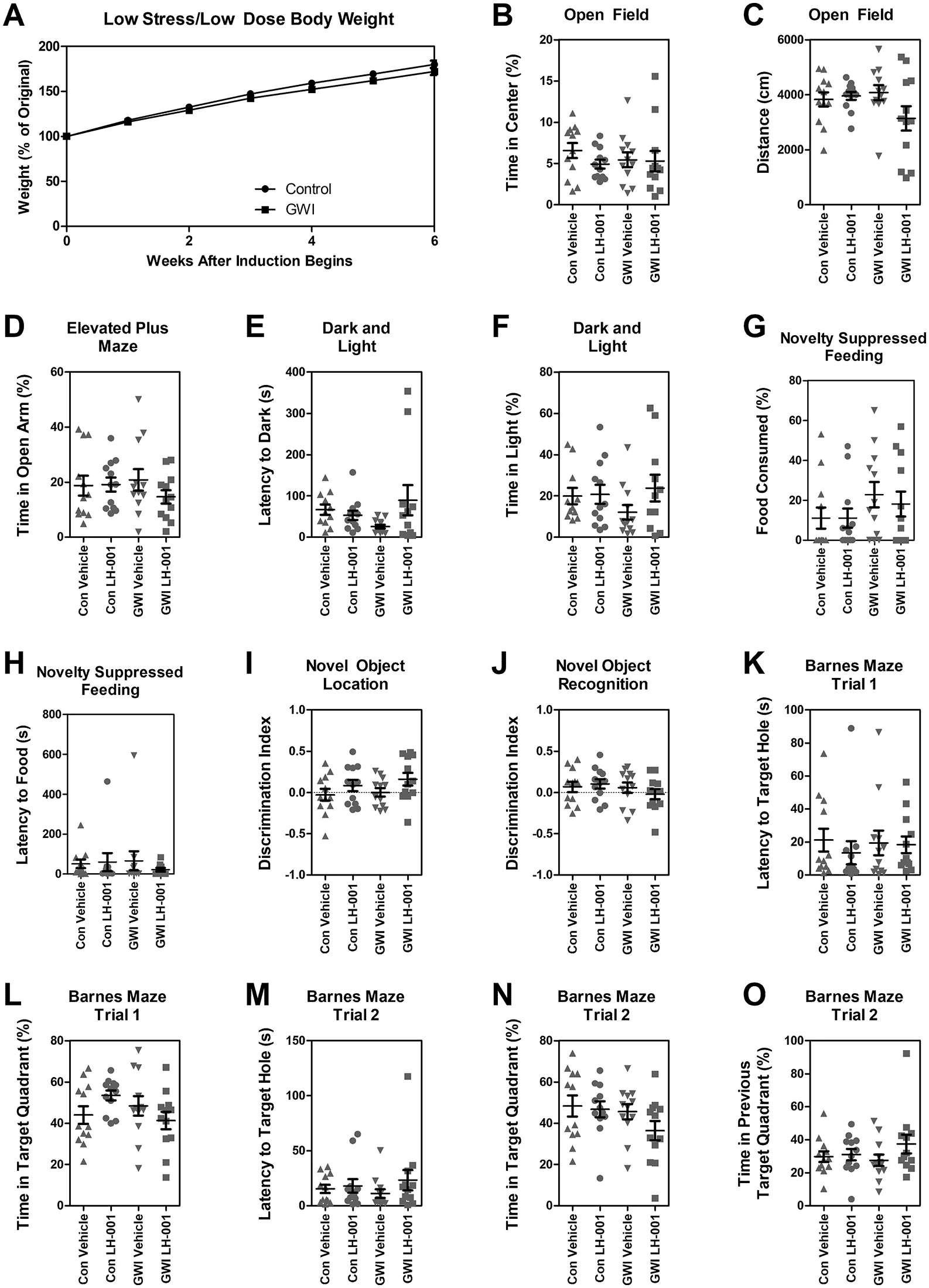
Low-dose/low-stress exposure does not alter body weight, anxiety-like behavior, or cognition in rats. Rats were exposed to low-dose chemical agents under a low-stress protocol for five weeks. Body weight was monitored during and after exposure. At 4–5 weeks post-exposure, rats received LH-001 or vehicle. Anxiety-like and cognitive behaviors were assessed beginning 8–9 weeks post-exposure. Data were analyzed using two-way ANOVA unless otherwise indicated. Unless specified, n = 12 per group. **(A) Body weight** measured weekly during the first six weeks (n = 24/group). No significant differences were observed between groups. **(B, C) Open-field test**: time spent in the center (B) and total distance traveled (C). **(D) Elevated plus maze**: percentage of time spent in the open arms. **(E, F) Light–dark transition test**: latency to enter the dark compartment (E) and percentage of time spent in the light compartment (F) (n = 11–12/group). **(G, H) Novelty-suppressed feeding test**: food consumption (G) and latency to feed (H) (n = 10–12/group). **(I, J) Recognition memory tests**: discrimination index for novel object location (I) and novel object recognition (J). **(K, L) Barnes maze training**: latency to find the escape hole (K) and time spent in the target quadrant (L). **(M, N) Barnes maze re-training**: latency to locate the new escape hole (M) and time spent in the new target quadrant (N) one week later. **(O) Barnes maze memory recall**: time spent in the original target quadrant during the re-training probe. No significant differences were observed between groups in any behavioral measure.

Procedures and behavioral assessments were otherwise kept consistent across studies to facilitate comparison, with exposure conditions representing the primary experimental variable. In the high-dose/low-stress study, three groups were included: control/vehicle, GWI/vehicle, and GWI/LH-001.

### Chemical agents

Chemical agents used for GWI induction in rats included the following: PB (Sigma-Aldrich Cat# P9797) dissolved in distilled water, PER (Sigma-Aldrich Cat# 45614) dissolved first in Dimethyl Sulfoxide (DMSO; Sigma-Aldrich Cat# 276855) and then diluted in 70% ethanol (diluted from 200 proof ethanol, Fisher scientific Cat# BP28184), and DEET (Sigma-Aldrich Cat# D100951) diluted in 70% ethanol. Batches of chemical agents were made fresh weekly and stored at 4°C when not in use. PER solution was sonicated for at least five minutes before use to minimize precipitation. All vehicles and chemicals were warmed to room temperature before use.

### GWI induction protocols

The induction protocols are summarized in Table 1. Rats were weighed at the start of each week. The average body weight within groups was determined to be used for preparing the chemical agents. Rats were treated with chemical agents or vehicle, followed by stress or no-stress every weekday for 4–5 weeks. Prior to chemical administration, animals were individually transferred from the housing room to the procedure room and maintained under ambient environmental conditions throughout the chemical and stress exposure procedure.

**Table 1:** GWI induction protocols.

Protocol	Chemical and stress exposure (every weekday for 4 or 5 weeks)				Vehicle or LH-001 (time to receive post-exposure)	Behavior testing (time to conduct post-exposure)
	PB (mg/kg)	PER (mg/kg)	DEET (mg/kg)	Stress		
**Low dose/** **low stress**	1.3	0.13	40	5-minute restraint stress (5 weeks)	4-5 weeks	8-9 weeks
**High dose/** **low stress**	13	2.6	215	5-minute restraint stress (5 weeks)	4-5 weeks	8-9 weeks
**Low dose/** **high stress**	1.3	0.13	40	2 unpredictable stressors (4 weeks)	4 weeks	10 weeks

PB: pyridostigmine bromide; PER: permethrin; DEET: N,N-Diethyl-meta-toluamide.

GWI group rats were administered PB via oral gavage at either a low (1.3 mg/kg) or high (13 mg/kg) dose in a 500 µl volume. This was followed by topical application to a shaved region of the neck with either a low (0.13 mg/kg) or high (2.6 mg/kg) dose of permethrin (PER; 200 µl), and either a low (40 mg/kg) or high (215 mg/kg) dose of DEET (200 µl). Following dermal application, animals were returned to their home cages and left undisturbed for approximately 5 minutes under standard ambient conditions to allow for topical absorption. Although the procedure was performed consistently according to this protocol, the exact interval for each individual animal was not prospectively recorded. No additional airflow or environmental manipulation was applied during this post-exposure period. Following the chemical absorption period, GWI rats were subjected to either 5 minutes of restraint stress using plastic DecapiCones (FisherSci, Cat. # NC9745006) or two different unpredictable stressors (Table 2). Control rats were treated via oral gavage with 500 µl of distilled water, followed by two topical applications of 70% ethanol (200 µl each, 400 µl total) on a shaved region of the neck. Control rats were not subjected to stress. Following the stress procedure, animals were returned to their home cages under standard vivarium conditions.

**Table 2:** Unpredictable stressors.

Stressor	Description of stressor
**Heat stress**	Rats were placed in cages warmed to 37^o^C via a heat lamp and left there for 1 hour. Cage temperatures were monitored with a thermometer. Rats were checked every 15 minutes.
**Cold stress**	Rats were placed in barren cages sitting on ice (temperature ranges between 8-14^o^C) for 1 hour. Rats were checked every 15 minutes.
**Food deprivation**	Food access was removed from home cage for 24 hours.
**Water deprivation**	Water access was removed from home cage for 24 hours.
**Single housing**	Rats were housed in novel cages, alone, and without enrichment for 24 hours.
**Light cycle disruption**	Rats in home cages were illuminated with a lamp for the duration of one full 12-hour dark cycle.
**Wet cage at a 30**^**o**^ **tilt**	Novel, barren cages containing a layer of water (the greatest depth is ~ 0.5 cm) were secured at 30^o^ tilt. Rats were placed in these cages for 4 hours.
**Restraint stress**	Rats were placed in well-ventilated clear plastic tubes measuring 2.5 cm in diameter and 25.5 cm in length with caps on either end. Rear caps contained a 1 cm x 1 cm hole for tail accessibility. Rats were restrained for 1 hour.
**Forced swim**	Rats were placed in a 6-gallon cylindrical fish tank with ~30 cm of 23-25^o^C water. Rats were left to swim for 10 minutes and monitored for the duration of the stressor and several minutes after. Rats were dried and returned to their home cage.
**Stroboscopic lighting**	Rats were subjected to randomized stroboscopic lighting in a dark room for 1 hour. Rats were checked every 15 minutes.
**Wet cage**	Rats were placed in novel, barren cages that had a layer of water ~3 cm deep for 3 hours. Rats were checked every 1 hour.

Following the 4–5 week exposure period, all rats remained undisturbed in their home cages for a 4–5 week washout period, during which they were handled only once weekly for body weight measurements and routine cage changes. Animals were then treated with either vehicle (saline) or the therapeutic compound LH-001. Behavioral testing commenced 4–6 weeks later (corresponding to 8–10 weeks post-induction). LH-001 or vehicle treatment was maintained continuously until euthanasia.

### Compound and treatment

LH-001, which was previously named LDN/OSU-0215111-M3 [24] and is a metabolite of LDN/OSU-0215111 [20], was used in the current study. It is an advanced compound being developed for clinical use. LH-001 (>95% purity) was highly soluble in water. After the resting period, rats were treated with either saline vehicle or LH-001 (10 mg/kg) every other day via oral gavage for 4 weeks. All animals continued to receive treatment in this manner until euthanasia.

### Behavior assessments

Rats were subjected to a series of behavior tests to assess anxiety and cognitive functions. All behavior testing was performed between 10 a.m. and 6 p.m. Prior to each experiment, animals were habituated to the testing room ambient conditions for at least 30 minutes. The rats were then single caged for 10 minutes immediately before behavioral testing. After testing, each animal was returned to its home cage. The apparatus was cleaned with 70% ethanol between each animal to disrupt scent cues, and all apparatuses were cleaned with 10% bleach at the end of each day. Data were captured and analyzed using SMART software (Version 3.0.05, Panlab Harvard Apparatus®). All behavioral tests were based on our previous study, with modifications [20]. The details of these modifications are described in each test section below.

#### Open field.

Rats were placed in the center of a 100 cm x 100 cm x 40 cm open field apparatus. Open field trials ran for 10 minutes under low light (approximately 30 Lux) and were recorded. The percentage of time spent in the center and total distance traveled were measured and analyzed.

#### Dark and light.

Dark and light testing was done using a 100 cm x 100 cm x 40 cm open field apparatus. A divider and lid were placed to create a 100 cm x 70 cm light field and a 100 cm x 30 cm dark field within the apparatus. The divider contained a small hole for rats to pass between the two fields. The light field was set to approximately 350 Lux. Rats were placed in the light field, away from the entrance to the dark field, and allowed to explore for 10 minutes. The percentage of time in the light field and latency time to the dark field were measured and analyzed.

#### Elevated plus maze.

This apparatus consisted of a plus shaped platform measuring 122 cm x 122 cm and stood 1 m above the ground. Two arms were closed while two arms were open. General lighting was set with the open arms being brighter (approximately 80 Lux) than the closed arms (approximately 30 Lux). Rats were placed on the end of an open arm and allowed to explore for 10 minutes. The percentage of time spent in the open arms was measured and analyzed.

#### Novelty-suppressed feeding.

Novelty-suppressed feeding was done using Kellogg’s brand Froot Loops cereal. Rats were introduced to the cereal in their home cages two days prior to testing. Rats were food deprived overnight right before the test trial. These tests were done in a 100 cm x 100 cm x 40 cm open field under bright light conditions, approximately 80–90 Lux. A small dish of cereal was placed in the center of the field. Rats were allowed to freely explore the open field and interact with the cereal for 10 minutes. At the end of the 10 minutes, rats were returned to their home cages, and the dish of remaining food was weighed. The latency to first interaction with the cereal and the total percentage of cereal eaten were measured and analyzed.

#### Novel object location.

Rats were first habituated to the empty 100 cm x 100 cm x 40 cm arena for 10 minutes per day over two days. On day three, two identical objects were placed into the upper right and upper left regions of the arena. The rats were given 10 minutes to freely interact with the objects. On day four, the upper right object was moved to the lower right of the arena. Rats were again given 10 minutes to interact with the objects. Time spent with each object was measured. A discrimination index [(novel % time – familiar % time) / (novel % time + familiar % time)] was calculated to determine the relative interaction preference. Positive values indicate more interaction with the novel location object, negative values indicate more relative time spent interacting with the unmoved object, and zero represents equal interaction with both objects. Memory deficits are indicated by a decreased discrimination index relative to controls. To determine whether a rat was interacting with the objects, a 12 cm × 12 cm zone was defined around the novel object and its location. The rat’s head and upper body had to be within this target area and remain detectable by the camera and tracking software used for quantification.

#### Novel object recognition.

This test was an extension of the novel object location test. After the familiar object was moved on day four, the unmoved familiar object was replaced with a similar, novel object on day five. Animals were again allowed to freely explore the two objects in the arena for 10 minutes. Memory was then analyzed by generating a discrimination index, as described above, for the novel versus familiar object, with cognitive deficits defined as discrimination index scores significantly lower than controls.

#### Barnes maze.

The Barnes Maze apparatus was a 48-inch diameter round table (~1 m above the ground) with twenty 8 cm diameter holes equidistantly cut into the perimeter. One of these holes was chosen to have an escape box affixed below it that the animals were trained to find to escape the bright, open arena. Eight visual cues were placed around the table. Lighting was set to 80–90 Lux to induce stress. During the training phase, rats were placed in the center of the Barnes maze apparatus under an opaque cylinder. Once the cylinder was removed, rats were left to freely explore the maze for up to 5 minutes or until the target hole was found and entered the escape box. The rats were left in the escape box for 1 minute before being returned to the home cage. In the event the rat did not find the escape hole by the 5-minute mark, it was guided to the hole with a clear beaker and allowed to stay in the beaker, over the hole, for up to 2 minutes. If the rat still did not find the hole in the allotted time, it was gently nudged with the beaker for up to 1 minute. If the rat still did not enter the hole, it was placed in the hole. Once in the escape hole, the rat was left to rest in the escape box for 1 minute. After this minute was up, the rat was returned to the home cage, and the table was cleaned with 70% ethanol. The table was rotated a quarter turn clockwise in preparation for testing the next rat. This was repeated once daily for 4 days (Day 1–4). Probe trials were conducted on Day 8 (3 days after the last training trial). During the probe trial, the escape box was removed, and animals freely explored the maze for 2 minutes. The latency to find the target hole and the time spent in the quadrant containing the target hole were measured and analyzed. After the completion of Probe trial 1, the rats were trained to a new target hole with the same procedure, from Day 9–12. Probe trial 2 was conducted on Day 16, and the data were analyzed the same as Probe trial 1.

### Immunohistochemical analysis

Immunohistochemistry was performed as previously described [20]. Briefly, rats from low-dose/high-stress study were anesthetized with a mixture of Xylazine and Ketamine and then perfused with ice cold phosphate buffered saline (PBS; 137 mM NaCl (Fisher Scientific Cat# S271), 2.7 mM potassium chloride (KCl; Fisher Scientific Cat# P217), 10 mM sodium phosphate dibasic (HNa2PO_4_; Fisher Scientific Cat# S393), 1.8 mM monobasic potassium phosphate (KH_2_PO_4_; Fisher Scientific Cat# BP362); pH 7.4) followed by ice cold 4% paraformaldehyde solution (PFA; Fisher Scientific Cat# T353). Brains were dissected out and left to post-fixing in 4% PFA at 4°C for at least one week. After fixing, brains were moved to a cryoprotectant solution consisting of 30% sucrose (PR1MA Cat# KCS24060) and 0.02% sodium azide (NaN_3_; Fisher Scientific Cat# S227) in PBS and left at 4°C for at least three days, or until the brains sunk to the bottom of the solution. Using a sliding microtome (Leica, SM2010R), the coronal sections were sliced at a 30 μm thickness and collected serially (every 10^th^ section) from the start of the hippocampus and going until no more hippocampal sections could be obtained. Sections were stored in PBS with 0.02% NaN_3_ at 4°C. Sections were blocked in 5% normal goat serum (Vector Laboratories Cat# S-1000–20) with 0.2% Triton-X100 (Sigma-Aldrich Cat# 9002-93-1) in PBS. Primary antibodies used for immunohistochemical staining included the following: glial fibrillary acidic protein (GFAP; 1:1000; Sigma-Aldrich Cat# G3893, RRID: AB_477010), parvalbumin (PV; 1:1000; Sigma-Aldrich Cat# P3088, RRID: AB_477329) and doublecortin (DCX; 1:500; Abcam Cat# ab18723, RRID: AB_732011). Secondary antibodies were purchased from Fisher Scientific and included Alexa Fluor 594 goat anti-mouse (1:200; Invitrogen Cat# A-11032, RRID: AB_2534091), anti-rabbit IgG (1:200; Invitrogen Cat# A-11037, RRID: AB_2534095), and DAPI (1:5000; Thermo Fisher Scientific Cat# D1306, RRID: AB_2629482). Images were taken with a fluorescent Axioskop 2 plus microscope (×10/0.25NA Achroplan, Carl Zeiss) using AxioVision (v4.8) software and analyzed with FIJI software [20]. Whole images of DG and HC were stitched together with a stitching plugin found within the FIJI software [25].

PV^+^ cells were counted manually and divided by the area of the entire hippocampus region. Data were averaged across all sections stained for all animals in that group (>5 sections per animal, n = 4 animals per group). DCX^+^ cells were counted manually after a brief digital processing on FIJI software. Briefly, images of whole hippocampi were outlined and cropped. Under the analyze tab the scale was set in accordance with our device’s appropriate scale setting (1.526 pixels = 1.00 μm). The areas of the hippocampi were found using the measure tool. After this, the image type was changed to 8-bit, the background was subtracted with a rolling ball radius of 30 and image threshold was adjusted to 0.18%. Images were then analyzed via the “analyze particles” function with the size (pixel^2) setting set to 35-Infinity. Cells found outside of the hippocampus were removed from the final count. Data were averaged across all sections stained for all animals in that group (≥4 sections per animal, n = 4 animals per group).

GFAP immunoreactivity was quantified in the dentate gyrus (DG), CA1, and CA3 regions of the hippocampus using FIJI (ImageJ) for image processing and automated particle analysis. Images were processed through channel separation, background subtraction, thresholding, and particle quantification to estimate GFAP-positive immunoreactive structures within predefined regions of interest.

For DG analysis, images were converted to 8-bit after scale calibration, background subtraction was performed (rolling ball radius = 30), and thresholding was applied using MaxEntropy with white objects on a black background. Three non-overlapping 200 × 200 pixel regions containing GFAP immunoreactivity were analyzed. Particle quantification was performed using the Analyze Particles function (size = 35–Infinity pixels; exclude on edges enabled).

For CA1 analysis, after scale calibration, the region was cropped to a 1200 × 500 pixel rectangle. Background subtraction was performed using a rolling ball radius of 30, and thresholding was applied using RenyiEntropy with “White Objects on Black Background” enabled. Particle quantification was performed using Analyze Particles (size = 5–Infinity pixels; Exclude on Edges enabled).

CA3 analysis followed the same procedure as CA1. The region was cropped to a 500 × 900 pixel rectangle, background subtraction was performed, thresholding was applied using RenyiEntropy, and particle quantification was performed using the same Analyze Particles settings as for CA1.

### Western blot analysis

Hippocampal tissues were placed in 10x volume of homogenization buffer and homogenized in 20 consecutive strokes using a motorized overhead stirrer (SouthwestScience Cat# SOS20) with a 2 ml glass mortar and polytetrafluoroethylene pestle. The homogenization buffer contained 0.32 M Sucrose (PR1MA Cat# KCS24060); 10 mM Tris (pH 7.5; Bio-Rad Cat# 161–0715); 1 mM EDTA (pH 8.0; Fisher Scientific Cat# BP120); 1 mM EGTA (VWR Cat# 0732); and 1x Protease Inhibitor (ThermoFisher Scientific Cat# A32965). Total cell lysate was sonicated (Fisher Scientific Cat# F60 Sonic Dismembrator) prior to reading the protein concentration using a DC™ Protein Assay Kit I (Bio-Rad Cat# 5000111). Samples were prepared and run on 8% SDS-PAGE polyacrylamide gels. Proteins were transferred from the gels onto nitrocellulose membranes using Bio-Rad’s Trans-Blot Turbo Transfer System (Bio-Rad Cat# 1704150). Membranes were blocked for 30 minutes in phosphate-buffered saline mixed with 0.1% Tween-20 (Fisher Scientific Cat# BP337–100) and 5% non-fat dried milk (Kroger, UPC: 0001111008736). Once blocked, membranes were probed overnight with primary antibody. The following antibodies were used: EAAT2 (1:20,000; custom antibody; [26]); VGLUT1 (1:500; EMD Millipore Corp. Cat# MAB5502, RRID:AB_262185); PSD95 (1:1000–2000; Invitrogen Cat# MA1–046, RRID: AB_2092361); actin (1:10,000; EMD Millipore Corp. Cat# MAB1501, RRID:AB_2223041); synaptophysin (1:500; Cell Signaling Technology Cat# 9020S); GFAP (1:1000; Millipore Sigma Cat# G3893, RRID:AB_477010); NCAM (1:1000; Cell Signaling Technology Cat# 99746S); gephyrin (1:500; GeneTex Cat# GTX109734, RRID:AB_11164321); VGAT (1:2000; Invitrogen Cat# PA5–96231, RRID:AB_2808033); and doublecortin (1:500; Abcam Cat# ab18723, RRID:AB_732011). Membranes were subsequently washed with phosphate buffered saline with 0.1% Tween-20 (PBST) and incubated in their respective secondary antibodies for one hour. Membranes were once again washed in PBST before probing with WesternBright ECL (Thomas Sci Cat# C942A85) or WesternBright Sirius (Thomas Sci Cat# C942A92) according to manufacturer’s instructions. Blots were imaged on the ChemiDoc™ Touch Imaging System (Bio-Rad Cat# 1708370). Band intensities were quantified with Image Lab software (Bio-Rad, V 5.2.1) then normalized to loading controls (GFAP or actin). Results are presented as fold-change relative to controls.

### Statistical analysis

We used GraphPad Prism (v5.01) to perform all data analysis and make all graphs. All n-values are shown in the figure legends. All body weight data were analyzed using a two-way repeated-measures ANOVA with GW exposure (GW-exposed vs. control) as the between-subjects factor and time as the within-subjects factor, followed by Bonferroni post hoc tests where appropriate.

Behavioral data from the low-dose/low-stress and low-dose/high-stress studies were analyzed using a two-way ANOVA with GW exposure (control vs. GWI) and LH-001 treatment (vehicle vs. LH-001) as independent factors, followed by Bonferroni-adjusted post hoc comparisons where appropriate. This analysis allowed assessment of the main effects of GW exposure and LH-001 treatment, as well as their interaction. If no significant main effects or interactions involving treatment were detected, data were subsequently collapsed across treatment groups to increase statistical power for testing the GW main effect. Comparisons between the combined “Control” and “GWI” groups were performed using an unpaired two-tailed t-test.

Behavioral data from the high-dose/low-stress study were analyzed using a one-way ANOVA, as this experiment included three groups (control/vehicle, GWI/vehicle, and GWI/LH-001). When the ANOVA was significant and assumptions of homogeneity of variance were met (assessed using Bartlett’s test), Tukey’s post hoc test was performed. If unequal variances were detected, a Kruskal–Wallis test followed by Dunn’s post hoc test was used.

Data from predefined non-responders were excluded from behavioral datasets. In the low-dose/low-stress study, one control/vehicle rat, two control/LH-001 rats, one GWI/vehicle rat, and one GWI/LH-001 rat were classified as non-responders in the novel suppressed feeding test because they remained immobile at the edge of the testing box for the entire duration of the test. In the low-dose/high-stress study, one GWI/vehicle rat and one GWI/LH-001 rat were classified as non-responders in the novel object location test because they remained immobile at the edge of the testing box throughout the test. We examined the influence of these exclusions on the overall conclusions and found that the overall interpretation of the behavioral findings was unchanged.

Immunohistochemical data were analyzed using two-way ANOVA, as described above. For these analyses, we used hippocampal sections from: 4 animals for DCX immunostaining (≥ 5 sections per animal); 4 animals for PV (≥ 4 sections per animal); and 3 animals for GFAP (≥ 3 sections per animal). Western blot data were analyzed by converting the signal to fold-change relative to the control vehicle. A one-sample t-test was used to compare these values to the theoretical value of 1.0. All data are reported as mean ± standard deviation (SD) with p-values less than 0.05 considered significant.

## Results

### Low-dose/low-stress exposure study

As described above in the Experimental Design, we originally planned to conduct four experimental cohorts following the protocol described by Parihar et al. [21]. Fig 1 shows the results from Cohort 1 (12 male rats per group; estimated power = 0.77 to detect a main effect with Cohen’s f = 0.4 at α = 0.05). All behavioral raw data and statistical data (including test statistics, degrees of freedom, significance level, effect size, confidence interval, and descriptive statistics) can be found in S1 and S4 File, respectively.

Body weight data are shown in Fig 1A. Two-way repeated-measures ANOVA of body weight indicated no significant differences between GW-exposed and non-GW-exposed rats at any measured time point (Fig 1A).

Anxiety-like behavioral data are shown in Fig 1B–1H. GW-exposed rats did not exhibit anxiety-like behavior in the open-field test, showing no reduction in time spent in the center (Fig 1B) or total distance traveled (Fig 1C). Consistent results were observed in the elevated plus maze, where GW-exposed and control rats spent comparable time in the open arms (Fig 1D). Similarly, in the light–dark transition test, GW-exposed rats showed no decrease in latency to enter the dark compartment (Fig 1E) or time spent in the light compartment (Fig 1F). In the novelty-suppressed feeding test, GW-exposed rats did not differ from controls in food consumption (Fig 1G) or latency to feed (Fig 1H). Two-way ANOVA revealed no significant main effect of GW exposure across all anxiety-related behavioral tests.

Cognitive assessment data are shown in Fig 1I–1O. GW-exposed rats showed no deficits in recognition memory, discriminating effectively between objects in novel and familiar locations (Fig 1I) and between novel and familiar objects (Fig 1J) 24 h after initial exposure. In the Barnes maze, GW-exposed rats also exhibited normal spatial learning and memory, with no increase in latency to locate the target hole (Fig 1K) or decrease in time spent in the target quadrant during the probe test conducted one week after training (Fig 1L). GW-exposed rats also required similar time as controls to locate a newly trained target hole (Fig 1M) and spent comparable time exploring the new (Fig 1N) and previous target quadrants (Fig 1O).

Two-way ANOVA revealed a significant main effect of LH-001 treatment in the novel object location test (F(1,44) = 4.088, p = 0.0493, ηp^2^ = 0.085; Fig 1I), suggesting that LH-001 treatment may enhance recognition of changes in object location regardless of GW exposure. However, post hoc Bonferroni tests did not reveal significant pairwise differences between groups. Two-way ANOVA also indicated a significant interaction between GW exposure and LH-001 treatment for time spent in the target quadrant in the Barnes maze (F(1,44) = 4.279, p = 0.0445, ηp^2^ = 0.089; Fig 1L), although Bonferroni post hoc comparisons were not significant. Control/LH-001 and GWI/vehicle groups showed a trend toward better spatial memory retention, whereas the GWI/LH-001 group showed performance similar to the control/vehicle group. Overall, these results indicate that low-dose/low-stress exposure did not produce detectable cognitive deficits, and LH-001 treatment showed a modest trend toward improving recognition or spatial memory.

Above behavioral outcomes were analyzed using two-way ANOVA with GW exposure and LH-001 treatment as independent factors. Because neither a significant main effect of LH-001 treatment nor a significant interaction between GW exposure and LH-001 treatment was detected, pooled analyses combining the control/vehicle and control/LH-001 groups as “Control” and the GWI/vehicle and GWI/LH-001 groups as “GWI” were performed as exploratory analyses to facilitate visualization of overall GW exposure effects. These pooled analyses were not used for primary statistical inference. Consistent with the factorial analyses, pooled comparisons did not detect statistically significant differences between the Control and GWI groups across behavioral tests (S5 File; p > 0.05). The pooled sample size (n = 24 per group) provided high power to detect large effect sizes (estimated power = 0.94 for Cohen’s d = 0.8 at α = 0.05), but smaller effects cannot be excluded.

#### High-dose/low-stress exposure study.

In the absence of a clear phenotype in the initial GWI model, it is difficult to evaluate whether LH-001 has the potential to provide therapeutic efficacy for GWI. Therefore, we increased the doses of PB (13 mg/kg), PER (2.6 mg/kg), and DEET (215 mg/kg) [22,23] while maintaining the same five-minute restraint stress protocol (the “high-dose/low-stress exposure” protocol; Table 1). In this experiment, three groups were included: control/vehicle, GWI/vehicle, and GWI/LH-001, with the aim of determining whether higher chemical doses could induce measurable disease phenotypes. The same procedures and behavioral assessments described above were performed. Fig 2 shows the results from experiment (8–15 male rats per group); estimated power = 0.62 to detect an effect with Cohen’s f = 0.4 at α = 0.05). All behavioral raw data and a statistical overview of the data can be found in S1 and S4 Files respectively.

**Fig 2:**
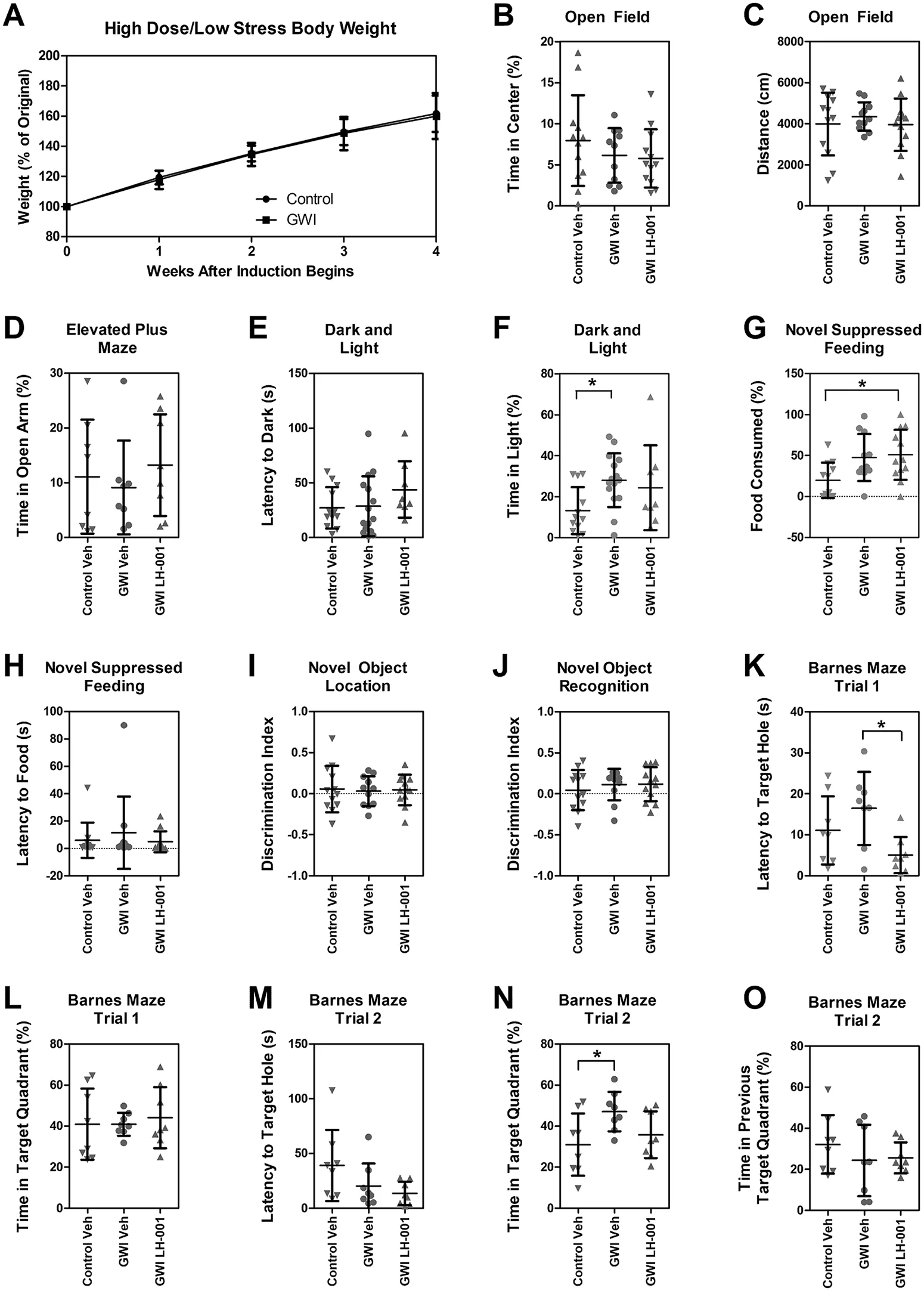
High-dose/low-stress exposure does not alter body weight or cognition but appears to reduce anxiety in rats. Rats were exposed to a high dose of chemical agents and a low-stress protocol for five weeks. Body weight was monitored during GW exposure. At 4−5 weeks post-exposure, rats received either LH-001 or vehicle. Anxiety-like and cognitive behaviors were assessed starting from 8–9 weeks post-exposure. A two-way ANOVA was used for body weight analysis. A one-way ANOVA was used for behavioral analysis. **(A) Body weight.** Body weights were monitored weekly over the first four weeks (n = 8 for control and n = 16 for GWI). No significant differences were found between groups. **(B, C) Open field test.** Time spent in the center (B) and total distance traveled (C) were measured (n = 12/group). No significant differences were found between groups. **(D) Elevated plus maze test.** The percentage of time spent in the open arms was analyzed (n = 8/group). No significant differences were found between groups. **(E-F) Dark and light test.** Latency to enter the dark chamber (E) and percent of time spent in the light chamber (F) were assessed (n = 8–15/group). GWI/vehicle rats spent significantly more time in the light area; GWI/LH-001 rats also spent more time in the light area but did not reach significance. **(G-H) Novelty-suppressed feeding test.** Amount of food consumed (G) and latency to begin feeding (H) were analyzed (n = 11–12/group). GWI/LH-001 rats ate significantly more food than control/vehicle rats; GWI/vehicle rats also ate more food but did not reach significance. Overall, high-dose/low-stress exposure appeared to reduce anxiety. **(I, J) Recognition memory test.** Discrimination index scores for novel object location (I) and novel object recognition (J) were calculated (n = 8/group). No significant differences were found between groups. **(K-L) Barnes maze test.** Latency to find the escape hole (K) and time spent in the target quadrant (L) during the initial training phase were analyzed (n = 8/group). GWI/LH-001 rats spent less time finding the target hole than GWI/vehicle rats. **(M-N) Barnes maze re-training.** Latency to the new escape hole (M) and time spent in the new target quadrant (N) were analyzed one week later (n = 8/group). No significant differences were found between groups. **(O) Barnes maze memory recall.** Time spent in the original target quadrant during the re-training phase was analyzed (n = 8/group). GWI/rats spent significantly more time in the target quadrant than control/vehicle rats. Overall, high-dose/low-stress exposure did not cause cognitive issues. *p < 0.05.

Two-way repeated-measures ANOVA of body weight data indicated no significant differences between GW-exposed and control rats at any measured time point (Fig 2A).

For behavioral data, Bartlett’s test revealed unequal variances for the following behavioral measures: novelty-suppressed feeding amount of food consumed (Fig 2G), Barnes maze trial 1 target quadrant time (Fig 2L), and trial 2 latency (Fig 2M). These data were analyzed using the Kruskal–Wallis test followed by Dunn’s post hoc test, while the remaining data were analyzed using one-way ANOVA with Tukey’s post hoc tests.

Anxiety-related behavioral data are shown in Fig 2B–2H, with detailed statistical results provided in Supplementary S4 File. Increased chemical doses did not induce anxiety-like phenotypes in the open-field test (Fig 2B–2C), elevated plus maze (Fig 2D), light–dark transition test (Fig 2E–2F), or novelty-suppressed feeding test (Fig 2G–2H). Interestingly, GWI groups tended to exhibit reduced anxiety-like behavior compared with controls. Specifically, GWI/vehicle rats spent significantly more time in the light compartment in the light–dark transition test (F(2,32) = 3.553, p = 0.0404, η² = 0.182; Fig 2F), while GWI/LH-001 rats consumed significantly more food in the novelty-suppressed feeding test (F(2,32) = 4.697, p = 0.0163, η² = 0.227; Fig 2G). In each case, the other GWI group (GWI/LH-001 in the light–dark test and GWI/vehicle in the novelty-suppressed feeding test) showed a similar trend toward reduced anxiety-like behavior, although these differences did not reach statistical significance.

Cognitive assessments (Fig 2I–2O) revealed no deficits in novel object location (Fig 2I), novel object recognition (Fig 2J), or Barnes maze performance (Fig 2K–2O). However, GWI/LH-001-treated rats located the target hole significantly faster than control and GWI/vehicle rats during Barnes maze testing (F(2,21) = 4.639, p = 0.0214, η^2^ = 0.306; Fig 2K). Overall, increasing chemical doses did not produce anxiety-like phenotypes or cognitive deficits.

#### Low-dose/high-stress exposure study.

Several reports from the Committee on Gulf War and Health indicate that GW veterans experienced multiple war-related psychological stresses, such as deployment and combat stress, and environmental stresses, such as dust storms, intense heat during the day and cold at night, and poor living condition [2]. Stress may potentiate the effects of GW chemical agents in the brain and other systems/organs. Higher levels of stress may produce more obvious GWI-like phenotypes [27,28]. We therefore decided to conduct a study using the protocol of GW chemical agents combined with multiple unpredictable stressors (the “low-dose/high-stress exposure” protocol in Table 1), similar to the mouse model that we previously established [20]. We measured body weight and performed the same behavioral assessments as described above. Fig 3 shows the results from this study (12 male rats per group; estimated power = 0.77 to detect a main effect with Cohen’s f = 0.4 at α = 0.05). All behavioral raw data and a statistical overview of the data can be found in S1 and S4 Files respectively.

**Fig 3:**
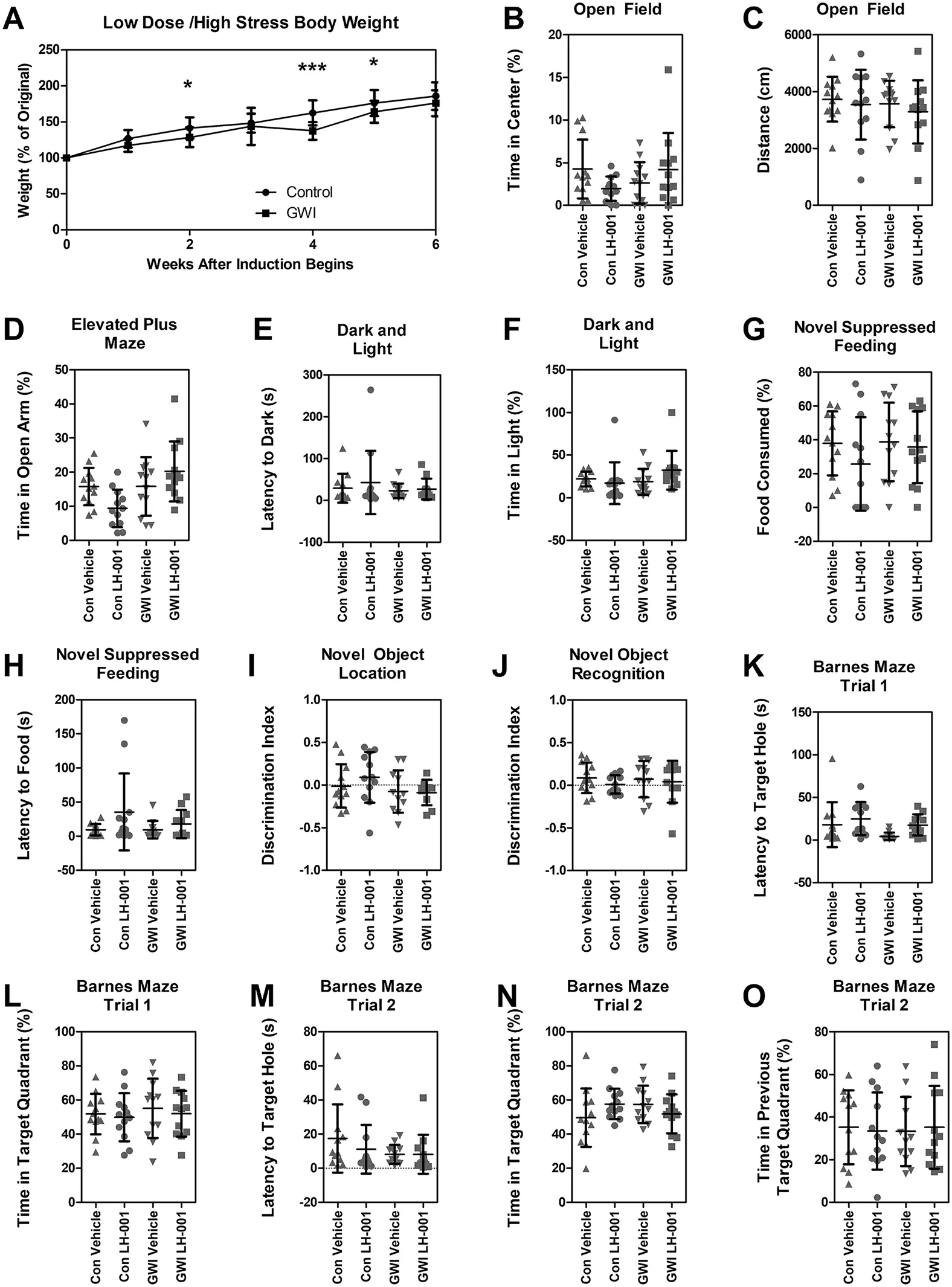
Low-dose/high-stress exposure reduces body weight but does not cause anxiety or cognitive impairment in rats. Rats were exposed to low doses of chemical agents and a variety of high stressors. Body weight was monitored during and post-GW exposure. At 4 weeks post-exposure, rats received either LH-001 or vehicle. Anxiety-like and cognitive behaviors were assessed starting from 10 weeks post-exposure. Data were analyzed using two-way ANOVA unless otherwise indicated. **(A) Body weight.** Body weights were monitored weekly over the first six weeks (n = 24/group). GW-exposed groups showed a significant reduction in body weight during the GW exposure period. **(B, C) Open field test.** Time spent in the center (B) and total distance traveled (C) were measured (n = 12/group). No significant differences were found between groups. **(D) Elevated plus maze test.** The percentage of time spent in the open arms was analyzed (n = 12/group). GWI/LH-001 rats spent more time in the open arm when compared to control/LH-001 rats. **(E-F) Dark and light test.** Latency to enter the dark chamber (E) and percent of time spent in the light chamber (F) were assessed (n = 11–12/group). No significant differences were found between groups. **(G-H) Novelty-suppressed feeding test.** Amount of food consumed (G) and latency to begin feeding (H) were analyzed (n = 11–12/group). No significant differences were found between groups. **(I, J) Recognition memory test.** Discrimination index scores for novel object location (I) and novel object recognition (J) were calculated (n = 11–12/group). No significant differences were found between groups. **(K-L) Barnes maze test.** Latency to find the escape hole (K) and time spent in the target quadrant (L) during the initial training phase were analyzed (n = 12/group). GWI/vehicle rats found the target hole more quickly when compared to control/LH-001 rats. **(M-N) Barnes maze re-training.** Latency to the new escape hole (M) and time spent in the new target quadrant (N) were analyzed one week later (n = 12/group). No significant differences were found between groups. **(O) Barnes maze memory recall.** Time spent in the original target quadrant during the re-training phase was analyzed (n = 12/group). Overall, no general trend for anxiety-like phenotypes or cognitive deficits with increasing stress was found. *p < 0.05, ***p < 0.001.

Two-way repeated-measures ANOVA of body weight showed a significant reduction in body weight in GW-exposed rats during the exposure period at weeks 2, 4, and 5 (Fig 3A), consistent with our previous findings in mice [20].

The anxiety-related behavioral data are shown in Fig 3B–3H. Two-way ANOVA revealed a significant interaction for time spent in the center in the open-field test (F(1,44) = 4.682, p = 0.0360, ηp^2^ = 0.096; Fig 3B), with the control/LH-001 and GWI/vehicle groups spending less time in the center. However, post hoc tests did not detect significant differences between groups. No significant differences were observed in total distance traveled in the open-field test (Fig 3C).

In the elevated plus maze, there was a main effect of GW exposure (F(1,44) = 6.625, p = 0.0135, ηp^2^ = 0.131) and a significant interaction between GW exposure and LH-001 treatment (F(1,44) = 6.542, p = 0.0141, ηp^2^ = 0.130) (Fig 3D). GW-exposed rats spent more time in the open arms, whereas control/LH-001 rats spent less time in the open arms; however, post hoc comparisons did not reveal significant group differences. No main effects or interactions were detected in the light–dark transition test (Fig 3E, 3F) or in the novelty-suppressed feeding test (Fig 3G, 3H).

The significant main effect of GW exposure and its interaction with LH-001 treatment in the elevated plus maze suggest that the effect of LH-001 may occur only in GW-exposed rats, but not in non-exposed rats. Several explanations are possible. First, low-dose/high-stress exposure may sensitize neural circuits such as the hypothalamic–pituitary–adrenal axis or specific neurotransmitter systems (e.g., glutamatergic signaling), and LH-001 may act to normalize these stress-sensitized pathways. Second, LH-001 may require a stress-induced signaling environment to exert anxiolytic effects. Third, LH-001 and the low-dose/high-stress exposure may act on the same biological system in opposite directions, such that the treatment effect becomes detectable only when stress elevates the system above a behavioral or neurochemical threshold.

Taken together, these data provide no clear evidence of anxiety-like phenotypes despite increased stress exposure. Notably, in our previous study, mice exposed to the same GW paradigm developed clear anxiety-like phenotypes three months post-exposure that persisted for at least one year [20].

The cognition data are shown in Fig 3I–3O. No significant effects were observed in the novel object location (Fig 3I) or novel object recognition tests (Fig 3J), indicating no changes in spatial or recognition memory. In the Barnes maze (Fig 3K–3O), two-way ANOVA revealed a main effect of GW exposure on latency to the target hole (F(1,44) = 4.353, p = 0.0428, ηp^2^ = 0.090; Fig 3K). GWI/vehicle rats located the target hole more quickly in the initial trial than the other groups; however, post hoc comparisons did not reveal significant differences between groups. The main effect suggests that rats exposed to the low-dose/high-stress protocol located the target hole faster than control rats. This unexpected result may have several explanations. First, low-dose/high-stress exposure may increase exploratory drive, causing GWI rats to search more actively and move faster than control rats. Second, exposure may alter spatial learning strategies in a way that benefits performance in this task, possibly by changing how rats prioritize spatial cues.

For tests in which two-way ANOVA showed no significant main effect of LH-001 treatment and no interaction between GW exposure and LH-001 treatment, the control/vehicle and control/LH-001 groups were combined as “Control” and the GWI/vehicle and GWI/LH-001 groups were combined as “GWI.” Analysis of the pooled data also showed no statistically significant differences between the “Control” and “GWI” groups across all tests (n = 24 per group; estimated power = 0.94 to detect Cohen’s d = 0.8 at α = 0.05) (S6 File; p > 0.05) Overall, these data provide no evidence of cognitive deficits in rats subjected to the low-dose/high-stress protocol.

#### Pathological studies.

After completing the behavioral assessments, the rats were euthanized for further pathological studies. We focused on the hippocampal regions of the high stress/low dose cohort rats. In our previous study in mice [20], by immunohistochemical staining, we did not observe significant changes for hippocampal pyramidal neurons and microglia regarding the number, morphology, and signal intensity between GW-exposed and control mice. However, we observed significant changes for the following cell types in GW-exposed mice: (i) astrocytes with a reduced area of glial fibrillary acidic protein-positive (GFAP^+^) elements, suggesting astrocytic atrophy; (ii) parvalbumin-positive (PV^+^) interneurons with a reduced number, suggesting their loss; and (iii) doublecortin-positive (DCX^+^) newborn neurons with a reduced number, indicating decreased neurogenesis. We therefore conducted immunohistochemical staining using GFAP, PV, and DCX antibodies to examine astrocytes, PV interneurons, and newborn neurons, respectively. Hippocampal sections from 3–4 animals, with at least 3 sections per animal, were analyzed for each staining. Two-way ANOVA was used for all immunohistochemistry analysis. Results show a significant main effect of the GW exposure for GFAP - CA1 (*F*(1,128) = 4.881, *p* = 0.0289, ηp^2^ = 0.0367; Fig 4B), GFAP - CA3 (*F*(1,131) = 4.883, *p* = 0.0289, ηp^2^ = 0.0367; Fig 4C), and DCX – DG (*F*(1,125) = 11.280, *p* = 0.0010, ηp^2^ = 0.0828; Fig 4E). However, Bonferroni post hoc tests showed no significant differences among the four groups in the GFAP immunoreactivity quantification in CA1 or CA3, or DCX⁺ newborn neurons in the DG. In addition, no significant differences were observed in the GFAP immunoreactivity quantification in the DG (Fig 4A) or PV⁺ interneurons in the hippocampus (Fig 4D). These results suggest that low-dose/high-stress exposure trends towards reduced neurogenesis in the DG and increased astrogliosis in the CA1 and CA3 regions of the hippocampus but did not reach statistical significance.

**Fig 4:**
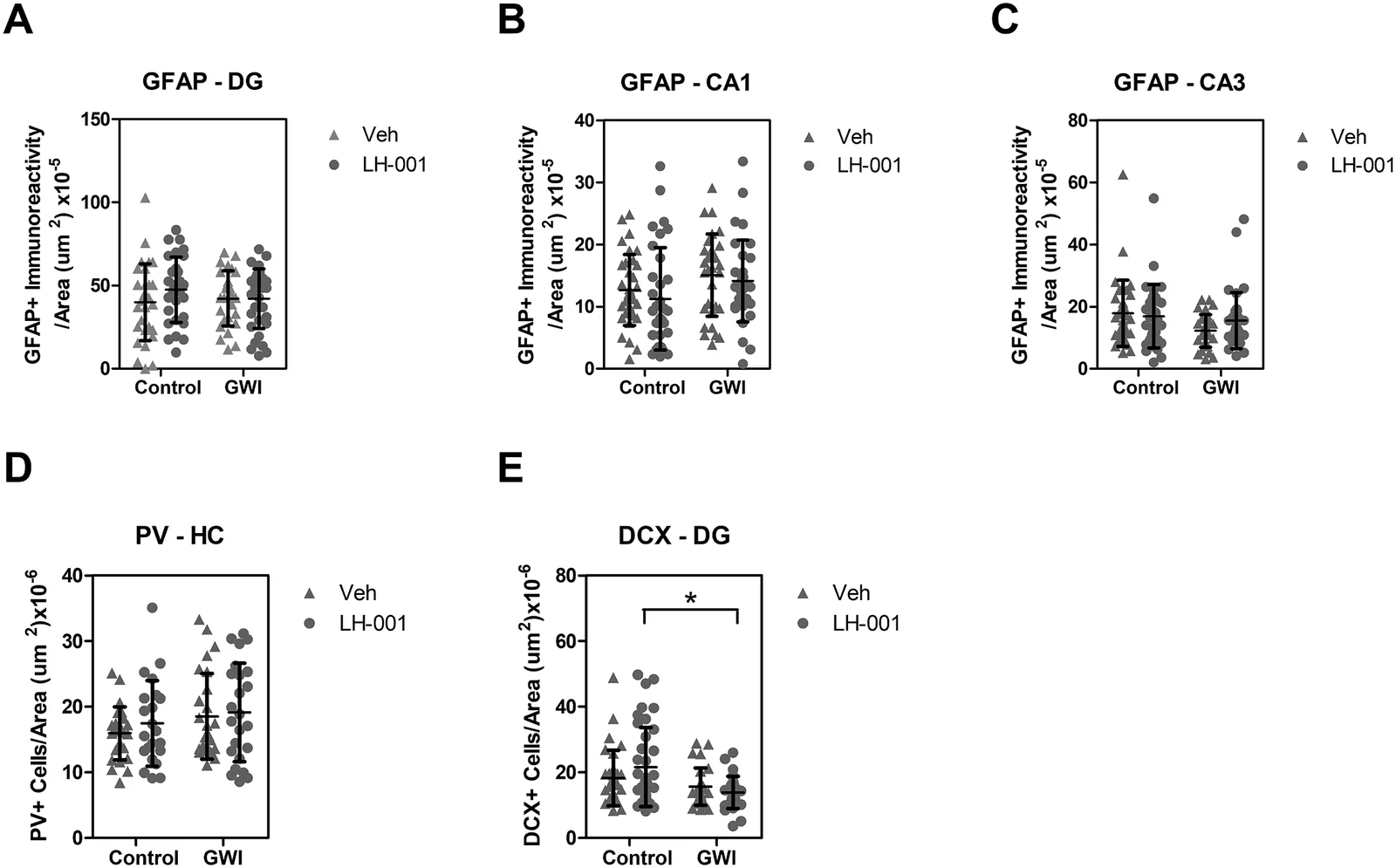
Low-dose/high-stress exposure trends towards reduced hippocampal neurogenesis and increased astrogliosis in the CA1 and CA3 regions. Following behavioral assessments (the above low-dose/high-stress exposure study), a subset of rats was euthanized to examine markers for astrocytes (GFAP^+^), parvalbumin-positive interneurons (PV^+^), and newborn neurons (DCX^+^) in hippocampal regions using immunohistochemical staining. For each staining, 3–4 animals and at least 3 sections per animal were analyzed. Data were analyzed by two-way ANOVA. **(A-C) the GFAP immunoreactivity quantification**. DG, n = 27–33; CA1, n = 31–34; CA3, n = 31–36. **(D) the number of PV**^**+**^ **neurons**. n = 22–26. **(E) the number of DCX**^**+**^ **newborn neurons**. n = 29–39. Two-way ANOVA revealed a significant main effect of the GW exposure for GFAP - CA1, CA3, and DCX – DG; however, Bonferroni post hoc tests showed no significant differences among the four groups.

We further examined total expression levels of several proteins in hippocampi by Western blot analysis. The proteins studied were: (1) synaptophysin (SYP) and vesicular glutamate transporter 1 (VGLUT1), which are expressed in the presynaptic terminals of glutamatergic neurons; (2) postsynaptic density protein 95 (PSD95) and neural cell adhesin molecule (NCAM), which are expressed in the postsynaptic terminals of glutamatergic neurons; (3) glutamate transporter EAAT2 and GFAP, which are primarily expressed in astrocytes; (4) vesicular GABA transporter (VGAT), which is expressed in the presynaptic terminals of GABAergic neurons; (5) gephyrin (GPHN), which is primarily expressed in the postsynaptic terminals of GABAergic neurons; and (6) doublecortin (DCX), which is expressed in newborn neurons. Results showed that total expression levels of synaptic proteins, including SYP (mean = 1.263, SD = 0.305) (t (11) = 2.862, p = 0.0155, Cohen’s d = 0.863), VGLUT1 (mean = 1.398, SD = 0.533) (t (11) = 2.4720, p = 0.0310, Cohen’s d = 0.745), PSD95 (mean = 1.313, SD = 0.454) (t (11) = 2.282, p = 0.0434, Cohen’s d = 0.688), NCAM (mean = 1.163, SD = 0.214) (t (11) = 2.534, p = 0.0278, Cohen’s d = 0.764), and VGAT (mean = 1.193, SD = 0.280) (t (11) = 2.291, p = 0.0427, Cohen’s d = 0.691, and astrocytic protein, EAAT2 (mean = 1.451, SD = 0.556) (t (11) = 2.688, p = 0.0211, Cohen’s d = 0.8105), were significantly increased in the control/LH-001 group compared to the control/vehicle group, which reflected LH-001’s effects (Fig 5). Notably, the GWI/vehicle group also showed significantly increased total expression levels of VGLUT1 (mean = 1.414, SD = 0.476) (t (11) = 2.885, p = 0.0148, Cohens d = 0.870) and EAAT2 (mean = 1.361, SD = 0.513) (t (11) = 2.335, p = 0.0395, Cohens d = 0.704) compared to the control/vehicle group. This suggests that GW exposure may increase glutamate release, and that the observed increase in EAAT2 expression may be a compensatory mechanism to maintain glutamate homeostasis and prevent excitotoxicity. Similarly, VGLUT1 (mean = 1.534, SD = 0.592) (t (11) = 2.992, p = 0.0122, Cohen’s d = 0.902) and EAAT2 (mean = 1.379, SD = 0.550) (t (11) = 2.228, p = 0.0429, Cohen’s d = 0.690) expression levels were also increased in the GWI/LH-001 group.

**Fig 5:**
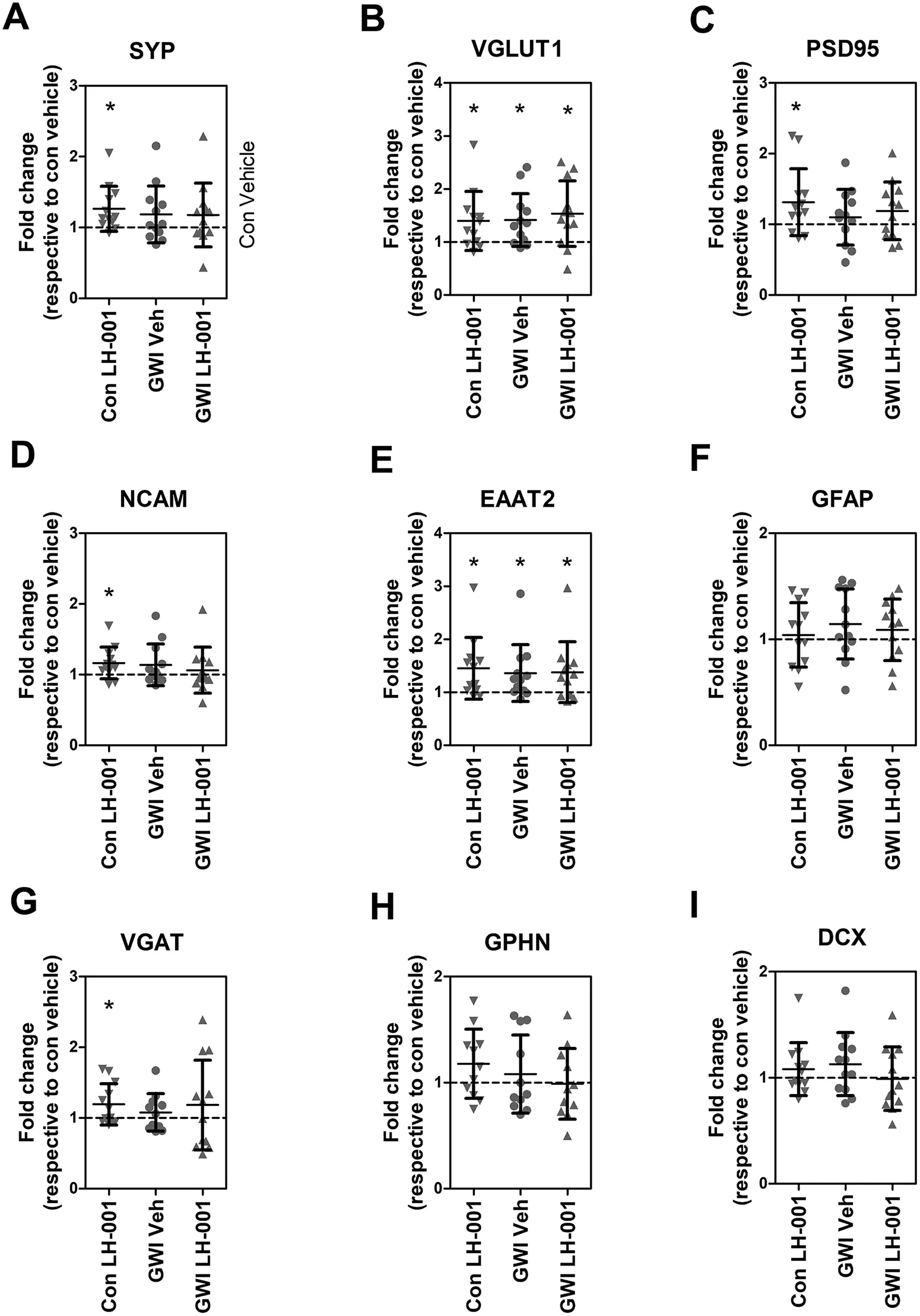
Effects of low-dose/high-stress exposure and LH-001 treatment on synaptic and astrocytic protein expression in the rat hippocampus. Total cell lysates were prepared from the hippocampus and analyzed for protein expression by Western blotting, followed by quantitative analysis. Results are presented as fold-change relative to the control/vehicle group, which is represented by a dashed line. Data was analyzed by unpaired t-test (n = 11−12 each group). The levels of glutamatergic synaptic proteins, including SYP, VGLUT1, PSD95, and NCAM, were significantly increased in the control/LH-001 group compared to the control/vehicle group. The GABAergic synaptic protein VGAT and the astrocytic protein EAAT2 were also significantly increased in the control/LH-001 group compared to the control/vehicle group. Additionally, the levels of VGLUT1 and EAAT2 were significantly increased in the GWI/vehicle and GWI/LH-001 groups compared to the control/vehicle group. Data represents the analysis of six animals per group. Statistical significance was determined using a one-sample t-test (*p < 0.05).

## Discussion

This study originally aimed to test the efficacy of compound LH-001, a novel synapse enhancer, in rat models of GWI. Unexpectedly, our initial GWI induction protocol in rats did not produce robust GWI-like behavioral phenotypes reported in previous studies [21,29]. After adjusting the induction protocol twice—once to a more potent chemical exposure and once to a higher level of stress we still did not detect robust GWI-like behavioral phenotypes under the experimental conditions used. These findings highlight the challenges of reproducing GWI-related phenotypes across experimental settings and underscore the importance of continued efforts to validate and standardize GWI animal models.

These unexpected results prompted a closer examination of published GWI induction protocols. A comparison of our study with previous studies reporting behavioral outcomes is summarized in Table 3. Several observations emerged from this comparison. First, considerable heterogeneity exists among published GWI protocols, including differences in chemical exposure regimens, stress paradigms, timing of behavioral assessments, animal sources, and other experimental conditions. Such variability may contribute to differences in behavioral outcomes observed across laboratories.

**Table 3:** GWI induction protocols.

Study	Chemical exposure	Stressor	Testing timing	Stain Sex Source	Conditions	Behavioral Outcomes	Ref
1a	PB (1.3 mg/kg) PER (0.13 mg/kg) DEET (40 mg/kg) every weekday for 5 weeks	5-min restraint every weekday for 5 weeks	Staring from 8–9 weeks post-exposure	Rat/SD male (CRL, Raleigh, NC, USA)	1. vehicle (no stress) 2. chemical + stress (n = 12/group)	• The chemical + stress group showed no evidence of anxiety-like phenotypes in the **OFT**, **LDT**, **EPMT**, or **NSFT**, nor did it show cognitive deficits in the **OLT**, **NORT**, or **BMT**, compared to the control group. • Individual rat data were shown for each behavioral test.	this study
1b	PB (13 mg/kg) PER (2.6 mg/kg) DEET (215 mg/kg) every weekday for 5 weeks	5-min restraint every weekday for 5 weeks	Starting from 8–9 weeks post-exposure	Rat/SD male (CRL, Raleigh, NC, USA)	1. vehicle (no stress) 2. chemical + stress (n = 8–15/group)	• The chemical + stress group showed no evidence of anxiety-like phenotypes in the **OFT**, **LDT**, **EPMT**, or **NSFT**, nor did it show cognitive deficits in the **OLT**, **NORT**, or **BMT**, compared to the control group. • Individual rat data were shown for each behavioral test.	this study
1c	PB (1.3 mg/kg) PER (0.13 mg/kg) DEET (40 mg/kg) every weekday for 4 weeks	2 unpredictable stressors every weekday for 4 weeks	Starting from 10 weeks post-exposure	Rat/SD male (CRL, Raleigh, NC, USA)	1. vehicle (no stress) 2. chemical + stress (n = 12/group)	• The chemical + stress group showed no evidence of anxiety-like phenotypes in the **OFT**, **LDT**, **EPMT**, or **NSFT**, nor did it show cognitive deficits in the **OLT**, **NORT**, or **BMT**, compared to the control group. • Individual rat data were shown for each behavioral test.	this study
2	PB (1.3 mg/kg) PER (0.13 mg/kg) DEET (40 mg/kg) daily for 4 weeks	5-min restraint daily for 4 weeks	Starting from 3 months post-exposure	Rat/SD, No gender info provided (Harlan, Indianapolis, IN, USA)	1. vehicle (no stress) 2. chemical (no stress) 3. chemical + stress (n = 6/group)	• The chemical (no stress) and chemical + stress groups showed (i) increased immobility time in **FST**, (ii) fewer open-arm entries and reduced open-arm dwell time in **EPMT**, (iii) impaired spatial learning in **MWM**, and (iv) normal recognition memory in **NORT**, compared to the control group. • No individual rat data was shown for each behavioral test.	[21]
3	PB (1.3 mg/kg) PER (0.13 mg/kg) DEET (40 mg/kg) daily for 4 weeks	5-min restraint daily for 4 weeks	Starting from 3 months post-exposure	Rat/SD, No gender info provided (Harlan, Indianapolis, IN, USA)	1. naive control (no exposure) 2. chemical + stress (n = 16–28/group)	• The chemical + stress group exhibited (i) no anxiety-like behavior in **OFT**, (ii) impaired location memory in **OLT**, (iii) impaired recognition memory in **NORT**, (iv) decreased motivation for running on wheel, and (v) longer latency to eat food in **NSFT**, compared to the control group. • No individual rat data was shown in each behavioral test.	[29]
4	PB (2 mg/kg) PER (0.2 mg/kg) DEET (60 mg/kg) daily for 4 weeks	15-min restraint daily for 4 weeks	Starting from 10 months post- exposure	Rat/SD male (Harlan, Indianapolis, IN, USA)	1. naive control (no exposure; n = 23 for **NORT**, n = 10 for **OIPT**) 2. chemical + stress (n = 19 for **NORT**, n = 16 for **OIPT**)	• The chemical + stress group exhibited (i) impaired recognition memory in **NORT** and (ii) impaired object-in-place associative recognition memory in **OIPT**. • No individual rat data was shown in each behavioral test.	[35]
5	PB (1.3 mg/kg) daily for 14 days	6-hour restraint daily for 10 days	Starting from 12 weeks post-exposure	Rat/SD male (Harlan, breeding site not provided, USA)	1. vehicle (no stress) 2. PB (no stress) 3. vehicle + stress 4. PB + stress (n = 12–15/group)	• No significant difference in the time spent exploring the novel object (**NORT**) was found between groups. • When challenged with acute LPS, PB-treated rats, irrespective of stress history, spent significantly less time with the novel object in **NORT** and exhibited reduced time in the target quadrant in **MWM**. • No individual rat data was shown in each behavioral test.	[36]
6	PB (1.3 mg/kg) daily and intranasal LPS (50 µl of 10 mg/5 ml) every weekday for 33 days	1 unpredictable stressor daily for 33 days	Starting on day 35 post-exposure	Rat/Wistar male & female (Harlan, Indianapolis, IN, USA)	1. naive control (n = 14; all males) 2. chemical + stress (n = 36; mixed males & females)	• The chemical + stress group rats spent significantly less time with the novel object in **NORT**, in the open arm in **EPMT**, and in the center of the open field in **OFT**, compared to the no exposure group. There was no significant difference in the time spent in TST and FST between the groups. • Individual rat data were shown in each behavioral test.	[30]
7	PB (1.3 mg/kg) PER (0.13 mg/kg) DEET (40 mg/kg) daily for 4 weeks	5-min restraint daily for 4 weeks	Starting from 3 months post-exposure	Rat/SD male (CRL, Wilmington, MA, USA)	1. vehicle (no stress) 2. chemical (no stress) 3. chemical + stress (n = 9–10/group)	• The chemical and chemical + stress groups showed no significant differences in (i) motor response to a sudden, intense sound in **ASR** or (ii) spatial learning and memory in **MWM**, but did show an increase in (iii) motor activity level in **OFT** when compared to the control group • No individual rat data was shown in each behavioral test.	[37]
8	PB (1.3 mg/kg) daily for 2 weeks	6-hour restraint daily for 10 days	10 days post-exposure	Rat/SD male (animal source not provided)	1. vehicle (no stress) 2. vehicle + stress 3. PB (no stress) 4. PB + stress (n = 13–14/group)	• The PB + stress and vehicle + stress groups exhibited deficits in ability to learn and remember an association between a tone and a foot shock in **CFC** test, but the PB alone group did not. • No individual rat data was shown in each behavioral test.	[28]
9	PB (1.5 mg/kg) every weekday for 2 weeks	**PCA** stress for 25 min every weekday for 2 weeks	Day 52, 113, & 199 post-exposure	Rat/Wistar male (Harlan, Gannat, France)	1. vehicle (no stress) 2. vehicle + stress 3. PB (no stress) 4. PB + stress (n = 18–20/group)	• The PB + stress group exhibited a learning deficit in **MWM**, which was not observed in the PB or stress group. The PB + stress group also showed significantly increased aggression in **SEA** on day 52 post-exposure, but not on day 113 or 199. • No individual rat data was shown in each behavioral test.	[38]
10	PB (2.0 mg/kg) PER (0.2 mg/kg) DEET (60 mg/kg) daily for 4 weeks	15-min restraint daily for 4 weeks	Starting from 8.5 months post-exposure	Rat/SD male (Harlan, Indianapolis, IN, USA)	1. naive control 2. chemical + stress (n = 10–15/group)	• The chemical + stress group exhibited (i) impaired location memory in **OLT**, (ii) impaired pattern separation memory in **PST**, (iii) anhedonia-like behavior in **SPT**, and (iv) hyperalgesia-like behavior in **VFT**, compared to the control group. • Individual rat data were shown in each behavioral test.	[31]
11	PB (1.3 mg/kg) PER (0.13 mg/kg) DEET (40 mg/kg) daily for 4 weeks	5-min restraint daily for 4 weeks	5 and 10 months post-exposure	Rat/SD male (Taconic Farms, Rockville, MD, USA)	naive control (n = 10) 2. chemical + stress (n = 20)	• The chemical + stress group exhibited (i) increased in aggressive behavior, (ii) increased anxiety-like behavior in **OFT** and **EPMT**, (iii) dysfunctional novel object recognition memory in **NORT**, (iv) dysfunctional spatial learning and memory in **MWM**, compared to the control group. • No individual rat data was shown in each behavioral test.	[39]
12	PB (1.3 mg/kg) PER (0.13 mg/kg) DEET (40 mg/kg) daily for 4 weeks	5-min restraint daily for 4 weeks	Starting from 1–4 days post exposure	Rat/SD male (animal source not provided)	1. control 2. chemical + stress 3. chemical + stress + minocycline (n = 18/group)	• The chemical + stress group exhibited (i) increased anxiety like behavior in the **OFT** and **EPMT** compared to control and minocycline groups, (ii) memory deficits compared to the other two groups in the **NORT**, and (iii) greater depressive-like behavior in the **FST** compared to the other two groups. • Individual data was presented in each behavioral test.	[32]

CRL: Charles River Laboratories; LPS: lipopolysaccharide; SD: Sprague-Dawley.

**ASR**: acoustic startle response; **BMT**: Barnes maze test; **CFC**: cued fear conditioning; **EPMT**: elevated plus maze test; **FST**: forced swim test; **LDT**: light and dark transition; **MWM**: Morris water maze; **NORT**: novel object recognition test; **NSFT**: novelty-suppressed feeding test; **OFT**: open field test; **OIPT**: object-in-place associative recognition memory test; **OLT**: object location test; **PCA**: pole climbing avoidance; **PST**: pattern separation test; **SEA**: shock-elicited aggression test; **SPT**: Sucrose preference test; **TST**: tail suspension test; **VFT**: Von Frey test

Second, reporting practices varied across published studies. Among the eleven published studies (Studies 2–12) summarized in Table 3, only Studies 6 [30], 10 [31], and 12 [32] presented individual animal data, and Study 6 additionally provided raw behavioral data in the supplementary materials. Presentation of individual animal data alongside group summary statistics can improve transparency and facilitate interpretation of variability, response distributions, and potential outliers. Such reporting may also support cross-study comparisons and aid efforts to evaluate reproducibility across experimental settings.

Third, an important observation from this study was that we did not detect robust GWI-like behavioral phenotypes in rats despite modifying the induction protocol to increase either chemical exposure intensity (Study 1b) or stress exposure (Study 1c). These findings suggest that, under the experimental conditions evaluated here, increasing chemical dose or stress level alone was insufficient to reproduce previously reported behavioral outcomes. However, because these variables were not systematically isolated and directly compared, we cannot exclude the possibility that they contributed to differences across studies.

The timing of behavioral assessment is also unlikely to fully explain the observed findings. In Study 1c (low-dose/high-stress), behavioral testing was repeated at 6–8 months post-exposure following the initial assessment at approximately 3 months post-exposure, and robust GWI-like behavioral phenotypes were still not detected.

The strain and source of rats are important considerations when interpreting variability across GWI studies. Of the 12 studies summarized in Table 3, 10 used Sprague–Dawley rats and two used Wistar rats. Among the Sprague–Dawley studies, rat sources included Charles River (n = 2), Harlan/Envigo (n = 5), Taconic Farms (n = 1), and two studies that did not report the source.

Although Sprague–Dawley rats are widely used in preclinical research, increasing evidence indicates that animals obtained from different vendors represent genetically and physiologically distinct populations rather than interchangeable stocks. Charles River and Harlan/Envigo Sprague–Dawley rats have undergone separate breeding histories and exhibit measurable differences in genetic background, physiology, metabolism, and responses to experimental challenges [33]. Such vendor-dependent differences have been reported to influence behavioral phenotypes, pharmacological responses, susceptibility to neurological injury, and other biological outcomes [34].

In the present study, rats were obtained from Charles River Laboratories, whereas several previous studies reporting GWI-like behavioral phenotypes used Sprague–Dawley rats obtained from Harlan/Envigo. Therefore, we cannot exclude the possibility that vendor-related differences contributed to differences in observed outcomes across studies. However, because our study did not directly compare rat vendors under identical experimental conditions, our data do not support concluding that Charles River Sprague–Dawley rats are inherently unsuitable for GWI modeling or that vendor source alone explains the observed outcomes. Additional factors—including housing conditions, animal handling procedures, environmental conditions, exposure paradigms, timing of behavioral testing, and other laboratory-specific variables—may also influence phenotype expression.

Our study employed a 5-day-per-week exposure schedule rather than continuous daily exposure protocols used in several previous studies. This schedule was selected to maintain consistency in animal handling, chemical administration, monitoring, and husbandry procedures throughout a prolonged study period. Nevertheless, intermittent exposure may differ from continuous exposure in cumulative exposure burden, pharmacokinetic profiles, recovery between dosing periods, and temporal development of behavioral and biological responses. Therefore, we cannot exclude the possibility that this procedural difference contributed to variability in phenotype expression.

Another procedural consideration is the source of chemicals used for exposure. Although PB, DEET, and permethrin were obtained from research-grade commercial suppliers, we did not independently verify purity, stability, or potency. Therefore, lot-to-lot or vendor-related variation cannot be excluded as a contributor to differences in effective exposure across studies.

Finally, post-exposure housing conditions following topical chemical administration may also influence effective exposure. In the present study, animals were individually transferred to the procedure room under ambient environmental conditions for chemical administration and subsequently returned to their home cages and allowed to remain undisturbed for 5 minutes to permit initial dermal absorption. No additional airflow or procedural intervention was applied during this post-exposure period. Nevertheless, because dermal retention and systemic exposure were not directly measured, we cannot exclude the possibility that post-exposure environmental or husbandry factors contributed to variability across studies.

It is important to note that the power analyses presented in the Results section were based on detecting large effect sizes and were informed by our previous findings in mice [20]. Accordingly, although the present study was adequately powered to detect large effects, it was underpowered to detect medium-sized effects. Therefore, the absence of statistically significant behavioral differences should not be interpreted as evidence that smaller effects were absent. In addition, power calculations were not reported in most of the studies summarized in Table 3, which may make direct comparison of statistical sensitivity across studies difficult.

Our pathological analyses showed trends toward reduced neurogenesis in the DG and increased GFAP immunoreactivity in the CA1 and CA3 regions of the hippocampus following high-stress/low-dose exposure; however, these changes did not reach statistical significance (Fig 4). Therefore, the relationship between these neurobiological changes and GWI-like phenotype expression remains unclear. Although neurogenesis impairment and astrogliosis have been proposed in experimental models of GWI, evidence linking these changes directly to clinical GWI pathology remains limited. It is possible that the observed changes did not reach a sufficient threshold to produce measurable behavioral alterations under the conditions tested. Neurobiological alterations may precede the development of detectable behavioral deficits or persist in the absence of overt behavioral abnormalities, and the behavioral assays employed in the present study may not have been sufficiently sensitive to detect subtle functional impairments. These interpretations remain speculative and warrant further investigation in future longitudinal studies.

In addition, high-stress/low-dose exposure increased VGLUT1 and EAAT2 expression, suggesting concurrent changes in glutamatergic signaling and glutamate clearance capacity. One possible interpretation is that increased EAAT2 expression reflects a compensatory response that helps maintain glutamate homeostasis and limits downstream excitotoxic effects. Such adaptive mechanisms may have attenuated the emergence of detectable behavioral phenotypes in the present study. Alternatively, subtle functional deficits may have been present but remained undetected.

In our previous mouse study, we observed significant GWI-like phenotypes [20]; however, these phenotypes were not observed in rats, despite using the same exposure paradigm. This discrepancy may reflect species-specific differences in stress responsivity, pharmacokinetics, and behavioral sensitivity.

In conclusion, our findings should not be interpreted as contradicting or invalidating previously published GWI studies. Rather, under the experimental conditions used in the present study—including animal source and strain background, exposure paradigm, and laboratory environment—we did not detect GWI-like behavioral phenotypes across multiple chemical and stress exposure conditions. Differences in vendor background, exposure schedules, housing conditions, and other laboratory-specific variables may contribute to variability across studies and should be evaluated directly in future validation efforts.

Because *in vivo* testing in animal models remains an essential preclinical step and a regulatory requirement prior to human clinical trials, continued efforts to validate, optimize, and standardize reproducible GWI animal models will be important for strengthening translational rigor and supporting therapeutic development.

## Supporting information

S1 File Behavioral raw data. Behavioral raw data for all groups of rats for all behavioral tests including, open field, dark and light, elevated plus maze, novelty-suppressed feeding, novel object location, novel object recognition, and Barnes maze.
(XLSX)

S2 File Immunohistochemical raw data. Immunohistochemical raw data and representative images for the number of antibody positive cells per area for glial fibrillary acidic protein, doublecortin, and parvalbumin.
(PDF)

S3 File Western blotting raw data. Western blotting raw data with images. Western blots were analyzed by choosing an exposure time that was below saturation for each respective protein.
(PDF)

S4 File Statistical data. The following data are included: test statistics, degrees of freedom, significance level, effect size, confidence interval, and descriptive statistics.
(PDF)

S5 File Analysis of pooled data for low dose/low stress exposure study. (DOCX)

S6 File Analysis of pooled data for low dose/high stress exposure study. (DOCX)
