# Supplementary material for: Limited detection of Gulf War illness-like behavioral phenotypes in Charles River male Sprague-Dawley rats across multiple chemical and stress exposure paradigms
Source: PLoS One. 2026 Sep 30;21(9):e0337157. doi: 10.1371/journal.pone.0337157 (PMC13626346; doi:10.1371/journal.pone.0337157)

Glial fibrillary acidic protein – Dentate Gyrus – Raw Data and Representative Images

GFAP+/Area(μm^2) x10^{-5}

Control Veh	Control LH-001	GWI Veh	GWI LH-001
102.8499	77.62254	13.58394	62.09803
42.69239	67.91972	38.81127	64.03859
75.68197	77.62254	27.16789	13.58394
50.45465	71.80084	60.15746	67.91972
3.881127	58.2169	21.3462	19.40563
64.03859	67.91972	42.69239	42.69239
25.22732	60.15746	25.22732	58.2169
50.45465	52.39521	69.86028	44.63296
15.52451	9.702817	44.63296	54.33577
23.28676	83.44422	42.69239	32.98957
42.69239	17.46507	17.46507	31.04901
60.15746	46.57352	42.69239	15.52451
40.75183	31.04901	31.04901	48.51408
60.15746	27.16789	62.09803	48.51408
13.58394	58.2169	42.69239	50.45465
44.63296	34.93014	67.91972	27.16789
36.8707	50.45465	58.2169	60.15746
29.10845	42.69239	64.03859	48.51408
64.03859	69.86028	48.51408	9.702817
36.8707	48.51408	58.2169	11.64338
50.45465	40.75183	50.45465	56.27634
38.81127	48.51408	32.98957	48.51408
64.03859	27.16789	34.93014	29.10845
50.45465	50.45465	48.51408	52.39521
25.22732	17.46507	29.10845	7.762253
19.40563	42.69239	11.64338	36.8707
23.28676	52.39521	52.39521	52.39521
1.940563	44.63296		54.33577
0	29.10845		54.33577
42.69239	19.40563		71.80084
			25.22732
			52.39521
			36.8707

Control Vehicle

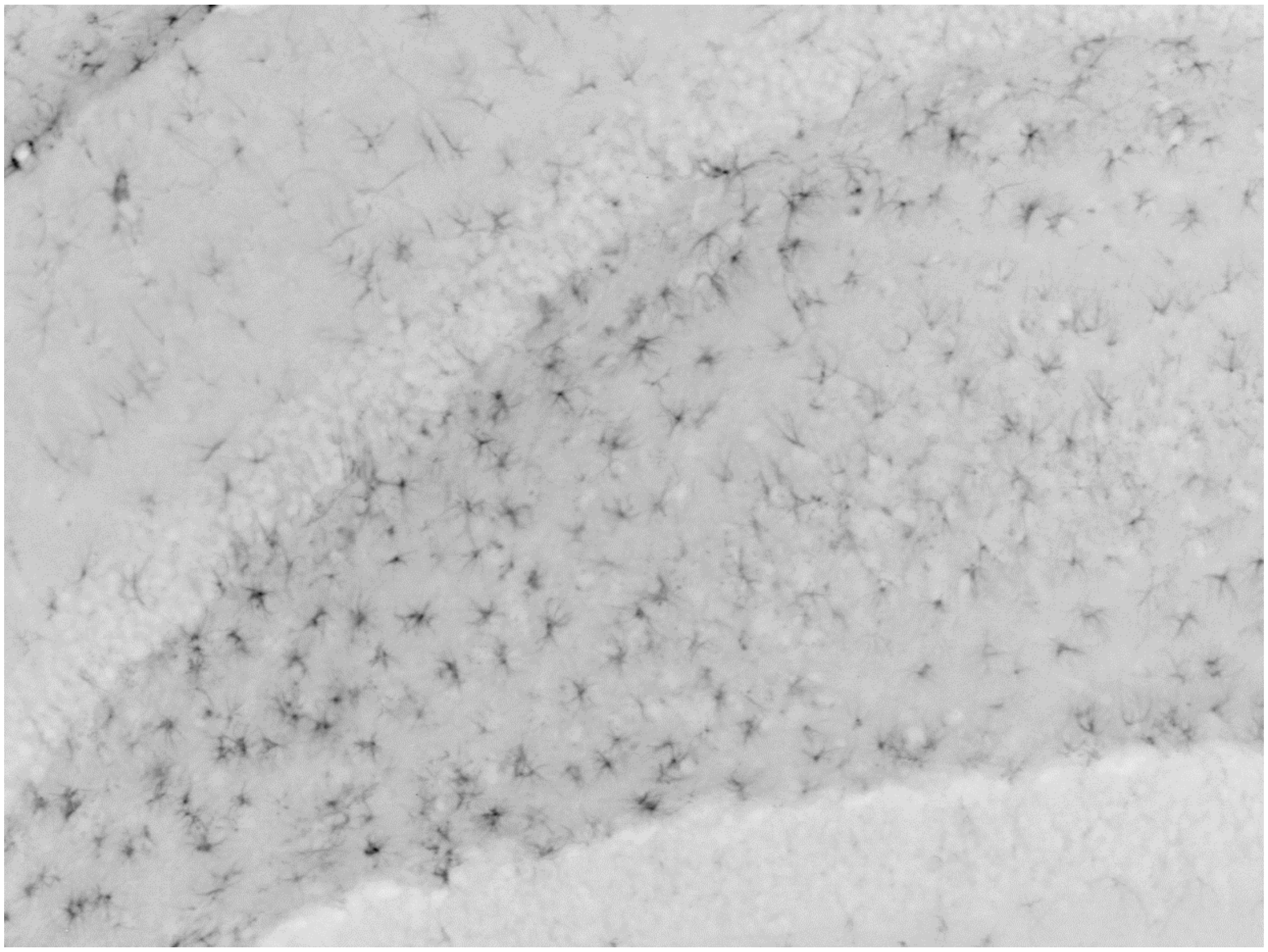

Control LH-001

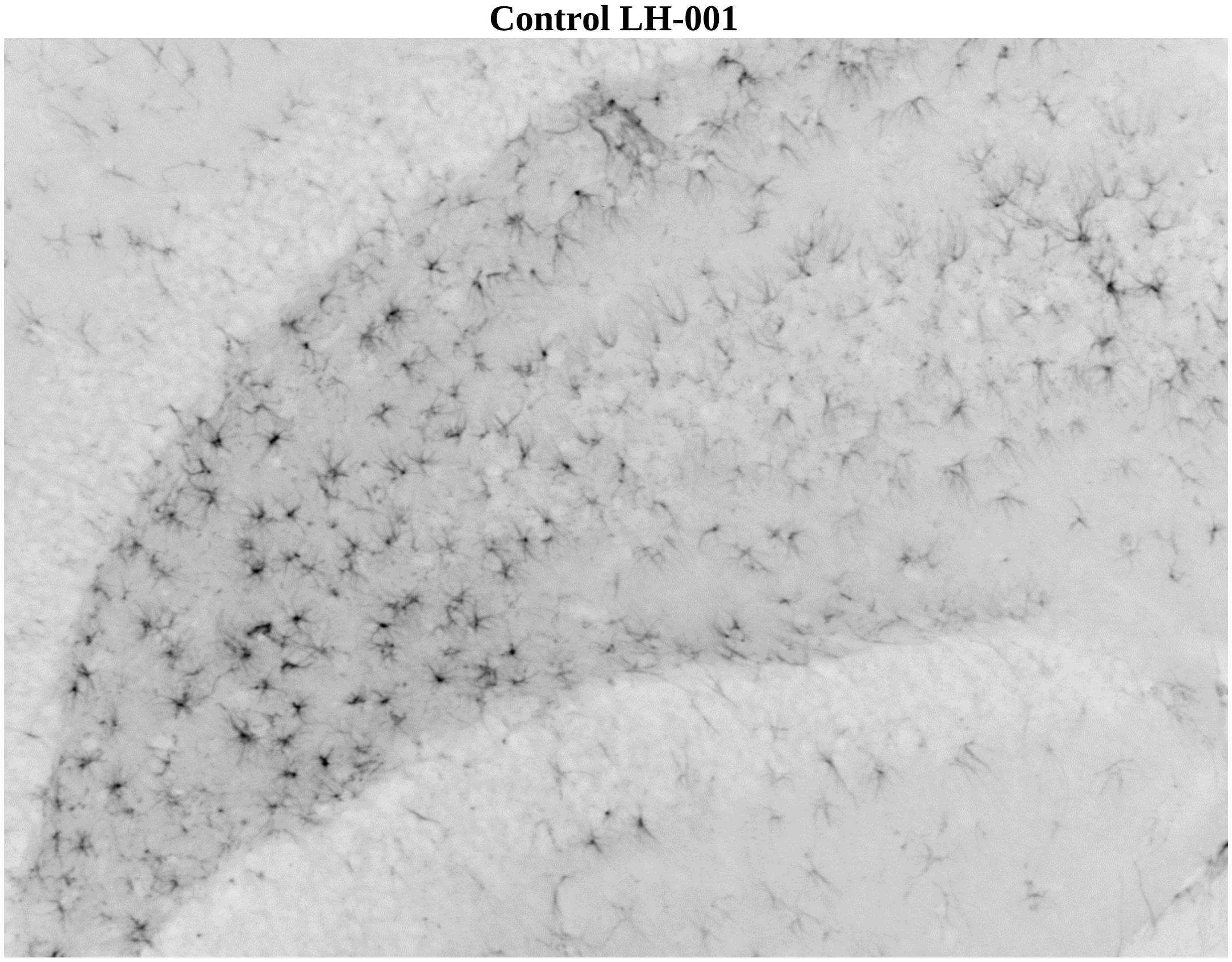

GWI Vehicle

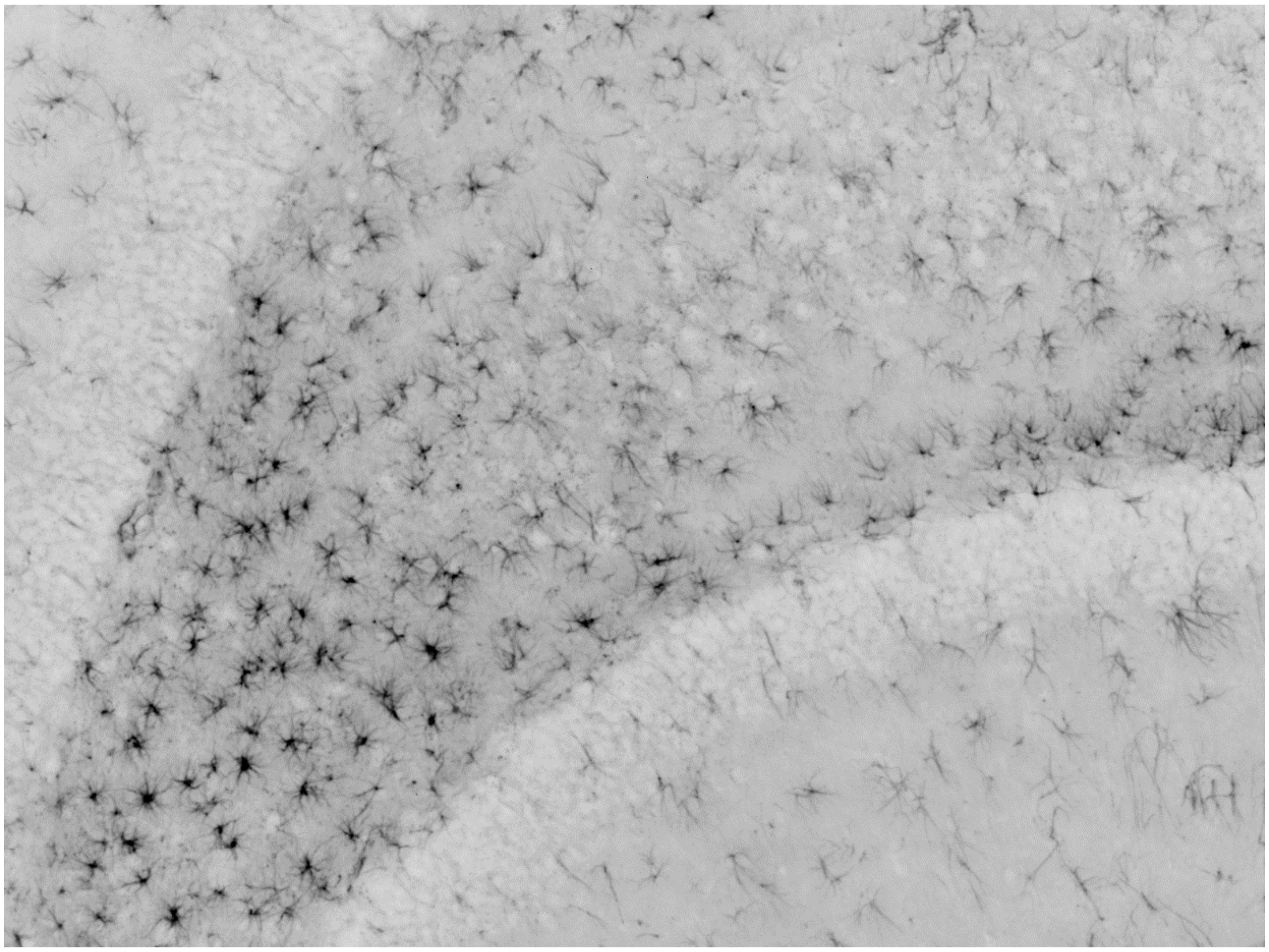

GWI LH-001

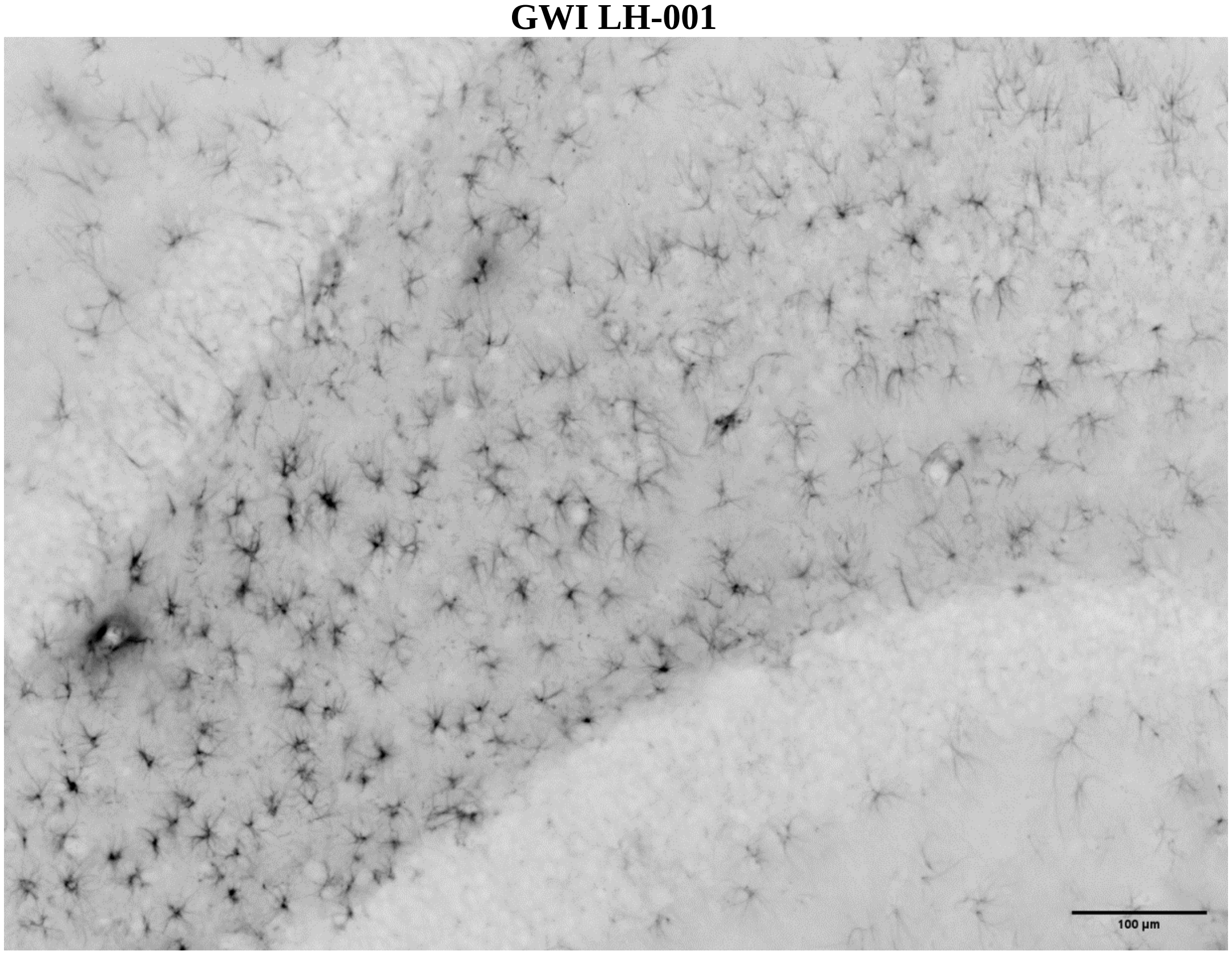

Glial fibrillary acidic protein – CA1 – Raw Data and Representative Images

GFAP+/Area(μm^2) x10^{-5}

Control Veh	Control LH-001	GWI Veh	GWI LH-001
12.41961	19.79375	25.22732	17.85318
12.80772	32.60146	22.12242	23.28676
16.68884	21.73431	19.01752	13.97206
8.150366	23.67487	16.30073	20.18186
21.73431	22.51053	9.702817	7.374141
19.01752	28.72034	21.73431	15.52451
10.09093	14.36017	20.56997	14.74828
11.64338	10.09093	19.01752	23.67487
10.09093	13.58394	15.52451	13.19583
14.74828	1.940563	18.24129	9.702817
17.46507	3.104901	9.702817	13.19583
11.25527	5.433578	5.433578	13.19583
10.47904	9.702817	5.045465	33.37769
8.538479	2.328676	17.85318	28.33222
19.01752	2.328676	15.91262	13.19583
1.552451	9.314704	25.22732	14.74828
17.07696	3.104901	19.79375	3.104901
5.045465	7.374141	10.09093	10.47904
7.374141	7.374141	17.85318	12.41961
10.86716	12.03149	29.10845	0.7762253
9.314704	7.762253	5.433578	10.09093
20.56997	17.85318	6.597915	10.09093
8.150366	14.74828	15.52451	18.24129
11.25527	9.314704	16.68884	20.18186
12.03149	10.86716	18.62941	8.538479
15.52451	5.433578	13.58394	11.25527
4.269239	22.89865	3.881127	15.52451
16.68884	7.374141	10.47904	12.80772
3.104901	2.716789	17.07696	11.25527
13.58394	3.493014	9.314704	14.74828
24.06298	8.150366	6.597915	14.36017
8.926591	3.493014		13.19583
24.83921	5.82169		4.269239
13.19583			13.58394

Control Vehicle

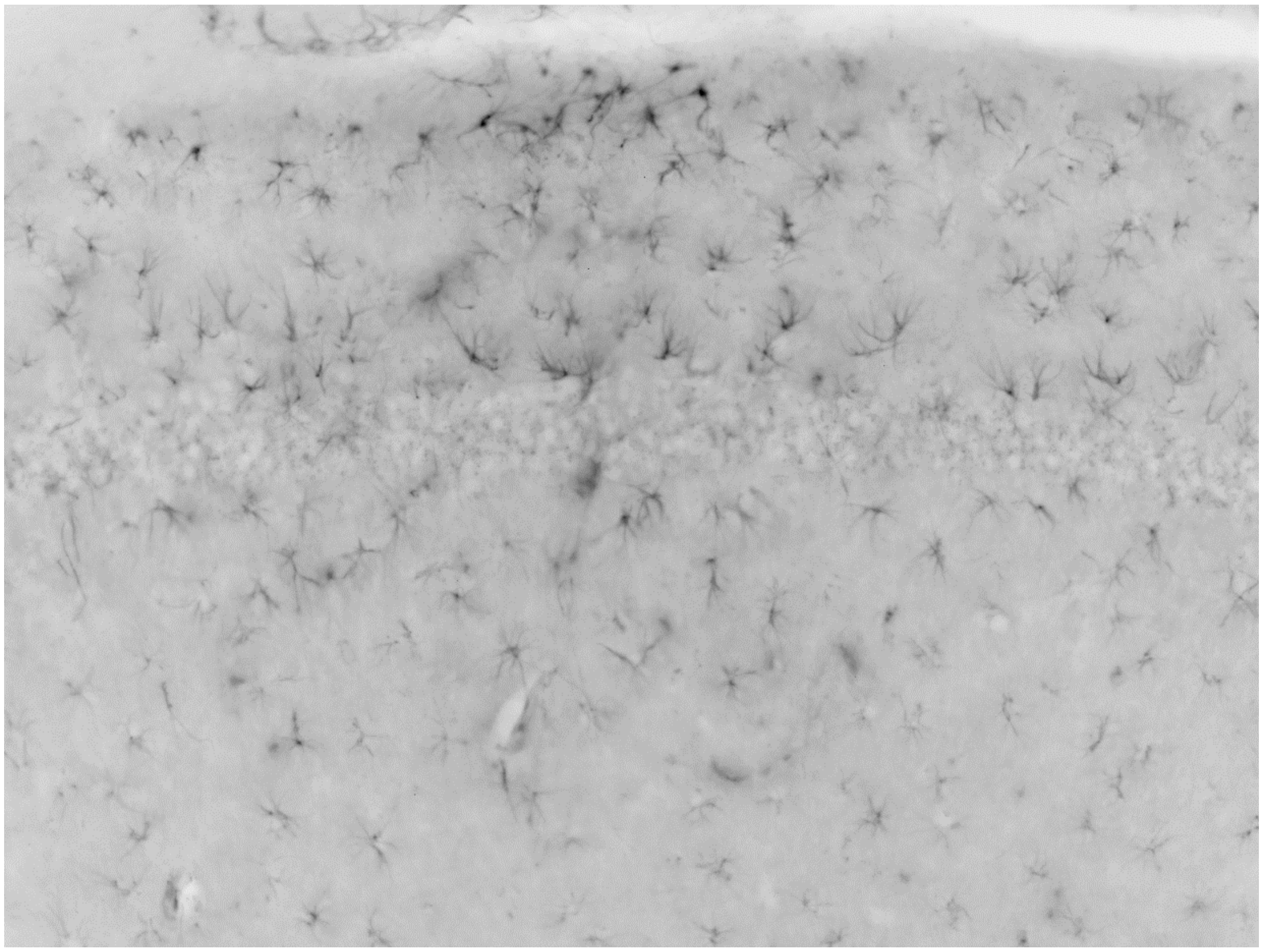

Control LH-001

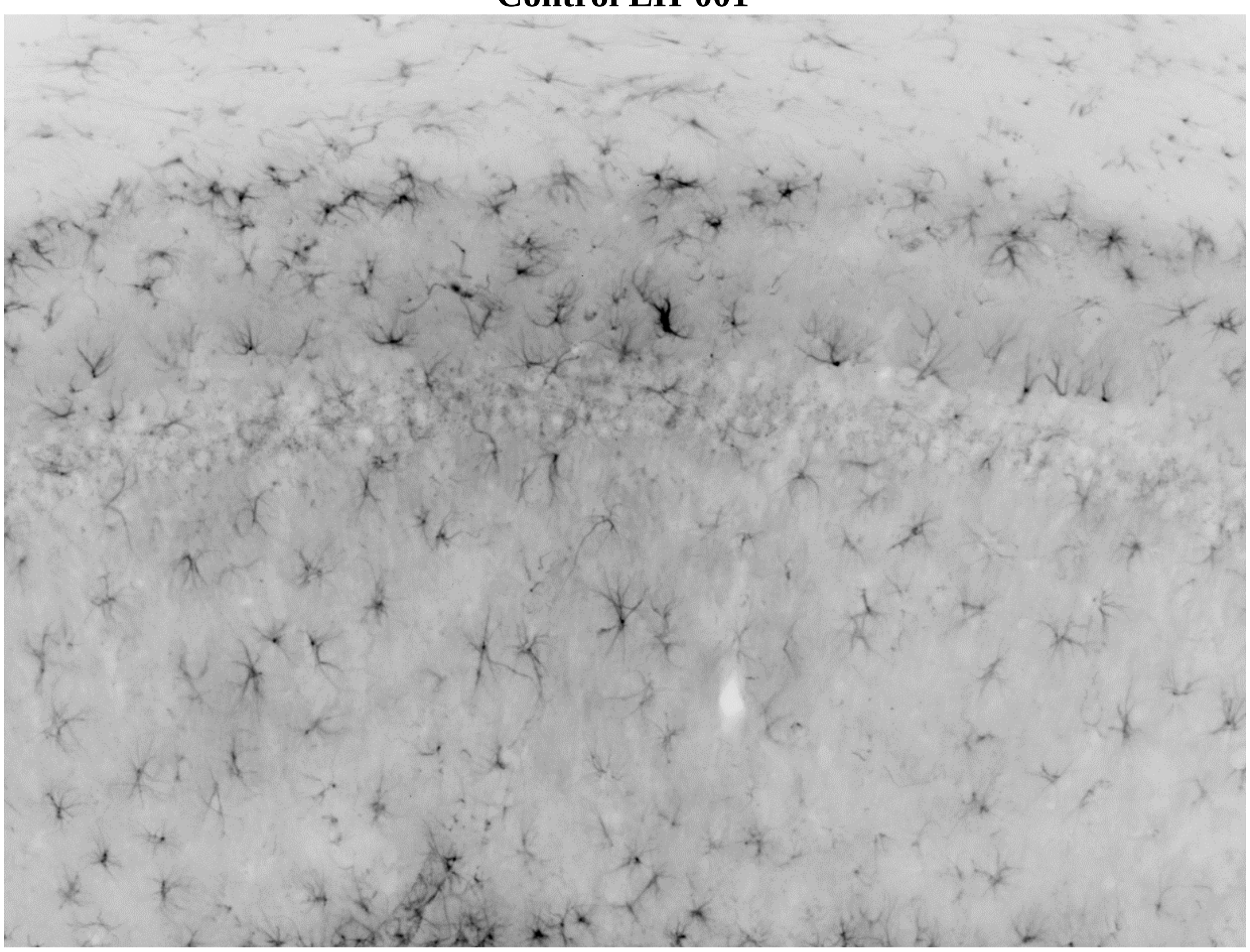

GWI Vehicle

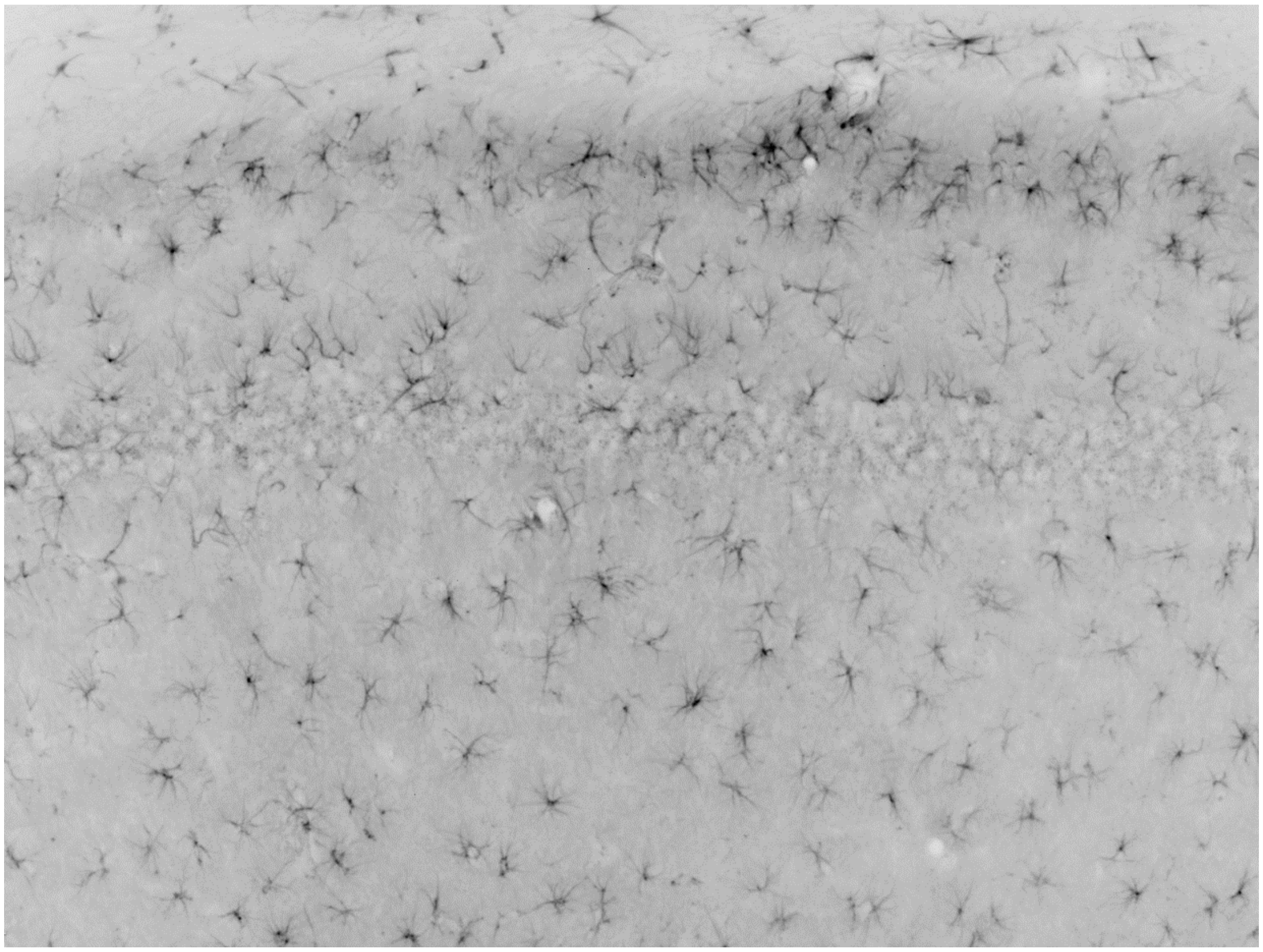

GWI LH-001

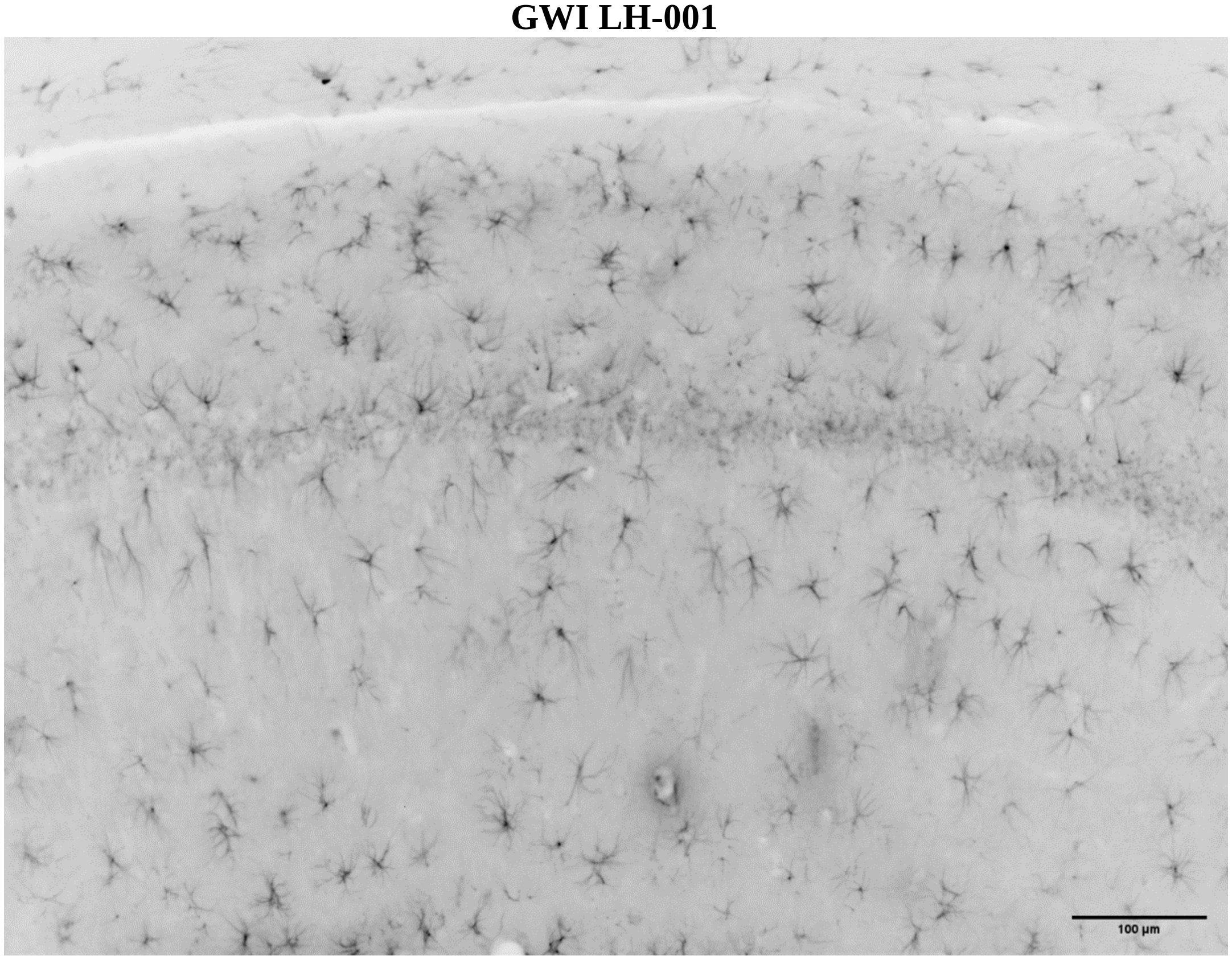

Glial fibrillary acidic protein – Dentate Gyrus – Raw Data and Representative Images
GFAP+/Area(µm^2) x10^{-5}

Control Veh	Control LH-001	GWI Veh	GWI LH-001
14.48954	13.97206	14.48954	25.87418
27.94411	13.97206	22.25179	11.38464
25.35669	11.90212	19.66438	13.45457
7.762253	13.45457	12.41961	10.86716
7.24477	11.90212	13.97206	15.52451
13.45457	7.24477	22.25179	25.35669
13.97206	23.28676	11.38464	16.55947
11.38464	5.692319	17.07696	16.55947
10.86716	16.55947	19.14689	12.93709
17.59444	54.85326	11.90212	8.79722
25.35669	10.86716	12.93709	12.93709
26.39166	18.62941	15.00702	43.9861
12.93709	18.11193	16.04199	15.52451
10.34967	6.209803	14.48954	14.48954
13.45457	23.28676	7.24477	12.41961
16.55947	26.39166	7.24477	15.52451
11.90212	33.11895	10.86716	18.62941
37.7763	22.76928	9.832188	10.34967
15.52451	21.21683	3.622385	4.139868
20.18186	3.622385	3.104901	18.62941
17.07696	16.04199	5.692319	12.93709
22.76928	22.25179	4.657352	8.279737
62.61551	9.314704	12.41961	15.52451
21.21683	9.314704	12.41961	10.86716
11.90212	7.762253	12.41961	6.209803
23.80424	21.21683	10.86716	8.79722
9.314704	19.14689	12.41961	24.32173
9.314704	8.279737	8.79722	12.41961
13.97206	26.39166	20.69934	12.93709
5.692319	21.73431	5.174836	17.07696
19.14689	2.069934	9.314704	10.34967
26.90915	25.35669		19.14689
19.14689	11.90212		48.12597
16.04199			12.93709
5.174836			11.38464
			5.174836

Control Vehicle

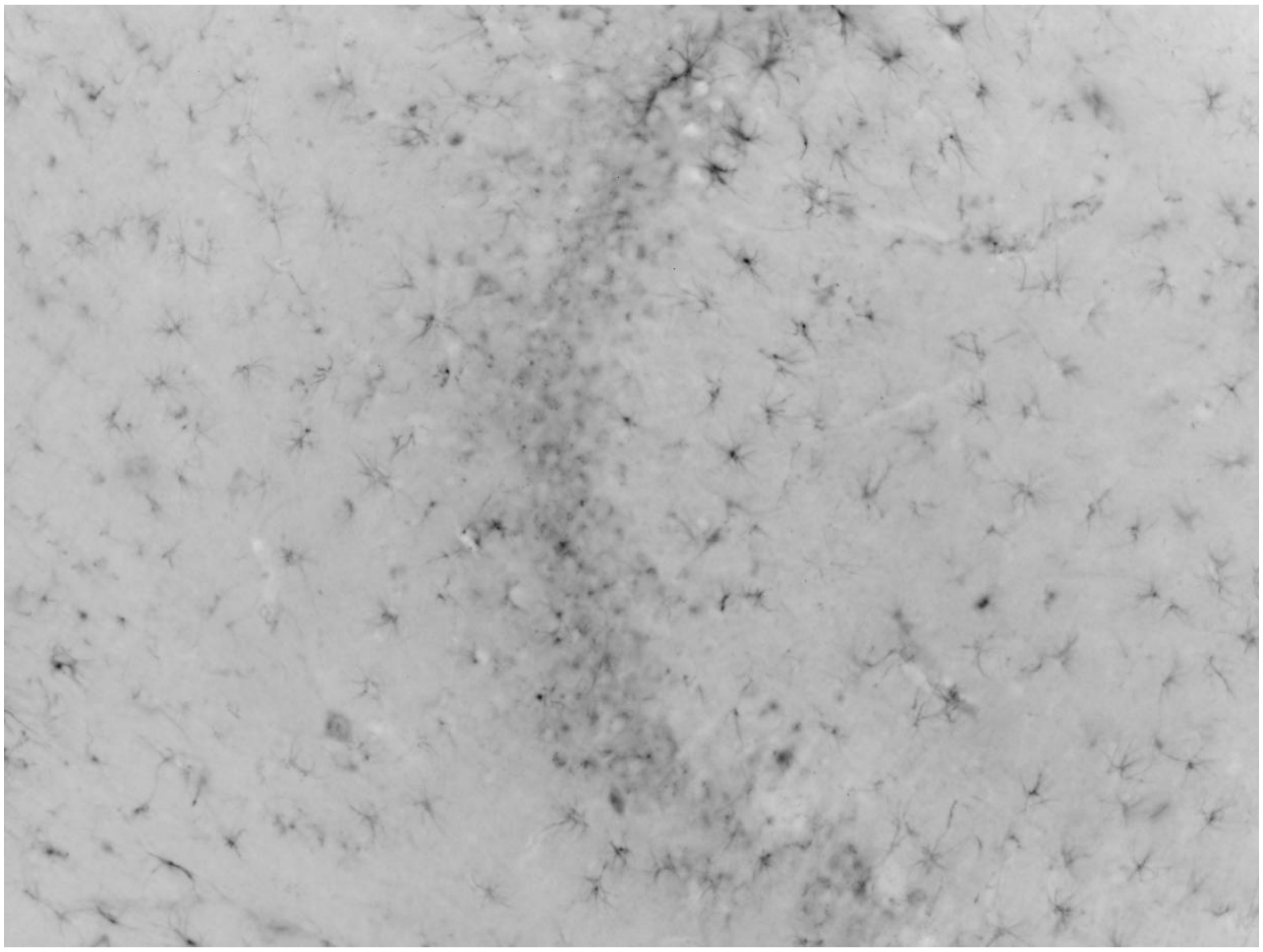

Control LH-001

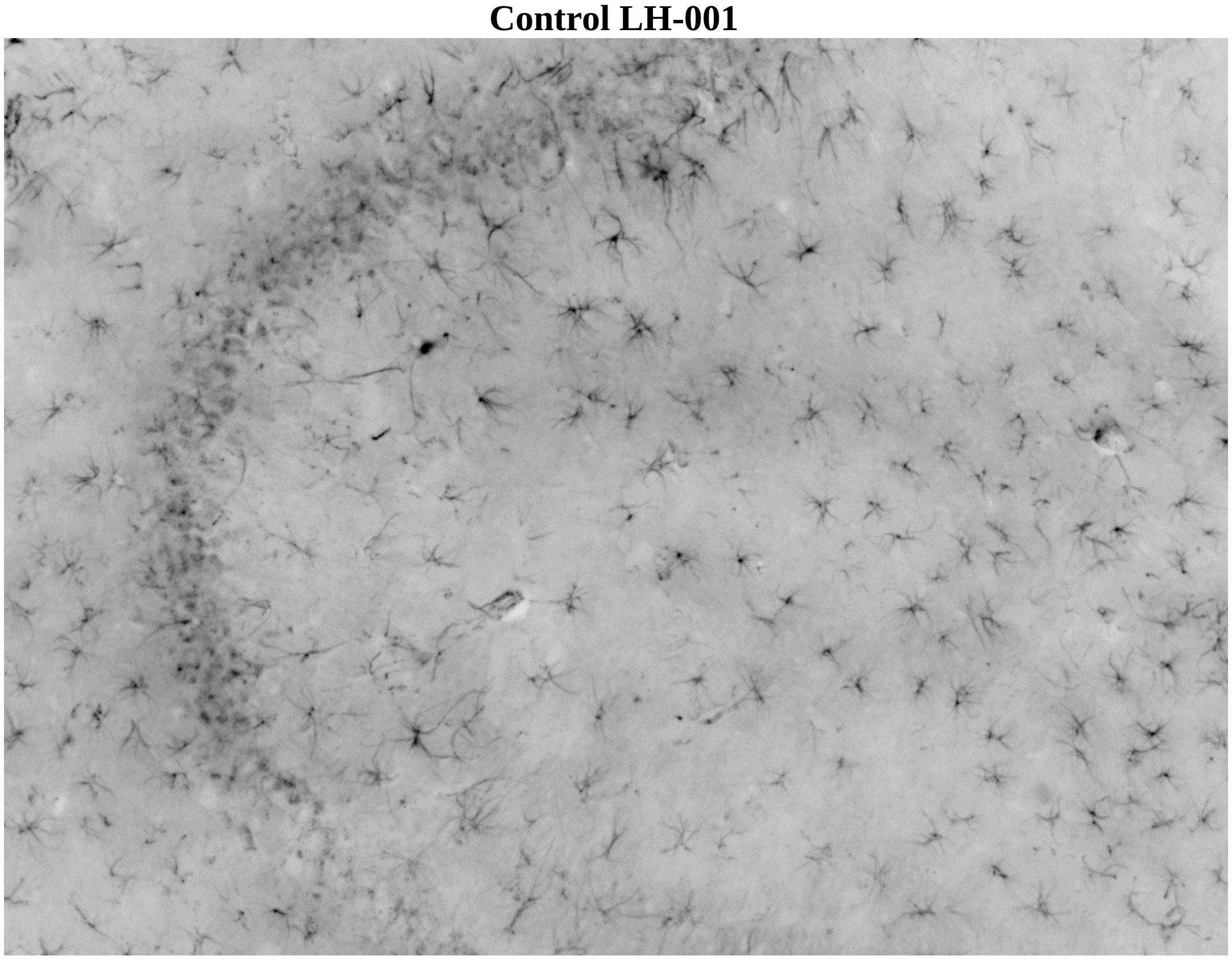

GWI Vehicle

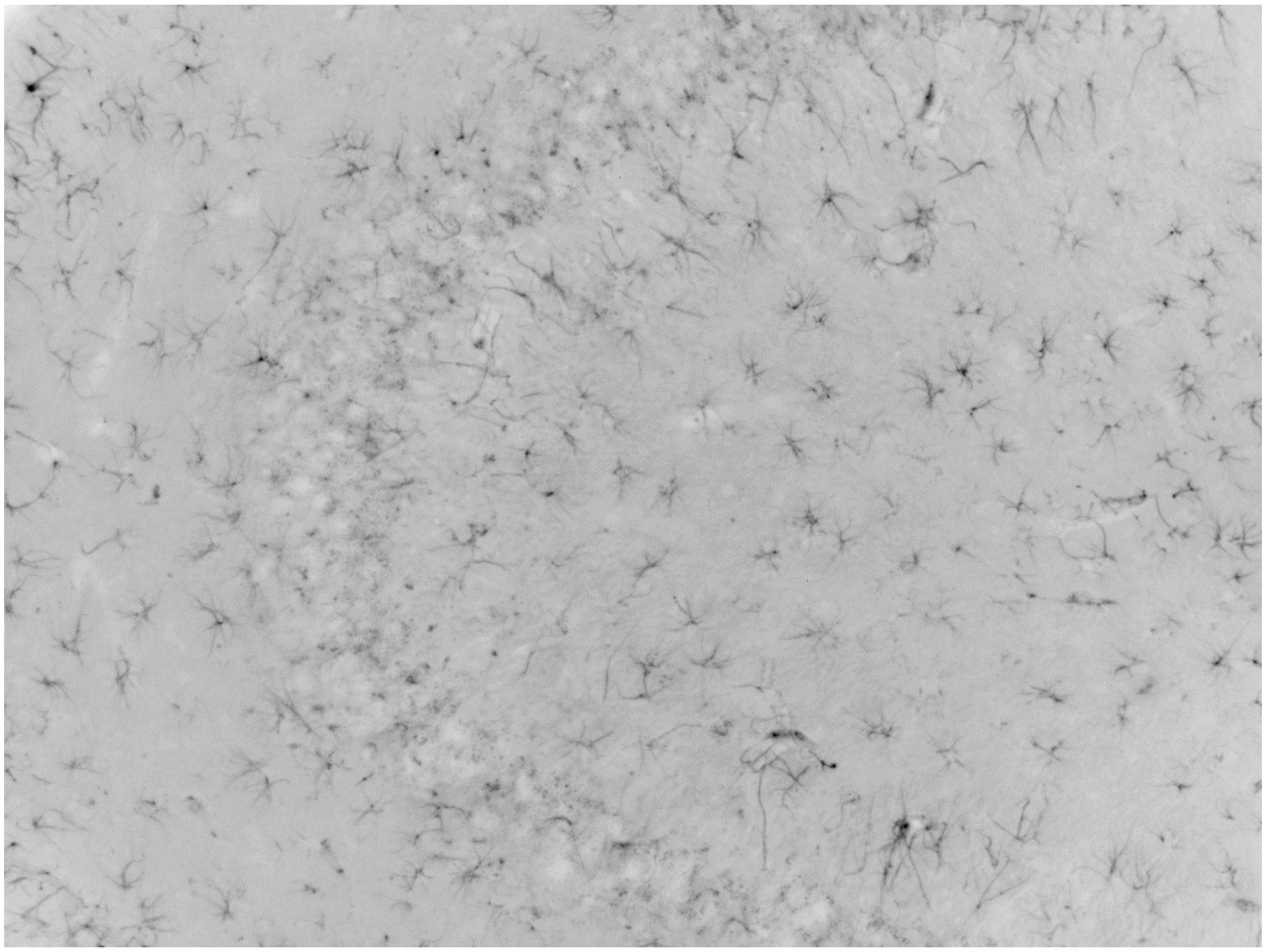

GWI LH-001

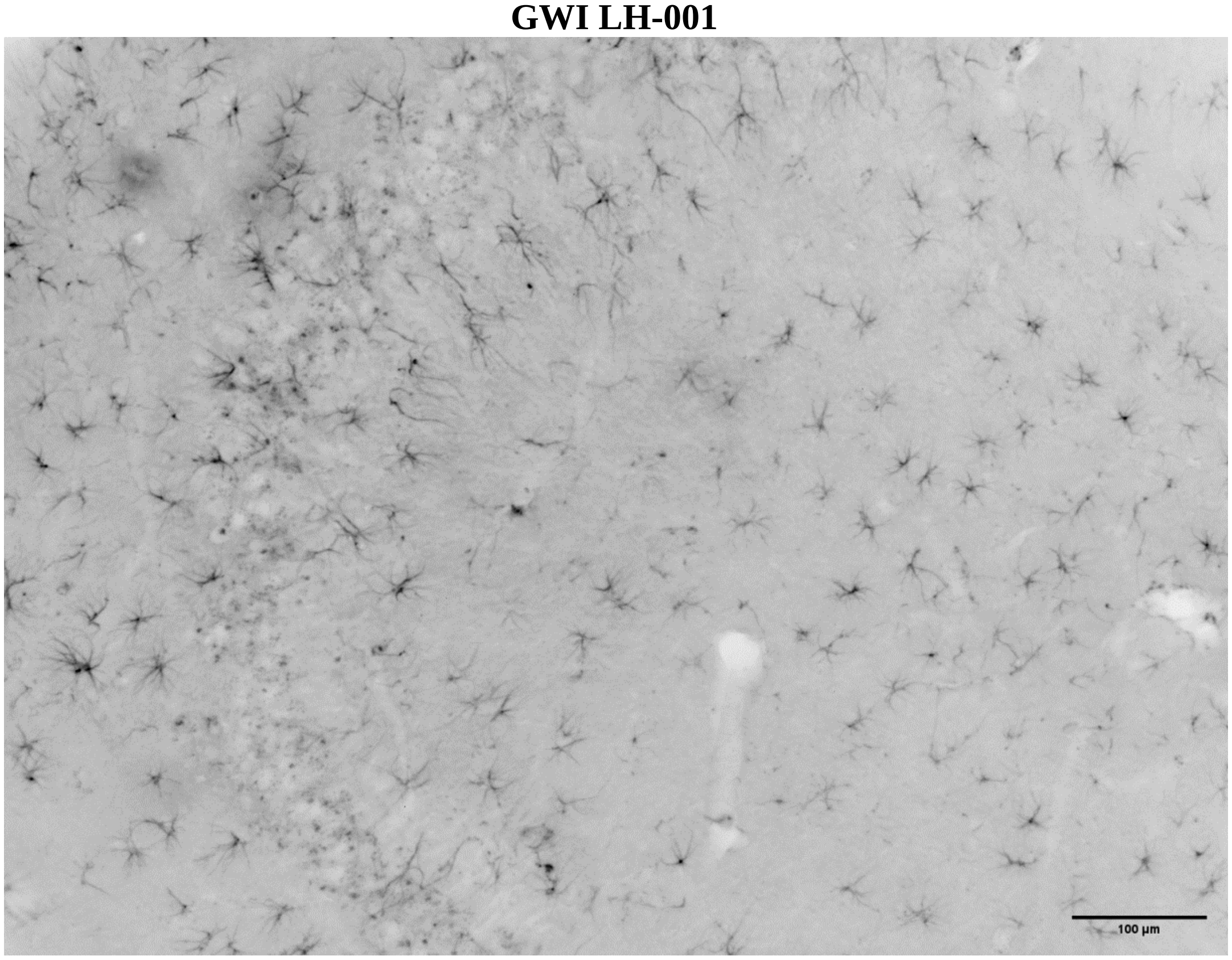

Parvalbumin – Whole Hippocampus – Raw Data and Representative Images
PV+ cells/Area(µm^2) x10^{-6}

Control Veh	Control LH-001	GWI Veh	GWI LH-001
17.2003	21.74357	13.03262	9.54098
15.93532	21.26717	15.3793	8.584421
17.16353	14.40763	14.92428	9.134063
16.62642	11.28147	13.0569	12.07681
14.52074	9.117399	11.09114	9.951571
11.84214	13.31412	17.13994	13.71204
17.12773	9.151898	13.63195	31.13176
19.09971	9.951012	13.54153	17.75727
20.62801	16.29167	14.10239	16.41654
18.20044	14.47285	20.91762	25.30467
25.12361	11.95089	13.41933	30.24318
24.1441	17.35417	13.28895	14.44395
16.04734	35.08112	13.58904	10.483
17.46757	24.23178	17.46016	13.22237
19.11838	19.81625	18.3178	17.04617
16.22452	21.23121	15.66116	26.21428
12.07192	25.25	15.55513	19.90627
11.595	13.75879	12.10371	24.80256
13.71744	15.53825	29.15169	25.00715
16.52351	13.26747	33.30086	23.02906
8.452498	19.34946	22.66146	30.36105
10.45323	26.57792	19.82118	29.58932
13.41929		27.78439	18.87376
15.8408		31.7509	22.046
10.14263		25.74026	
		25.32865	

Control Vehicle

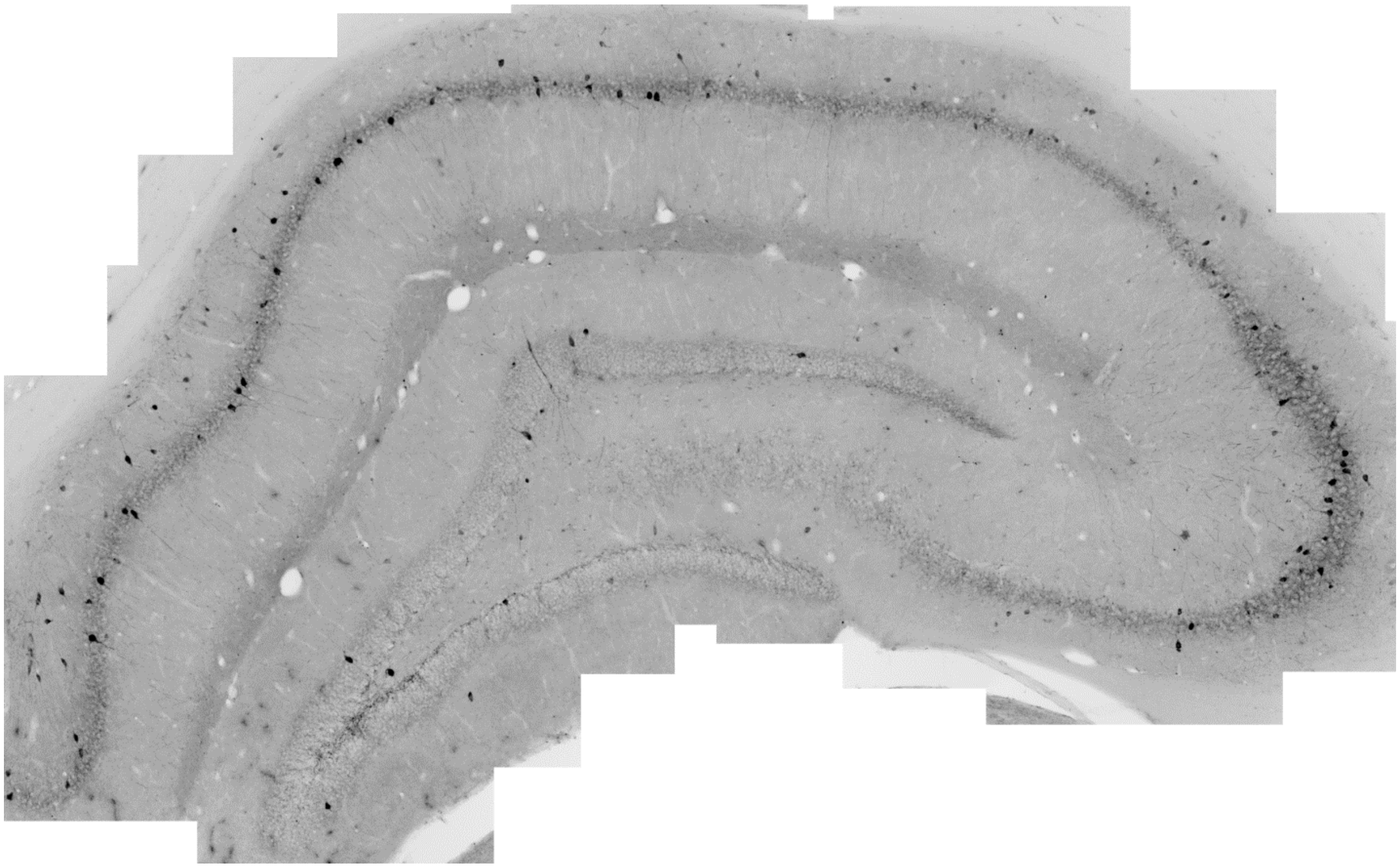

Control LH-001

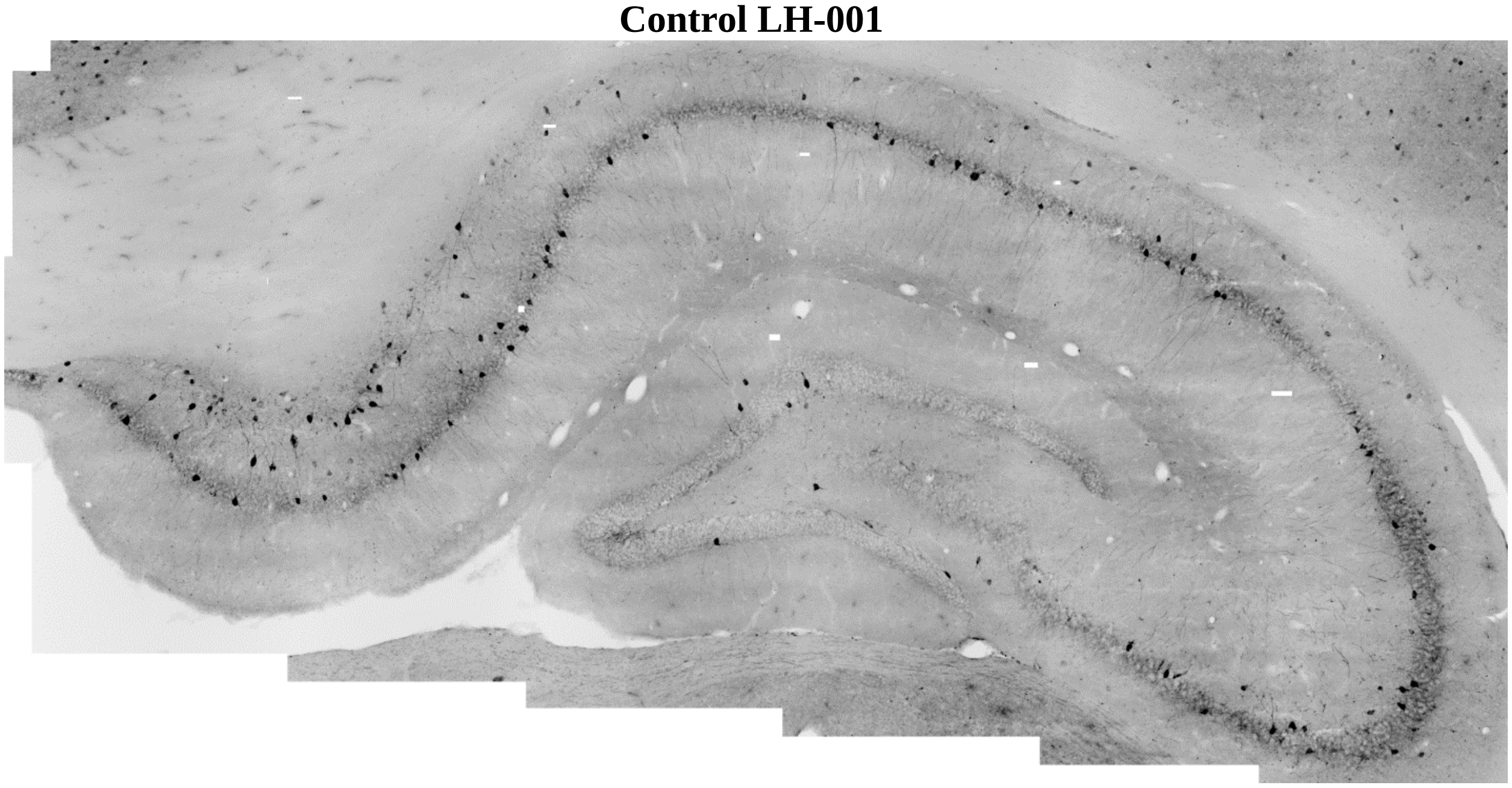

GWI Vehicle

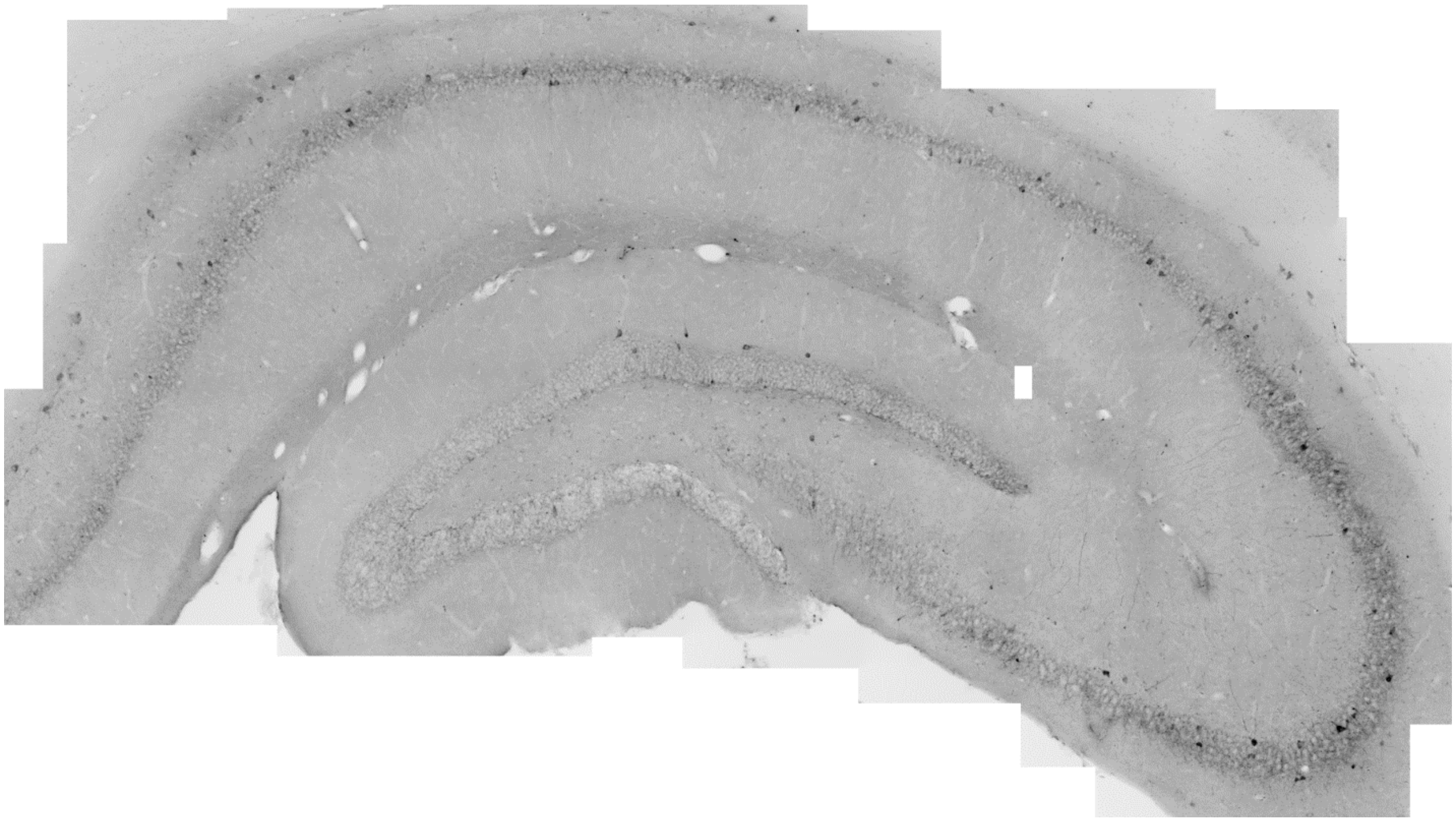

GWI LH-001

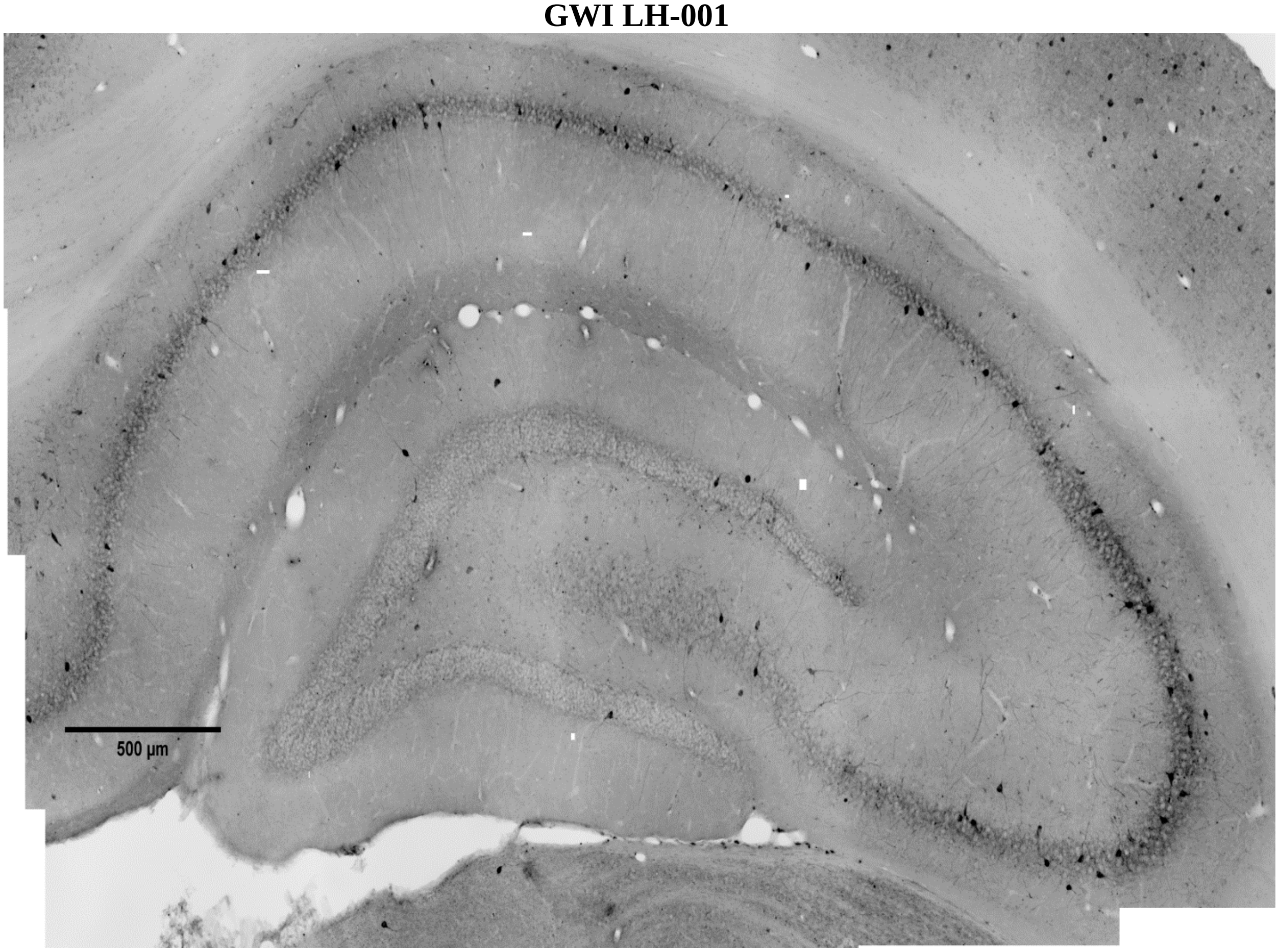

Doublecortin – Dentate Gyrus – Raw Data and Representative Images

DCX+ cells/Area(µm^2) x10^{-6}

Control Veh	Control LH-001	GWI Veh	GWI LH-001
16.141	17.41026	8.676302	14.8104
17.57588	16.97222	8.748119	8.423178
19.2173	18.318	12.18467	15.35135
17.79264	14.59678	14.93653	14.31747
19.6897	10.74798	9.81412	5.116599
11.59544	8.099475	12.2503	14.80261
14.57243	23.80614	12.98848	10.20632
25.75035	11.32046	13.0766	9.865423
19.84684	9.238694	20.33259	12.23207
26.8795	10.4619	15.69302	25.94129
19.72976	11.1216	16.21817	24.08164
19.46709	12.87661	15.74913	15.91915
19.81934	9.608036	15.21034	13.48874
19.42071	15.89347	13.59744	16.86805
17.83455	15.49856	9.011732	18.76106
12.10142	11.64377	13.82779	20.8831
12.82188	15.27879	12.02546	16.48584
14.82679	12.91885	28.55647	11.02998
15.588	15.57089	25.61961	12.38969
11.04675	11.80639	21.14669	16.14503
13.66627	19.11882	25.85266	14.0805
12.54541	49.69794	28.82826	10.24327
28.19417	48.33084	14.35952	10.22703
8.718869	34.9707	16.17801	12.32791
36.34464	19.5597	11.81686	11.9124
48.87075	27.15365	10.50171	3.65842
30.51757	30.86356	13.77716	14.56424
10.44163	36.26902	16.95664	9.060459
12.23912	23.46807		18.79635
11.62475	39.56897		
8.297615	33.06115		
16.43592	39.71786		
12.57338	26.53509		
	47.02454		
	37.39676		
	13.32501		
	10.92948		
	15.76995		
	15.44958		

Control Vehicle

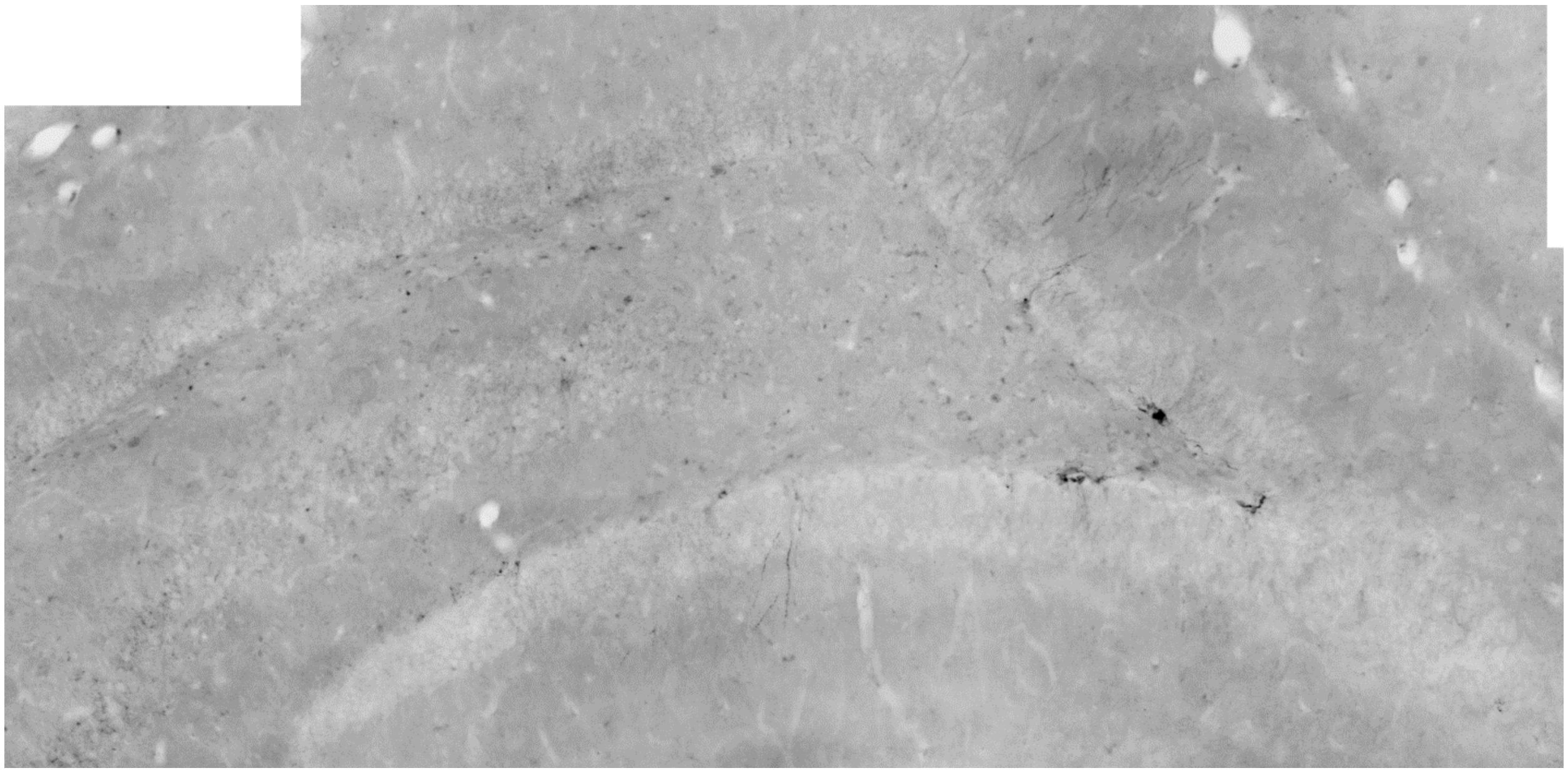

Control LH-001

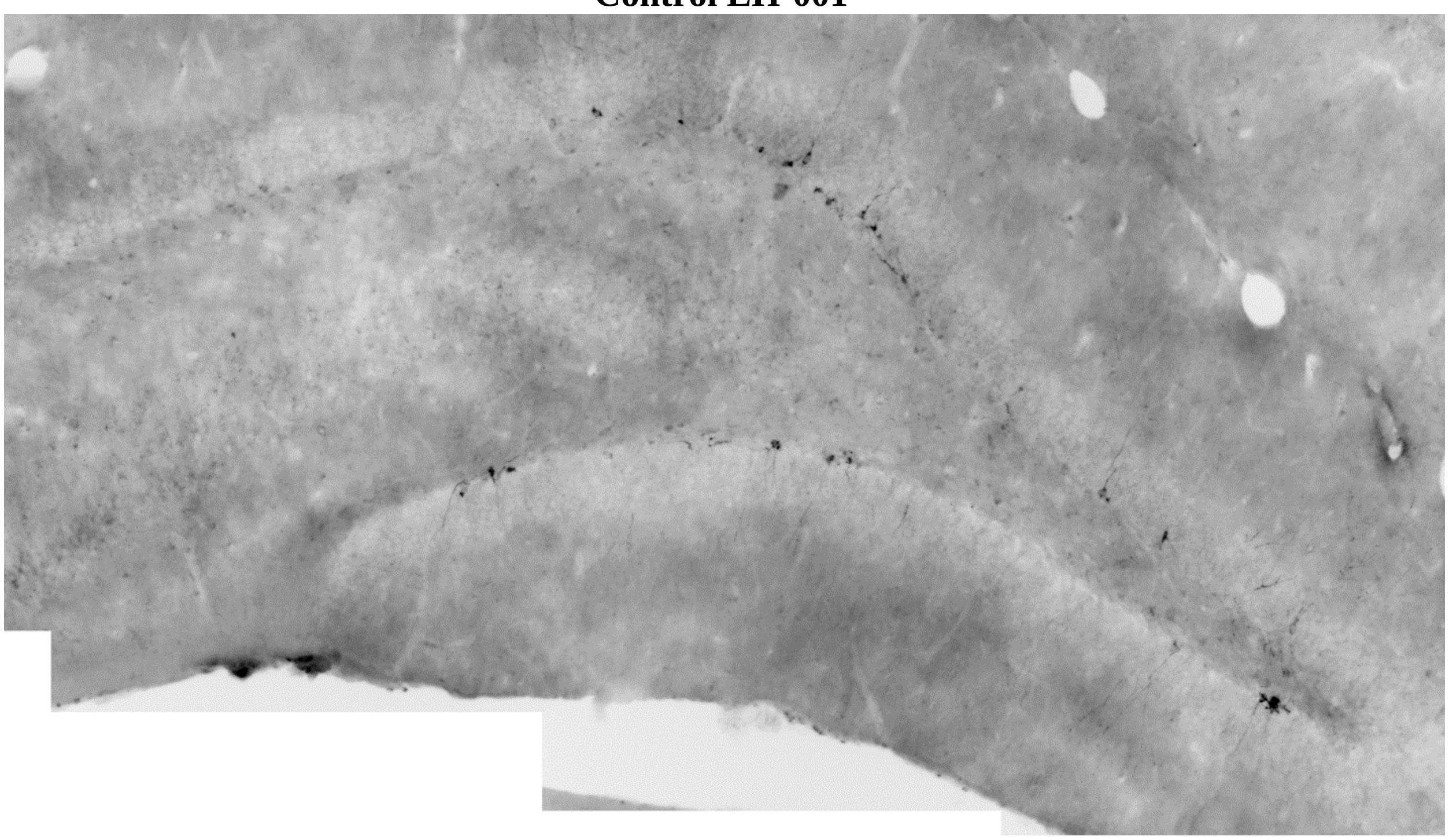

GWI Vehicle

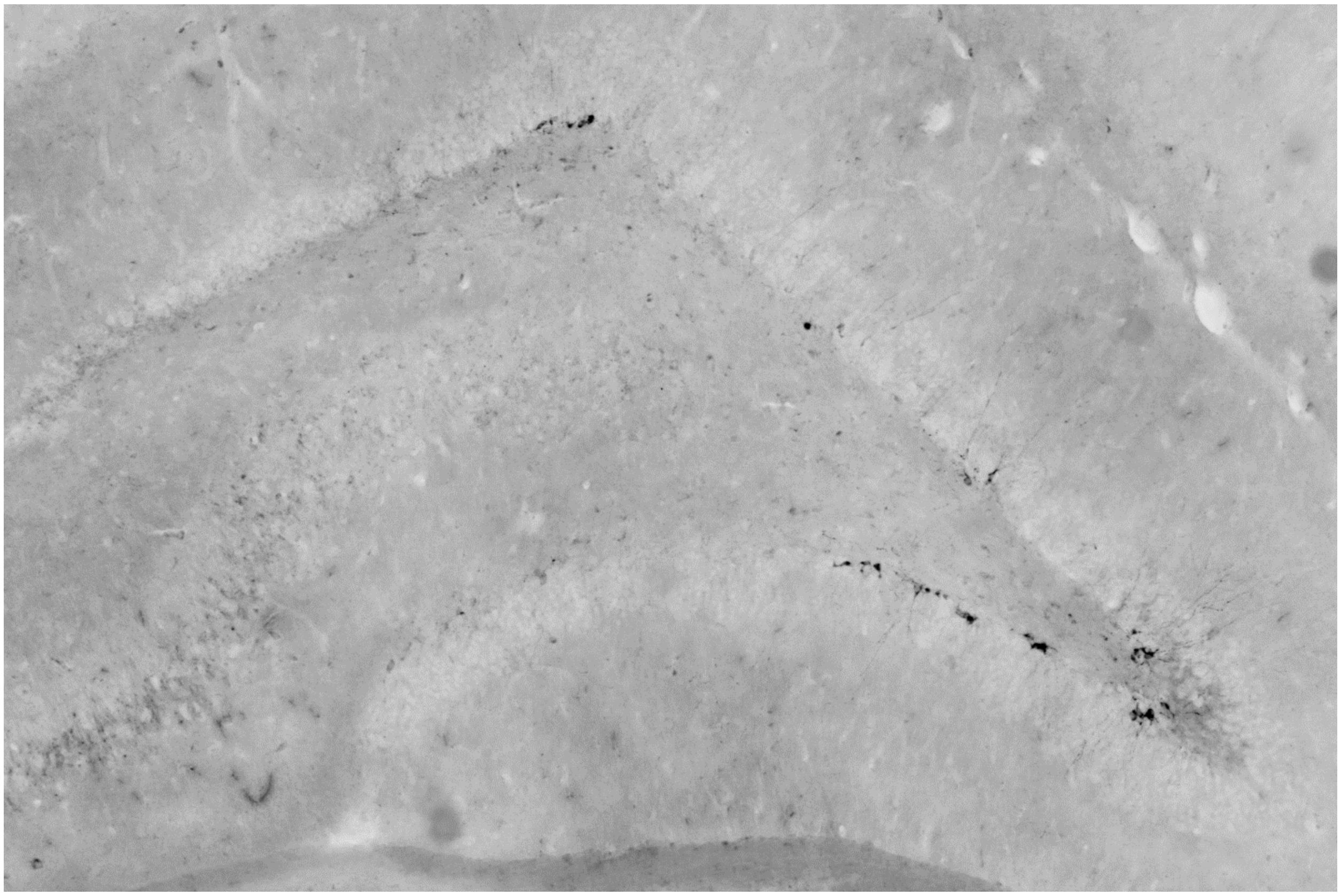

GWI LH-001

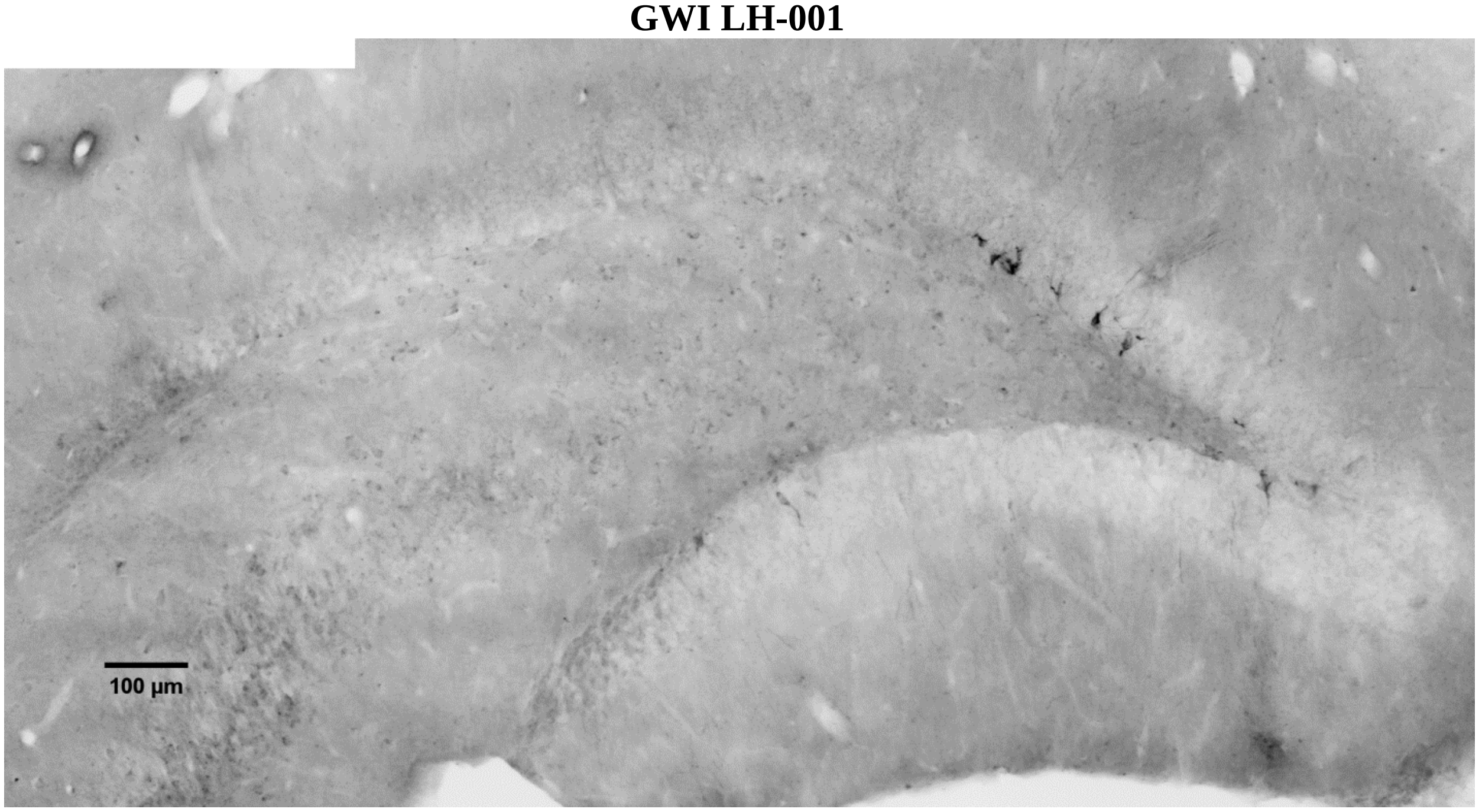

Supplement: Supplementary file 1 [file pone.0337157.s002.pdf]
