# Supplementary material for: Limited detection of Gulf War illness-like behavioral phenotypes in Charles River male Sprague-Dawley rats across multiple chemical and stress exposure paradigms
Source: PLoS One. 2026 Sep 30;21(9):e0337157. doi: 10.1371/journal.pone.0337157 (PMC13626346; doi:10.1371/journal.pone.0337157)
Supplement: Supplementary file 2 [file pone.0337157.s003.pdf]

Western Blotting Raw Data

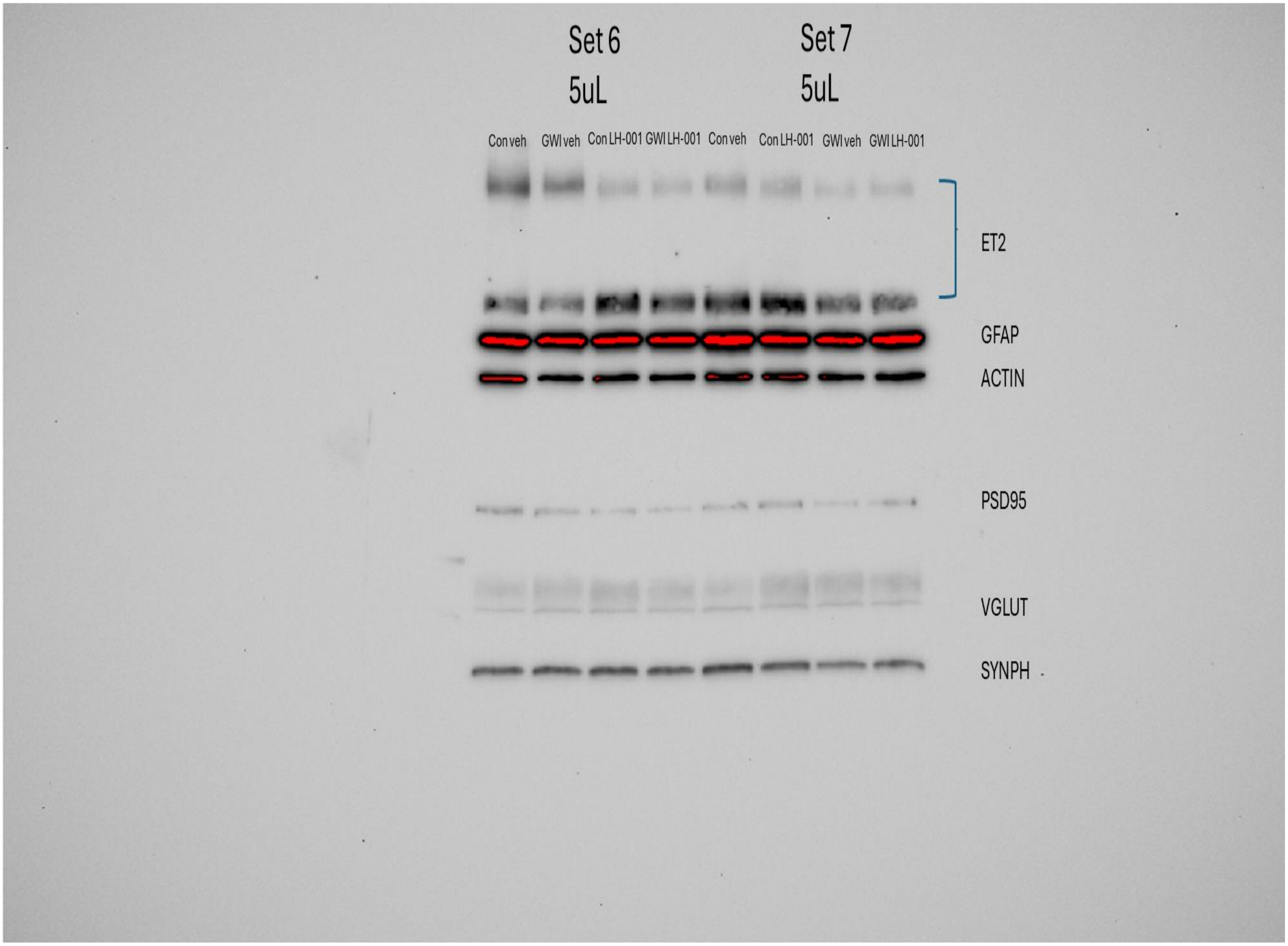

SYP fold change respective to Con Vehicle		
Con LH-001	GWI Veh	GWI LH-001
1.08	0.99	1.25
1.58	0.94	1.22
1.40	1.39	1.09
0.92	0.82	0.92
1.23	1.32	0.94
1.11	0.76	0.44
1.05	0.87	0.89
1.15	1.23	1.34
1.48	1.65	1.56
1.13	1.03	0.95
2.05	2.15	2.29

0.98	1.05	1.22

VGLUT1 fold change respective to Con Vehicle		
Con LH-001	GWI Veh	GWI LH-001
1.01	1.14	1.57
0.96	0.94	2.27
1.47	0.98	1.43
1.61	1.67	1.55
1.15	1.40	1.69
1.39	0.89	0.99
0.81	1.04	1.35
1.47	1.30	1.33
1.94	2.26	2.51
1.21	1.58	0.84
2.83	2.41	2.39
0.92	1.36	0.49

PSD95 fold change respective to Con Vehicle		
Con LH-001	GWI Veh	GWI LH-001
1.42	1.13	1.48
1.43	1.47	1.62
1.12	0.93	1.28
1.24	1.87	1.11
2.20	1.16	0.86
0.83	1.31	1.43
1.29	0.46	0.67
1.12	1.05	2.01
1.17	1.41	0.84
2.25	1.09	0.94
0.88	0.70	1.32
0.80	0.62	0.70

EAAT2 fold change respective to Con Vehicle		
Con LH-001	GWI Veh	GWI LH-001
0.92	1.10	0.93
1.59	0.98	1.28
1.03	1.36	1.21
1.56	1.25	1.52
0.93	1.01	0.83
2.97	0.87	0.89
1.05	1.23	1.34
1.15	1.65	1.56
1.48	1.03	0.95
1.13	1.01	0.83
1.68	1.68	1.65
1.31	1.31	1.42

GFAP fold change respective to Con Vehicle		
Con LH-001	GWI Veh	GWI LH-001
0.96	0.91	0.90
1.13	1.28	1.22
0.79	1.02	1.02
0.97	1.03	1.15
1.44	1.56	1.48
1.38	1.14	1.16
1.14	1.49	1.41
1.46	1.48	1.35
0.71	0.52	0.84
0.74	0.98	0.69
1.22	1.53	1.28
0.55	0.78	0.56

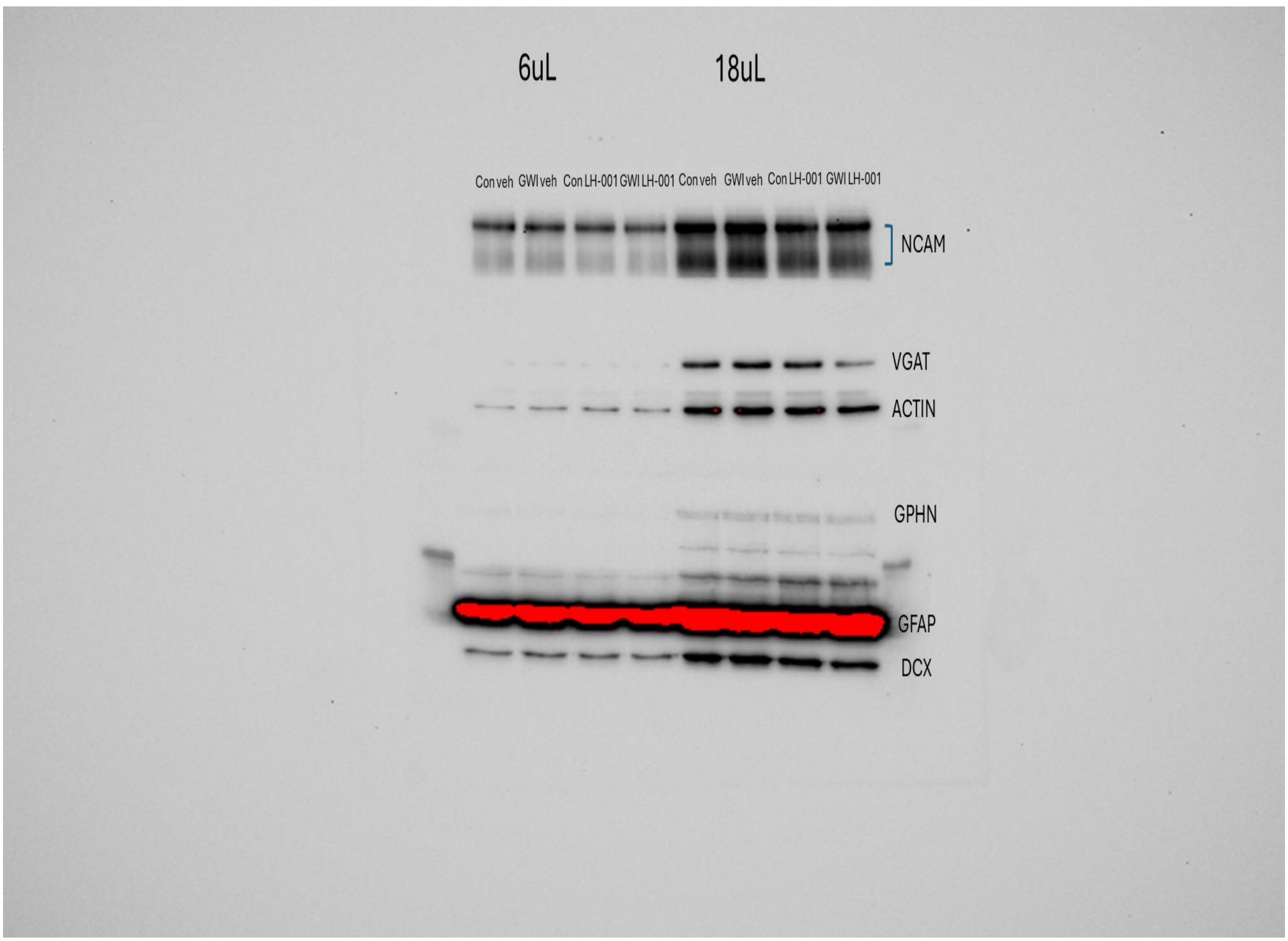

NCAM fold change respective to Con Vehicle		
Con LH-001	GWI Veh	GWI LH-001
0.86	1.04	0.88
1.10	0.85	0.95
1.15	1.00	1.18
1.22	1.38	1.24
1.05	1.08	0.95
1.14	0.92	0.93
1.06	1.15	1.04
1.69	1.83	1.92
1.30	0.93	1.01
1.11	0.96	0.60
1.39	1.53	1.22
0.89	0.98	0.83

VGAT fold change respective to Con Vehicle		
Con LH-001	GWI Veh	GWI LH-001
1.14	1.31	0.99
0.95	0.85	0.49
1.69	1.67	2.39
1.51	0.90	1.96
0.95	1.15	1.95
1.00	0.88	0.67
0.94	1.06	0.58
1.66	0.82	1.34
0.99	0.81	0.69
0.90	1.23	0.59
1.25	1.18	1.24
1.34		1.31

GPHN fold change respective to Con Vehicle		
Con LH-001	GWI Veh	GWI LH-001
1.77	1.63	0.73
0.88	0.84	0.82
1.35	0.78	1.36
1.36	1.00	1.26
1.03	0.86	0.94
1.15	1.27	1.15
1.58	1.58	1.64
1.30	1.59	0.79
0.83	0.89	0.50
0.75	0.70	0.98
0.95	0.74	0.70

DCX fold change respective to Con Vehicle		
Con LH-001	GWI Veh	GWI LH-001
0.95	1.29	1.27
1.06	0.90	0.71
0.94	1.03	0.78
1.24	1.40	0.88
1.22	1.17	1.22
0.94	0.89	0.93
1.10	1.17	1.27
1.75	1.82	1.59
0.97	0.76	0.83
0.87	1.03	0.56
1.12	1.27	1.08
0.80	0.80	0.75
