# Supplementary material for: Limited detection of Gulf War illness-like behavioral phenotypes in Charles River male Sprague-Dawley rats across multiple chemical and stress exposure paradigms
Source: PLoS One. 2026 Sep 30;21(9):e0337157. doi: 10.1371/journal.pone.0337157 (PMC13626346; doi:10.1371/journal.pone.0337157)
Supplement: Supplementary file 3 [file pone.0337157.s004.pdf]

Low-Stress/Low-Dose Study

Test	t/F value	df	P-value	Effect size	95% CI	Mean ± SD
**OFB time in center**	t = 0.0420	46	0.6763	Cohen's d = 0.1212	Control: 4.6402 To 6.8649<br><br>GWI: 3.8587 To 6.8696	Control: 5.7525 ± 2.6341<br><br>GWI: 5.3642 ± 3.5652
**OFB Distance**	t = 0.9207	46	0.3620	Cohen's d = 0.2657	Control: 3599.9235 To 4188.4407<br><br>GWI: 3054.1594 To 4163.3448	Control: 3894.1820 ± 696.8610<br><br>GWI: 3608.7521 ± 1313.3822
**EMPT time in open arm**	t = 0.3867	46	0.7140	Cohen's d = 0.1064	Control: 14.5514 To 23.3336<br><br>GWI: 13.0175 To 22.5125	Control: 18.9400 ± 10.3990<br><br>GWI: 17.7650 ± 11.2430
**Dark and light latency to dark**	t = 0.1625	46	0.8717	Cohen's d = 0.0479	Control: 42.9018 To 76.5182<br><br>GWI: 18.5839 To 94.2065	Control: 59.7100 ± 38.8680<br><br>GWI: 56.3952 ± 87.4386
					Control:	Control:

**Dark and light % time in light**	t = 0.1281	46	0.8997	Cohen's d = -0.0369	14.3049 To 26.4377<br><br>GWI: 11.0782 To 31.1468	20.3713 ± 14.0284<br><br>GWI: 21.1125 ± 23.7631
**Novelty suppressed feeding: % food consumed**	t = 1.6820	46	0.0993	Cohen's d = -0.4856	Control: 3.9289 To 18.0711<br><br>GWI: 11.5559 To 29.2774	Control: 11.0000 ± 16.7457<br><br>GWI: 20.4167 ± 20.9840
**Novelty suppressed Feeding: latency to food**	t = 0.2620	46	0.7946	Cohen's d = 0.0799	Control: 6.9585 To 103.466<br><br>GWI: -8.4405 To 100.0905	Control: 55.2124 ± 106.0071<br><br>GWI: 45.8250 ± 122.3919
***Novel object location: discrimination index**	Treatment: F = 4.0880<br><br>Model: F = 0.6207<br><br>Interaction: F = 0.1363	44	Treatment: 0.0493<br><br>Model: 0.4350<br><br>Interaction: 0.7137	Treatment: ηp^2 = 0.0850<br><br>Model: ηp^2 = 0.0139<br><br>Interaction: ηp^2 = 0.0031	Control: -0.1761 To 0.1207<br><br>Control LH-001: -0.0595 To 0.2267<br><br>GWI Veh: -0.1064 To 0.1074<br><br>GWI LH-001: -0.0017	Control: -0.0277 ± 0.2439<br><br>Control LH-001: 0.0836 ± 0.2283<br><br>GWI Veh: 0.0002 ± 0.1808<br><br>GWI LH-001: 0.1616

					To 0.3248	± 0.2647
**Novel object recognition discrimination index**	t = 1.1120	46	0.2717	Cohen's d = 0.3211	Control: 0.0022 To 0.1694<br><br>GWI: -0.0696 To 0.1075	Control: 0.0858 ± 0.1979<br><br>GWI: 0.01897 ± 0.2097
**Barnes maze trial 1: latency to target hole**	t = 0.0691	46	0.8119	Cohen's d = -0.0691	Control: 7.4468 To 27.2032<br><br>GWI: 9.9593 To 27.8399	Control: 17.3250 ± 23.3936<br><br>GWI: 18.8996 ± 21.1723
***Barnes maze trial 1: % time in target quadrant**	Treatment: F = 0.0939<br><br>Model: F = 0.9360<br><br>Interaction: F = 4.2790	44	Treatment: 0.7607<br><br>Model: 0.3386<br><br>Interaction: 0.0445	Treatment: ηp^2 = 0.0021<br><br>Model: ηp^2 = 0.0208<br><br>Interaction: ηp^2 = 0.0886	Control: 35.7701 To 54.7699<br><br>Control LH-001: 48.1810 To 58.9190<br><br>GWI Veh: 38.0689 To 59.0620<br><br>GWI LH-001: 31.3894 To 50.1397	Control: 43.1608 ± 14.5715<br><br>Control LH-001: 53.4842 ± 8.2634<br><br>GWI Veh: 48.7267 ± 16.2653<br><br>GWI LH-001: 41.3517 ± 14.9885
					Control: 9.4934 To	Control: 16.6450 ±

**Barnes maze trial 2: Latency to target hole**	t = 0.0837	46	0.9337	Cohen's d = -0.0242	23.7966<br><br>GWI: 6.9339 To 27.3877	16.9363<br><br>GWI: 17.1608 ± 24.2193
**Barnes maze trial 2: % time in target quadrant**	t = 1.4960	46	0.1414	Cohen's d = 0.4320	Control: 41.2568 To 53.8682<br><br>GWI: 34.7945 To 47.2396	Control: 47.5625 ± 14.9331<br><br>GWI: 41.0171 ± 14.7362
**Barnes maze trial 2: time in previous target hole**	t = 0.5111	46	0.6117	Cohen's d = -0.1476	Control: 25.6074 To 35.1051<br><br>GWI: 25.5372 To 39.4211	Control: 30.3563 ± 11.2462<br><br>GWI: 32.4792 ± 16.4398

* 2-way Anova Statistical data. All others refer to unpaired t-tests. Only 2-way ANOVA that showed a significant main effect or interaction are included in this table. All other data refers to combined data analyzed by unpaired t-test.

High-Dose/ Low-Stress Study

Test	F value	df	P-value	Effect size	95% CI	Mean ± SD
**OFB time in center**	F = 0.8785	32	0.4252	η^2 = 0.0520	Control: 3.3608 To 10.0626<br><br>GWI Veh: 4.0422 To 8.2724<br><br>GWI LH-001: 3.6352 To 5.7808	Control: 7.9475 ± 5.2895<br><br>GWI Veh: 6.1573 ± 3.1484<br><br>GWI LH-001: 5.7808 ± 3.3770
**OFB Distance**	F = 0.3534	32	0.7050	η^2 = 0.0216	Control: 1391.2217 To 4433.3353<br><br>GWI Veh: 3904.5220 To 4790.6726<br><br>GWI LH-001: 3181.1508 To 3955.3367	Control: 3990.2600 ± 1463.3760<br><br>GWI Veh: 4347.5973 ± 659.5255<br><br>GWI LH-001: 3955.3367 ± 1218.4809
**EMPT time in open arm**	F = 0.3754	23	0.6916	η^2 = 0.0345	Control: 2.9657 To 17.7811<br><br>GWI Veh: 2.4352 To 15.8098	Control: 11.0938 ± 9.7222<br><br>GWI Veh: 9.1225 ± 7.9990

					GWI LH-001: 5.9511 To 13.2113	GWI LH-001: 13.2113 ± 8.6842
**Dark and light latency to dark**	F = 1.325	32	0.2800	η^2= 0.0765	Control: 15.7089 To 41.7749<br><br>GWI Veh: 13.9756 To 43.2004<br><br>GWI LH-001: 23.6937 To 43.8488	Control: 27.1625 ± 18.0266<br><br>GWI Veh: 28.5880 ± 26.3865<br><br>GWI LH-001: 43.8488 ± 24.1083
**Dark and light % time in light**	F = 3.553	32	0.0404	η^2 = 0.1817	Control: 6.2073 To 20.1961<br><br>GWI Veh: 21.0040 To 35.0280<br><br>GWI LH-001: 8.2060 To 24.3813	Control: 13.1842 ± 10.9808<br><br>GWI Veh: 28.0160 ± 12.6620<br><br>GWI LH-001: 24.3813 ± 19.3479
**Novelty suppressed feeding: % food consumed**	F = 4.697	32	0.0163	η^2 = 0.2269	Control: 6.6821 To 38.1825<br><br>GWI Veh: 29.0221 To 65.8870	Control: 19.75 ± 20.5675<br><br>GWI Veh: 47.4546 ± 27.4371

					GWI LH-001: 32.3265 To 50.9167	GWI LH-001: 50.9167 ± 29.2588
**Novelty suppressed Feeding: latency to food**	F = 0.4480	32	0.6431	η^2 = 0.0272	Control: -2.3293 To 22.8387<br><br>GWI Veh: -5.4351 To 28.3914<br><br>GWI LH-001: 0.0016 To 4.9045	Control: 5.9255 ± 12.2874<br><br>GWI Veh: 11.4782 ± 25.1757<br><br>GWI LH-001: 4.9045 ± 7.2981
**Novel object location: discrimination index**	F = 0.0406	32	0.9603	η^2 = 0.0025	Control: -0.1157 To 0.1736<br><br>GWI Veh: -0.0865 To 0.1472<br><br>GWI LH-001: -0.0667 To 0.1600	Control: 0.0568 ± 0.2715<br><br>GWI Veh: 0.0303 ± 0.1740<br><br>GWI LH-001: 0.0467 ± 0.1784
**Novel object recognition discrimination index**	F = 0.4049	32	0.6704	η^2 = 0.0247	Control: -0.1031 To 0.1684<br><br>GWI Veh: -0.0101 To 0.2358	Control: 0.0455 ± 0.2338<br><br>GWI Veh: 0.1128 ± 0.1830

					GWI LH-001: -0.0099 To 0.2431	GWI LH-001: 0.1166 ± 0.1991
**Barnes maze trial 1: latency to target hole**	F = 4.6390	21	0.0214	η² = 0.3064	Control: 4.5974 To 18.0508<br><br>GWI Veh: 9.4904 To 23.4096<br><br>GWI LH-001: 1.6280 To 5.0738	Control: 11.0913 ± 7.7675<br><br>GWI Veh: 16.4500 ± 8.3246<br><br>GWI LH-001: 5.0738 ± 4.1217
**Barnes maze trial 1: % time in target quadrant**	F = 0.1449	21	0.8660	η² = 0.0136	Control: 27.4021 To 45.3224<br><br>GWI Veh: 36.4914 To 45.2486<br><br>GWI LH-001: 32.4630 To 44.0663	Control: 40.9438 ± 16.1978<br><br>GWI Veh: 40.8700 ± 5.2375<br><br>GWI LH-001: 44.0663 ± 13.8791
**Barnes maze trial 2: Latency to target hole**	F = 2.6430	21	0.0947	η² = 0.2011	Control: 13.6356 To 55.0990<br><br>GWI Veh: 4.2260 To 36.2765	Control: 39.0738 ± 30.4276<br><br>GWI Veh: 20.2513 ± 19.1685

					GWI LH-001: 5.2345 To 13.5425	GWI LH-001: 13.5425 ± 9.9376
**Barnes maze trial 2: % time in target quadrant**	F = 3.6240	21	0.0445	η^2 = 0.2566	Control: 19.1107 To 38.5037<br><br>GWI Veh: 39.5726 To 54.6224<br><br>GWI LH-001: 26.8871 To 35.8125	Control: 30.9788 ± 14.1958<br><br>GWI Veh: 47.0975 ± 9.0009<br><br>GWI LH-001: 35.8125 ± 10.6760
**Barnes maze trial 2: time in previous target hole**	F = 0.7494	21	0.4849	η^2 = 0.0666	Control: 21.0274 To 45.7452<br><br>GWI Veh: 10.7160 To 37.9490<br><br>GWI LH-001: 19.6701 To 25.5775	Control: 32.1288 ± 13.2788<br><br>GWI Veh: 24.3325 ± 16.2872<br><br>GWI LH-001: 25.5775 ± 7.0660

All statistical data refers to one-way ANOVA results

High-Stress/Low-Dose

Test	t/F value	df	P-value	Effect size	95% CI	Mean ± SD
***OFB time in center**	Treatment: F = 0.1727<br><br>Model: F = 0.1131<br><br>Interaction: F = 4.6820	44	Treatment: 0.6797<br><br>Model: 0.7382<br><br>Interaction: 0.0360	Treatment: ηp^2 = 0.0039<br><br>Model: ηp^2 = 0.0026<br><br>Interaction: ηp^2 = 0.0962	Control: 3249.0624 To 4209.4893<br><br>Control LH-001: 2796.0755 To 4289.1729<br><br>GWI Veh: 3070.4527 To 4063.5839<br><br>GWI LH-001: 2607.4826 To 3963.1958	Control: 3729.2758 ± 755.8016<br><br>Control LH-001: 3542.6242 ± 1174.9832<br><br>GWI Veh: 3567.0183 ± 781.5381<br><br>GWI LH-001: 3285.3392 ± 1066.8696
**OFB Distance**	t = 0.7339	46	0.4667	Cohen's d = 0.3129	Control: 3216.9467 To 4054.9533<br><br>GWI: 3026.8461 To 3825.5114	Control: 3635.9500 ± 992.2805<br><br>GWI: 3426.1788 ± 945.6966
***EMPT time in open arm**	Treatment: F = 0.2283<br><br>Model: F = 6.6250<br><br>Interaction: F = 6.5420	44	Treatment: 0.6351<br><br>Model: 0.0135<br><br>Interaction: 0.0141	Treatment: ηp^2 = 0.0052<br><br>Model: ηp^2 = 0.1309<br><br>Interaction:	Control: 1485.7446 To 5972.8071<br><br>Control LH-001: 2796.0755 To	Control: 3729.2758 ± 755.8016<br><br>Control LH-001: 3542.6242 ±

				ηp^2 = 0.1295	4289.1729<br><br>GWI Veh: 3070.4527 To 4063.5839<br><br>GWI LH-001: 2607.4826 To 3963.1958	1174.9832<br><br>GWI Veh: 3567.0183 ± 781.5381<br><br>GWI LH-001: 3285.3392 ± 1066.8696
**Dark and light latency to dark**	t= 0.8599	45	0.3969	Cohen's d = 0.2509	Control: 11.9675 To 59.9075<br><br>GWI: 16.2547 To 33.9279	Control: 35.9375 ± 56.7656<br><br>GWI: 25.0913 ± 20.4347
**Dark and light % time in light**	t = 1.0760	46	0.2876	Cohen's d = 0.4588	Control: 12.1152 To 27.0698<br><br>GWI: 17.2214 To 33.8411	Control: 19.5925 ± 17.7076<br><br>GWI: 25.5313 ± 19.6792
**Novelty suppressed feeding: % food consumed**	t = 0.8174	46	0.4179	Cohen's d = 0.3485	Control: 21.8483 To 41.7350<br><br>GWI: 28.1962 To 46.2204	Control: 31.7917 ± 23.5478<br><br>GWI: 37.2083 ± 21.3424
**Novelty suppressed Feeding: latency to food**	t = 0.9735	45	0.3381	Cohen's d = 0.2841	Control: 5.2105 To 39.5504	Control: 22.3804 ± 40.6618

					GWI: 6.1855 To 20.6649	GWI: 13.4252 ± 16.7419
**Novel object location: discrimination index**	t = 0.2931	45	0.7708	Cohen's d = 0.1250	Control: -0.0745 To 0.1538<br><br>GWI Veh: -0.1432 To 0.1671	Control: 0.0396 ± 0.2704<br><br>GWI Veh: 0.0119 ± 0.3588
**Novel object recognition discrimination index**	t = 0.1889	45	0.8510	Cohen's d = 0.0806	Control: -0.0146 To 0.1099<br><br>GWI: -0.0372 To 0.1536	Control: 0.0477 ± 0.1475<br><br>GWI: 0.0582 ± 0.2206
***Barnes maze trial 1: latency to target hole**	Treatment: F = 4.031<br><br>Model: F = 4.353<br><br>Interaction: F= 0.4306	44	Treatment: 0.0508<br><br>Model: 0.0428<br><br>Interaction: 0.5825	Treatment: ηp^2 = 0.0839<br><br>Model: ηp^2 = 0.0900<br><br>Interaction: ηp^2 = 0.0097	Control: 2.0329 To 34.0237<br><br>Control LH-001: 13.1904 To 36.8046<br><br>GWI Veh: 1.5555 To 6.8595<br><br>GWI LH-001: 10.1622 To 25.0961	Control: 18.0283 To 25.1750<br><br>Control LH-001: 24.9975 To 18.5831<br><br>GWI Veh: 4.2075 To 4.1740<br><br>GWI LH-001: 17.6292 To 11.7521

**Barnes maze trial 1: % time in target quadrant**	t = 0.6643	46	0.5098	Cohen's d = 0.2833	Control: 45.5482 To 56.1093<br><br>GWI Veh: 47.2173 To 59.8386	Control: 50.8288 ± 12.5053<br><br>GWI Veh: 53.5279 ± 14.9449
**Barnes maze trial 2: Latency to target hole**	t = 1.541	46	0.1325	Cohen's d = 0.6571	Control: 7.1572 To 21.4412<br><br>GWI: 4.5503 To 11.8431	Control: 14.2992 ± 16.9136<br><br>GWI: 8.1967 ± 8.6354
**Barnes maze trial 2: % time in target quadrant**	t = 0.2812	46	0.7796	Cohen's d = 0.1199	Control: 47.8325 To 59.3575<br><br>GWI: 49.9433 To 59.3092	Control: 53.5950 ± 13.6466<br><br>GWI: 54.6263 ± 11.0902
**Barnes maze trial 2: time in previous target hole**	t = 0.0196	46	0.9844	Cohen's d = 0.0084	Control: 27.1676 To 41.5357<br><br>GWI: 27.0276 To 41.4782	Control: 34.3517 ± 17.0132<br><br>GWI: 34.2529 ± 17.1108

* 2-way Anova Statistical data. All others refer to unpaired t-tests. Only 2-way ANOVA that showed a significant main effect or interaction are included in this table. All other data refers to combined data analyzed by unpaired t-test.

IHC Statistics

Test	t/F value	df	P-value	Effect size	95% CI	Mean ± SD
*GFAP – DG	Treatment: F = 1.0970<br><br>Model: F = 0.2039<br><br>Interaction: F = 1.1480	116	Treatment: 0.2970<br><br>Model: 0.6524<br><br>Interaction: 0.2862	Treatment: ηp^2 = 0.0094<br><br>Model: ηp^2 = 0.0018<br><br>Interaction: ηp^2 = 0.0098	Control: 31.3107 To 49.8319<br><br>Control LH-001: 40.9305 To 56.1369<br><br>GWI Veh: 35.0193 To 48.2617<br><br>GWI LH-001: 36.7426 To 49.1370	Control: 40.5713 ± 24.8116<br><br>Control LH-001: 48.5337 ± 20.3695<br><br>GWI Veh: 41.6405 ± 16.7418<br><br>GWI LH-001: 42.9398 ± 17.7326
*GFAP – CA1	Treatment: F = 1.0060<br><br>Model: F = 4.8810<br><br>Interaction: F = 0.0454	128	Treatment: 0.3177<br><br>Model: 0.0289<br><br>Interaction: 0.8316	Treatment: ηp^2 = 0.0078<br><br>Model: ηp^2 = 0.0367<br><br>Interaction: ηp^2 = 0.0004	Control: 10.5169 To 14.8139<br><br>Control LH-001: 7.2805 To 13.1263<br><br>GWI Veh: 12.3802 To 17.4376<br><br>GWI LH-001: 10.9735	Control: 12.6654 ± 6.1543<br><br>Control LH-001: 10.2034 ± 8.2431<br><br>GWI Veh: 14.9089 ± 6.8984<br><br>GWI LH-001: 13.4363

					To<br>15.8991	±<br>7.0512
***GFAP – CA3**	Treatment: F = 0.5729<br><br>Model: F = 4.883<br><br>Interaction: F = 1.8420	131	Treatment: 0.4505<br><br>Model: 0.0289<br><br>Interaction: 0.1770	Treatment: ηp^2 = 0.0045<br><br>Model: ηp^2 = 0.0367<br><br>Interaction: ηp^2 = 0.0142	Control: 14.4096 To 22.3250<br><br>Control LH-001: 13.3522 To 21.0250<br><br>GWI Veh: 10.1926 To 14.0146<br><br>GWI LH-001: 12.6038 To 18.6170	Control: 18.3673 ± 11.4896<br><br>Control LH-001: 17.1886 ± 10.8254<br><br>GWI Veh: 12.1036 ± 5.2178<br><br>GWI LH-001: 15.6104 ± 8.9057
***PV – HC**	Treatment: F = 0.6919<br><br>Model: F = 2.7610<br><br>Interaction: F = 0.1349	131	Treatment: 0.4077<br><br>Model: 0.0999<br><br>Interaction: 0.7142	Treatment: ηp^2 = 0.0053<br><br>Model: ηp^2 = 0.00206<br><br>Interaction: ηp^2 = 0.0010	Control: 13.6718 To 17.1500<br><br>Control LH-001: 14.3326 To 20.4506<br><br>GWI Veh: 15.7557 To 21.2217<br><br>GWI LH-001: 16.3718 To 22.7030	Control: 15.4109 ± 4.2138<br><br>Control LH-001: 17.3916 ± 6.8951<br><br>GWI Veh: 18.4887 ± 6.7782<br><br>GWI LH-001: 19.5374 ± 7.4978

***DCX – DG**	Treatment: F = 0.2535<br><br>Model: F = 11.2800<br><br>Interaction: F = 2.7590	125	Treatment: 0.6155<br><br>Model: 0.0010<br><br>Interaction: 0.0992	Treatment: ηp^2 = 0.0020<br><br>Model: ηp^2 = 0.0828<br><br>Interaction: ηp^2 = 0.0216	Control: 14.8689 To 21.1843<br><br>Control LH-001: 17.1088 To 25.2264<br><br>GWI Veh: 13.2206 To 17.8226<br><br>GWI LH-001: 11.5800 To 15.5700	Control: 18.0266 ± 8.9007<br><br>Control LH-001: 21.1676 ± 12.5264<br><br>GWI Veh: 15.5216 ± 5.9287<br><br>GWI LH-001: 13.5750 ± 5.2721

* 2-way Anova Statistical data

Biochemical Statistics

Test	t value	df	P-value	Effect size Cohen's d	95% CI	Mean ± SD
**SYP**	Control LH-001: t = 2.862<br><br>GWI Veh: t = 1.586<br><br>GWI LH-001: t = 1.353	11	Control LH-001: 0.0155<br><br>GWI Veh: 0.2435<br><br>GWI LH-001: 0.2738	Control LH-001: 0.8630<br><br>GWI Veh: 0.4783<br><br>GWI LH-001: 0.4081	Control LH-001: 1.0695 To 1.4572<br><br>GWI Veh: 0.9398 To 1.4269<br><br>GWI LH-001: 0.9021 To 1.4496	Control LH-001: 1.2633 ± 0.3051<br><br>GWI Veh: 1.1833 ± 0.3833<br><br>GWI LH-001: 1.1758 ± 0.4309
**VGLUT1**	Control LH-001: t = 2.4720<br><br>GWI Veh: t = 2.8850<br><br>GWI LH-001: t = 2.9920	11	Control LH-001: 0.0310<br><br>GWI Veh: 0.0148<br><br>GWI LH-001: 0.0122	Control LH-001: 0.7453<br><br>GWI Veh: 0.8700<br><br>GWI LH-001: 0.9022	Control LH-001: 1.0586 To 1.7364<br><br>GWI Veh: 1.1117 To 1.7166<br><br>GWI LH-001: 1.1580 To 1.9104	Control LH-001: 1.3975 ± 0.5333<br><br>GWI Veh: 1.4142 ± 0.4761<br><br>GWI LH-001: 1.5342 ± 0.5921
**PSD95**	Control LH-001: t = 2.2820	11	Control LH-001: 0.0434	Control LH-001: 0.6882	Control LH-001: 1.0240 To 1.6010	Control LH-001: 1.3125 ± 0.4541

	GWI Veh: t = 0.8796<br><br>GWI LH-001: t = 1.5970		GWI Veh: 0.3979<br><br>GWI LH-001: 0.1385	GWI Veh: 0.2652<br><br>GWI LH-001: 0.4816	GWI Veh: 0.8604 To 1.3396<br><br>GWI LH-001: 0.9399 To 1.4368	GWI Veh: 1.1000 ± 0.3770<br><br>GWI LH-001: 1.1883 ± 0.3910
**NCAM**	Control LH-001: t = 2.5340<br><br>GWI Veh: t = 1.6180<br><br>GWI LH-001: t = 0.6688	11	Control LH-001: 0.0278<br><br>GWI Veh: 0.1340<br><br>GWI LH-001: 0.5174	Control LH-001: 0.7640<br><br>GWI Veh: 0.4878<br><br>GWI LH-001: 0.2016	Control LH-001: 1.0275 To 1.2992<br><br>GWI Veh: 0.9584 To 1.3166<br><br>GWI LH-001: 0.8656 To 1.2594	Control LH-001: 1.1633 ± 0.2138<br><br>GWI Veh: 1.1375 ± 0.2819<br><br>GWI LH-001: 1.0625 ± 0.3099
**EAAT2**	Control LH-001: t = 2.6880<br><br>GWI Veh: t = 2.3350<br><br>GWI LH-001: t = 2.2280	11	Control LH-001: 0.0211<br><br>GWI Veh: 0.0395<br><br>GWI LH-001: 0.0429	Control LH-001: 0.8105<br><br>GWI Veh: 0.7041<br><br>GWI LH-001: 0.6899	Control LH-001: 1.0974 To 1.8043<br><br>GWI Veh: 1.0352 To 1.6864<br><br>GWI LH-001: 1.0299 To 1.7284	Control LH-001: 1.4508 ± 0.5563<br><br>GWI Veh: 1.3608 ± 0.5125<br><br>GWI LH-001: 1.3792 ± 0.5496
**GFAP**	Control LH-001: t = 0.4656	11	Control LH-001: 0.6506	Control LH-001: 0.1404	Control LH-001: 0.8560	Control LH-001: 1.0408

	GWI Veh:<br>t = 1.5000<br><br>GWI<br>LH-001:<br>t = 1.0540		GWI Veh:<br>0.1617<br><br>GWI LH-001:<br>0.3147	GWI Veh:<br>0.4523<br><br>GWI LH-001:<br>0.3177	To<br>1.2256<br><br>GWI Veh:<br>0.9420<br>To<br>1.3447<br><br>GWI<br>LH-001:<br>0.9116<br>To<br>1.2650	±<br>0.2909<br><br>GWI Veh:<br>1.1433<br>±<br>0.3169<br><br>GWI<br>LH-001:<br>1.0883<br>±<br>0.2781
**VGAT**	Control<br>LH-001:<br>t = 2.2910<br><br>GWI Veh:<br>t = 0.9769<br><br>GWI<br>LH-001:<br>t = 1.001	11<br><br>10<br><br>11	Control<br>LH-001:<br>0.0427<br><br>GWI Veh:<br>0.3517<br><br>GWI LH-001:<br>0.3386	Control<br>LH-001:<br>0.6909<br><br>GWI Veh:<br>0.3089<br><br>GWI LH-001:<br>0.3017	Control<br>LH-001:<br>1.0155<br>To<br>1.3711<br><br>GWI Veh:<br>0.9082<br>To<br>1.2482<br><br>GWI<br>LH-001:<br>0.7972<br>To<br>1.5695	Control<br>LH-001:<br>1.1933<br>±<br>0.2798<br><br>GWI Veh:<br>1.0782<br>±<br>0.2531<br><br>GWI<br>LH-001:<br>1.1833<br>±<br>0.6077
**GPHN**	Control<br>LH-001:<br>t = 1.8080<br><br>GWI Veh:<br>t = 0.7229<br><br>GWI<br>LH-001:<br>t = 0.1171	10	Control<br>LH-001:<br>0.1007<br><br>GWI Veh:<br>0.4863<br><br>GWI LH-001:<br>0.9091	Control<br>LH-001:<br>0.5718<br><br>GWI Veh:<br>0.2286<br><br>GWI LH-001:<br>-0.0370	Control<br>LH-001:<br>0.9690<br>To<br>1.3856<br><br>GWI Veh:<br>0.8449<br>To<br>1.3151<br><br>GWI<br>LH-001:<br>0.7737<br>To<br>1.2027	Control<br>LH-001:<br>1.1773<br>±<br>0.3100<br><br>GWI Veh:<br>1.0800<br>±<br>0.3500<br><br>GWI<br>LH-001:<br>0.9882<br>±<br>0.3193

**DCX**	Control LH-001: t = 1.1090<br><br>GWI Veh: t = 1.4850<br><br>GWI LH-001: t = 0.1255	11	Control LH-001: 0.2912<br><br>GWI Veh: 0.1657<br><br>GWI LH-001: 0.9024	Control LH-001: 0.3343<br><br>GWI Veh: 0.4476<br><br>GWI LH-001: -0.0378	Control LH-001: 0.9280 To 1.2320<br><br>GWI Veh: 0.9465 To 1.3085<br><br>GWI LH-001: 0.8072 To 1.1711	Control LH-001: 1.0800 ± 0.2393<br><br>GWI Veh: 1.1275 ± 0.2848<br><br>GWI LH-001: 0.9892 ± 0.2864

All data refers to two-tailed t-test.
