# Supplementary material for: Limited detection of Gulf War illness-like behavioral phenotypes in Charles River male Sprague-Dawley rats across multiple chemical and stress exposure paradigms
Source: PLoS One. 2026 Sep 30;21(9):e0337157. doi: 10.1371/journal.pone.0337157 (PMC13626346; doi:10.1371/journal.pone.0337157)
Supplement: Supplementary file 4 [file pone.0337157.s005.docx]

**S5. Analysis of pooled data shows no significant differences between Control and GWI groups under low-dose/low-stress exposure.**

For tests in Figure 1 in which two-way ANOVA revealed no treatment effects or interactions, control/vehicle and control/LH-001 groups were combined as "Control", and GWI/vehicle and GWI/LH-001 groups were combined as "GWI". Unpaired t-tests were used to compare the combined groups. Unless otherwise indicated, n = 24 per group. **(A, B)** Open-field test: time spent in the center (A) and total distance traveled (B). **(C)** Elevated plus maze: percentage of time spent in the open arms. **(D, E)** Light–dark transition test: latency to enter the dark compartment (D) and percentage of time spent in the light compartment (E) (n = 23–24/group). **(F, G)** Novelty-suppressed feeding test: food consumption (F) and latency to feed (G) (n = 20–24/group). **(H)** Novel object recognition: discrimination index. **(I)** Barnes maze training: latency to find the escape hole. **(J, K)** Barnes maze re-training: latency to locate the new escape hole (J) and time spent in the new target quadrant (K) one week later. **(L)** Barnes maze memory recall: time spent in the original target quadrant during the re-training probe. No significant differences were observed between the combined "Control" and "GWI" groups in any behavioral measure.
