# Supplementary material for: Limited detection of Gulf War illness-like behavioral phenotypes in Charles River male Sprague-Dawley rats across multiple chemical and stress exposure paradigms
Source: PLoS One. 2026 Sep 30;21(9):e0337157. doi: 10.1371/journal.pone.0337157 (PMC13626346; doi:10.1371/journal.pone.0337157)
Supplement: Supplementary file 5 [file pone.0337157.s006.docx]

**S6. Analysis of pooled data shows no significant differences between Control and GWI groups under low-dose/high-stress exposure.**

For tests in Figure 3 in which two-way ANOVA revealed no treatment effects or interactions, control/vehicle and control/LH-001 groups were combined as "Control" and GWI/vehicle and GWI/LH-001 groups were combined as "GWI". Unpaired t-tests were used to compare the combined groups. Unless otherwise indicated, n = 24 per group. **(A) Open-field test**: time spent in the center. **(B–C) Dark–light test**: latency to enter the dark chamber (B) and percentage of time spent in the light chamber (C) (n = 23–24/group). **(D–E) Novelty-suppressed feeding test**: latency to begin feeding (D) and food consumption (E) (n = 23–24/group). **(F–G) Recognition memory test**: discrimination index for object recognition tasks (n = 23–24/group). **(H) Barnes maze**: time spent in the target quadrant during the initial training phase. **(I–J) Barnes maze re-training**: latency to locate the new escape hole (I) and time spent in the new target quadrant (J), assessed one week later. **(K) Barnes maze memory recall**: time spent in the original target quadrant. No significant differences were observed between groups.
